# Cloning, structural and functional characterization of the ABCB1 transporter of the Eurasian bullfinch (*Pyrrhula pyrrhula*)

**DOI:** 10.3389/fvets.2026.1759019

**Published:** 2026-04-10

**Authors:** Katharina Heilmann, Luise Charlotte Kempf, Lisa Siegl, Melanie Hamann, Michael Lierz, Joachim Geyer

**Affiliations:** 1 Faculty of Veterinary Medicine, Biomedical Research Center Seltersberg (BFS), Institute of Pharmacology and Toxicology, Justus Liebig University of Giessen, Giessen, Germany; 2 Faculty of Veterinary Medicine, Clinic for Birds, Reptiles, Amphibians and Fish, Justus Liebig University of Giessen, Giessen, Germany

**Keywords:** ABCB1, bullfinch, drug transport and binding, efflux, ivermectin, P-glycoprotein

## Abstract

Suspected ivermectin hypersensitivity has been reported in the Eurasian bullfinch and some related passerine birds. However, the underlying cause and pharmacological mechanism have not been systematically investigated. In mammals, ivermectin hypersensitivity in dogs and cats has been linked to mutations in the *ABCB1* gene, which encodes the P-glycoprotein drug efflux transporter. In the present study, the full-length ABCB1 transcript of the Eurasian bullfinch (*Pyrrhula pyrrhula*) was amplified and cloned. Sequence analysis identified a tandem-repeat insertion consisting of two identical 10-amino acid motifs within the ABCB1 linker region, a feature absent from human and chicken ABCB1. A systematic analysis of this insertion motif across available ABCB1 sequences from passerine birds identified four distinct variants: a single-motif insertion, a tandem repeat insertion, a triple repeat insertion, or complete absence of the insertion. These variants showed a predominant, though not exclusive, association with specific *Passeriformes* families. Heterologous expression of Eurasian bullfinch ABCB1 in HEK293 cells revealed significantly reduced total protein levels of the wild-type ABCB1 transporter compared with a mutant lacking the tandem repeat insertion. However, efflux activity toward the fluorescent ABCB1 substrate Rhodamine 123 did not differ between the wild-type and mutant transporters. Likewise, inhibition of Rhodamine 123 efflux by the established ABCB1 inhibitors ivermectin and tariquidar was comparable between the Eurasian bullfinch ABCB1 variants and with canine ABCB1. In conclusion, although a distinct genetic feature was identified in the ABCB1 linker region of some passerine birds, no functional differences in Rhodamine 123 efflux or inhibitor sensitivity were detected. Our *in vitro* data do not support a role of this ABCB1 insertion for the suspected ivermectin hypersensitivity in the Eurasian bullfinch.

## Introduction

Adverse events, especially symptoms of neurological toxicity, are often reported after administration of veterinary antiparasitic drugs (1–5). Some of them have been associated with mutations in the ABCB1 drug efflux transporter (syn. P-glycoprotein) that is highly expressed at the blood-brain barrier (BBB) and normally restricts the entry of many clinically used drugs into the central nervous system (6–15). The ABCB1 transporter phylogenetically belongs to the ATP-binding cassette (ABC) transporter superfamily (16, 17) and is highly conserved in all vertebrates (18). The significant role of ABCB1 for drug brain penetration and for preventing neurological toxicity of potentially neurotoxic drugs has been well studied in *Abcb1-*deficient mouse models (19). As an example, *Abcb1*-deficient mice revealed dramatically increased sensitivity to the centrally neurotoxic pesticide ivermectin (IVM), associated with 59- to 87-fold higher drug concentrations in the brain compared to *Abcb1* intact control mice (19, 20). Some years later, gene deletion mutations in the *ABCB1* gene have been identified in dogs and cats that have also been associated with dramatically increased drug sensitivity against IVM and some other drugs (6, 10, 21, 22). In birds, the orange-cheeked waxbill (*Estrilda melpoda*, Vieillot, 1817), the European goldfinch (*Carduelis carduelis*, Linnaeus, 1758), and the Eurasian bullfinch (*Pyrrhula pyrrhula*, Linnaeus, 1758) have been reported to be more sensitive to IVM than other bird species, suggesting a genetic background (23–26). This encouraged us in the present study to clone and characterize the ABCB1 transporter from finches, using the Eurasian bullfinch as the model species.

## Materials and methods

### Materials

Stock solutions of the reagents, used in the transport assay, were prepared in 100% dimethyl sulfoxide (DMSO, Carl Roth, Karlsruhe, Germany). The fluorescent substrate Rhodamine 123 (Rh123) was used at a stock concentration of 10 mM and was obtained from Abcam (Cambridge, UK). The ABCB1 control inhibitor tariquidar (TQR) was purchased from Sigma-Aldrich (St. Louis, MO, USA). IVM was obtained from HPC Standards (Cunnersdorf, Germany).

### Cloning and sequencing of the Eurasian bullfinch ABCB1 transporter

Brain tissue of a Eurasian bullfinch was obtained at the Clinic for Birds, Reptiles, Amphibians and Fish of the Justus Liebig University Giessen and was preserved in RNAlater solution according to the manufacturer's instructions (Qiagen, Hilden, Germany). Severely injured wild birds found in the wild are frequently admitted to this clinic for treatment and rehabilitation. In accordance with animal welfare principles, wild birds suffering from severe injuries and a hopeless prognosis are humanely euthanized. RNA was isolated from the RNAlater-preserved brain tissue using TRI Reagent RNA Isolation Reagent according to the manufacturer's standard protocol (Sigma-Aldrich). Then, the RNA sample was subjected to standard DNase I digestion (Fermentas, St. Leon-Rot, Germany), and the SuperScript III Reverse Transcriptase was used for cDNA synthesis with random hexamers according to the manufacturer's standard protocol (Invitrogen, Carlsbad, CA, USA). As no Eurasian bullfinch-specific ABCB1 sequence was known at that time, oligonucleotide primers were derived from the ABCB1 sequence of the chicken (GenBank accession number NM_204894.2), organized in four overlapping fragments covering the full open reading frame (ORF) as indicated in Table 1. PCR amplification of the Eurasian bullfinch cDNA was performed with the FastStart High Fidelity PCR System (Roche, Mannheim, Germany) on a Primus 96 advanced gradient PCR cycler (Peqlab, Erlangen, Germany) under the following conditions: initial denaturation for 2 min at 95 °C; 35 cycles of denaturation for 30 s at 95 °C, annealing for 1 min at 5 °C < mean T_m_ of the respective primer pair, and extension for 1 min at 72 °C; final elongation for 7 min at 72 °C; and a final hold at 4 °C. PCR products were visualized by agarose gel electrophoresis and were sequenced (Microsynth Seqlab, Göttingen, Germany). To additionally obtain the Eurasian bullfinch ABCB1 sequences upstream of the start codon and downstream of the stop codon, 3′- and 5′-rapid amplification of cDNA ends (RACE) reactions were performed using the gene-specific forward primer 5′-cgc ctc tcc acc atc cag aac gct gac-3′ for the 3′-RACE reaction and the reverse primer 5′-gat tca cca cac caa taa cct ccc gca gat acc-3′ for the 5′-RACE reaction. RACE-PCR was performed with the SMARTer RACE cDNA Amplification Kit (Clontech, Mountain View, CA, USA) as previously reported (27) using the manufacturer's standard protocol. PCR amplification was performed with the FastStart High Fidelity DNA polymerase (Roche) under the following touch-down thermocycling conditions on a Peqlab Primus 96 advanced gradient PCR cycler: initial denaturation at 95 °C for 2 min; 10 cycles of 95 °C for 30 s, 72 °C minus 0.5 °C each cycle for 30 s, and 72 °C for 1 min; 35 cycles of 95 °C for 30 s, 67 °C for 30 s, and 72 °C for 1 min; and a final elongation at 72 °C for 10 min. The 3′- and 5′-RACE fragments were gel-purified and subjected to DNA sequencing (Microsynth Seqlab). Finally, the full-length Eurasian bullfinch ABCB1 transcript covering the entire ORF was amplified using the Phusion High-Fidelity Polymerase (Thermo Fisher Scientific, Waltham, MA, USA) with the following primers: 5‘-aat tta aaa tgc ctt ctg aag atg ag-3‘ forward and 5‘-gag ata agt act gcc ttg aat t-3‘ reverse. Amplification was carried out on a Peqlab Primus 96 advanced gradient PCR cycler at 35 cycles under the following conditions: initial denaturation for 30 s at 98 °C, denaturation for 10 s at 98 °C, annealing for 30 s at 60 °C, and extension for 2 min at 72 °C; final elongation for 10 min at 72 °C; and a final hold at 4 °C. The resulting PCR product was purified via gel extraction using the HiYield PCR Clean-up and Gel Extraction Kit according to the manufacturer's standard protocol (SLG, Gauting, Germany), and A-overhangs were added using the DyNAzyme II DNA Polymerase (Thermo Fisher Scientific) for subsequent TA-cloning. Finally, the full-length Eurasian bullfinch ABCB1 transcript was cloned into the expression vector pcDNA5/FRT/TO-TOPO (Invitrogen) via TA-cloning as reported before (14, 27, 28). Sequence verification was done by DNA sequencing (Microsynth Seqlab). The full-length Eurasian bullfinch ABCB1 cDNA sequence of 3915 bp was deposited into the GenBank database with accession number HG917089.

**Table 1:** Primers derived from the chicken ABCB1 cDNA (GenBank accession number NM_204894.2) that were used for PCR amplification of the Eurasian bullfinch ABCB1 sequence in four partly overlapping fragments.

Primer	Sequence (5^′^ → 3^′^)	T_m_	Position at NM_204894.2	Product length
F1	caa aca gaa cga ctt gca cgt att a	63 °C	11-35	990 bp
R1	gaa agc agc acc cat aga aat a	58 °C	979-1000	
F2	tat gca aaa gca gga gct gtt g	60 °C	830-851	1,409 bp
R2	gtt cca gct aca aag tat ggc c	62 °C	2217-2238	
F3	cgt cta tca aca gtc cga aat gc	63 °C	1853-1875	1,044 bp
R3	cgc ctt ctt cac aga att tct	57 °C	2876-2896	
F4	gga aag att gcc aca gaa gcc at	63 °C	2771-2793	1,185 bp
R4	gag aga agt act gcc ttt aat t	57 °C	3934-3955	

### BLAST search for ABCB1 sequences from other *Passeriformes* species

Using the full-length cDNA sequence of the Eurasian bullfinch ABCB1 sequence (GenBank accession number HG917089) as the query, a systematic BLAST search was performed with the NCBI blastn tool (https://blast.ncbi.nlm.nih.gov/Blast.cgi) with standard settings. The top 100 hits were manually evaluated, and all identified ABCB1 sequences from *Passeriformes* species are listed in Table 2. As in the BLAST analysis, the European greenfinch (*Chloris chloris*, Linnaeus, 1758) was the only *Passeriformes* species with a triple repeat insertion (see below), additional BLAST analysis was done with the European greenfinch cDNA sequence as the query with GenBank accession number HG917096. Furthermore, BLAST analysis was repeated with the ABCB1 cDNA sequence from Sunda zebra finch (*Taeniopygia guttata*, Vieillot, 1817), as a representative with a single insertion motif (see below). However, in both cases, no additional ABCB1 sequences from *Passeriformes* species could be identified. For all *Passeriformes* ABCB1 protein sequences, additional phylogenetic analysis was performed, where the human ABCB1 protein was added for comparison (GenBank accession number NP_001335873). Redundant sequences were deleted (see Table 2). Multi-sequence alignment was performed with the NCBI constraint-based multiple alignment tool COBALT (www.ncbi.nlm.nih.gov/tools/cobalt/) with standard settings. The resulting tree was edited with the TreeViewer (29).

**Table 2:** ABCB1 sequences from *Passeriformes* species, homologous to the Eurasian bullfinch ABCB1 sequence, retrieved by BLAST search on January 27th 2026.

Species	Family	Trivial name	GenBank (nt)	mRNA (bp)	GenBank (protein)	Protein (aa)	Insertion repeats
*Chloris chloris*	*Fringillidae*	European greenfinch	HG917096	1559	**CDM63417**	519	3
*Pyrrhula pyrrhula*	*Fringillidae*	Eurasian bullfinch	HG917089	3915	**CDM63410**	1304	2
*Haemorhous mexicanus*	*Fringillidae*	House finch	XM_059842676	5644	**XP_059698659**	1305	2
			XM_059842686	3564	XP_059698669	924	
*Serinus canaria*	*Fringillidae*	Atlantic canary	HG917099	3030	CDM63420	998	2
			XM_009085927	6566	XP_009084175	1305	
			XM_050971261	6021	**XP_050827218**	1304	
			XM_018909382	3405	XP_018764927	924	
*Serinus serinus*	*Fringillidae*	European serin	HG917095	2830	**CDM63416**	943	2
*Spinus spinus* *(Carduelis spinus)*	*Fringillidae*	Eurasian siskin	HG917094	2785	**CDM63415**	927	2
*Motacilla alba alba*	*Motacillidae*	White wagtail	XM_038128508	5528	**XP_037984436**	1304	2
			XM_038128509	3387	XP_037984437	923	
*Pyrgilauda ruficollis*	*Passeridae*	Rufous-necked snowfinch	XM_041466407	5519	XP_041322341	1305	2
			XM_041466408	5516	**XP_041322342**	1304	
*Onychostruthus taczanowskii*	*Passeridae*	White-rumped snowfinch	XM_041408615	5583	**XP_041264549**	1305	2
*Passer montanus*	*Passeridae*	Eurasian tree sparrow	XM_039711203	5521	**XP_039567137**	1305	2
*Passer domesticus*	*Passeridae*	House sparrow	XM_064409095	5091	**XP_064265165**	1305	2
			XM_064409099	3423	XP_064265169	924	
*Ficedula albicollis*	*Muscicapidae*	Collared flycatcher	XM_016296384	5446	**XP_016151870**	1305	2
*Ammospiza caudacuta*	*Passerellidae*	Saltmarsh sparrow	XM_058814951	5468	**XP_058670934**	1309	2
			XM_058814958	5363	XP_058670941	1274	
*Ammospiza nelsoni*	*Passerellidae*	Nelson's sparrow	XM_059467201	5410	**XP_059323184**	1309	2
			XM_059467209	5305	XP_059323192	1274	
*Melospiza georgiana*	*Passerellidae*	Swamp sparrow	XM_058020327	5378	**XP_057876310**	1309	2
*Melospiza melodia melodia*	*Passerellidae*	Song sparrow	XM_063150769	5529	**XP_063006839**	1309	2
*Vidua macroura*	*Viduidae*	Pin-tailed whydah	XM_053982611	4277	XP_053838586	1007	1
*Vidua chalybeata*	*Viduidae*	Village indigobird	XM_053952119	5001	**XP_053808094**	1294	1
*Anomalospiza imberbis*	*Viduidae*	Cuckoo-finch	XM_068211947	4291	**XP_068068048**	1293	1
			XM_068211917	4615	XP_068068018	1293	
			XM_068211928	4621	XP_068068029	1293	
			XM_068211938	4800	XP_068068039	1293	
			XM_068211907	4683	XP_068068008	1293	
			XM_068211956	2779	XP_068068057	852	
*Taeniopygia guttata*	*Estrildidae*	Sunda zebra finch	XM_004186218	6091	**XP_004186266**	1295	1
			XM_072925499	3903	XP_072781600	722	
			XM_030264624	6279	XP_030120484	1295	
			XM_030264625	4536	XP_030120485	914	
			XM_030264623	6040	XP_030120483	1295	
*Lonchura striata*	*Estrildidae*	White-rumped munia	XM_021551649	5109	**XP_021407324**	1295	1
			XM_021551650	2964	XP_021407325	914	
*Melozone crissalis*	*Passerellidae*	California towhee	XM_054273719	4929	**XP_054129694**	1299	1
*Zonotrichia leucophrys gambelii*	*Passerellidae*	Gambel's white-crowned sparrow	XM_064704225	5390	**XP_064560295**	1302	1
			XM_064704226	5328	XP_064560296	1302	
*Zonotrichia albicollis*	*Passerellidae*	White-throated sparrow	XM_074535471	5473	**XP_074391572**	1302	1
			XM_026795583	5537	XP_026651384	1302	
			XM_014269074	5421	XP_014124549	1302	
*Oenanthe melanoleuca*	*Muscicapidae*	Eastern black-eared Wheatear	XM_056483309	9191	**XP_056339284**	1295	1
			XM_056483308	8844	XP_056339283	1295	
			XM_056483311	2971	XP_056339286	914	
*Molothrus ater*	*Icteridae*	Brown-headed cowbird	XM_036406499	5500	**XP_036262392**	1299	1
*Molothrus aeneus*	*Icteridae*	Bronzed cowbird	XM_066552898	5293	**XP_066408995**	1299	1
*Agelaius phoeniceus*	*Icteridae*	Red-winged blackbird	XM_054628034	5531	**XP_054484009**	1299	1
*Agelaius tricolor*	*Icteridae*	Tricolored blackbird	XM_071428933	5508	**XP_071285034**	1299	1
*Geospiza fortis*	*Thraupidae*	Medium ground finch	XM_031066762	6107	**XP_030922622**	1299	1
*Camarhynchus parvulus*	*Thraupidae*	Small tree finch	XM_030970533	4926	**XP_030826393**	1299	1
*Cinclus cinclus*	*Cinclidae*	White-throated dipper	XM_062494675	3882	**XP_062350659**	1293	1
*Sturnus vulgaris*	*Sturnidae*	Common starling	XM_014875658	4986	**XP_014731144**	1295	1
*Catharus ustulatus*	*Turdidae*	Swainson's thrush	XM_033070805	5491	**XP_032926696**	1300	1
*Pseudopodoces humilis*	*Paridae*	Ground tit	XM_005518778	5450	**XP_005518835**	1284	0
*Poecile atricapillus*	*Paridae*	Black-capped chickadee	XM_058831920	3376	XP_058687903	904	0
			XM_058831919	5729	**XP_058687902**	1285	
*Parus major*	*Paridae*	Great tit	XM_015615665	3523	XP_015471151	904	0
			XM_015615657	5495	**XP_015471143**	1285	
*Cyanistes caeruleus (Parus caeruleus)*	*Paridae*	Eurasian blue tit	XM_023920275	4396	**XP_023776043**	1285	0
			XM_023920276	3410	XP_023776044	904	
*Hirundo rustica*	*Hirundinidae*	Barn swallow	XM_040058125	5658	XP_039914059	1294	0
			XM_058419908	5580	**XP_058275891**	1281	
			XM_058419904	5572	XP_058275887	1281	
			XM_040058116	5413	XP_039914050	1294	
			XM_040058152	5261	XP_039914086	1281	
*Prinia subflava*	*Cisticolidae*	Tawny-flanked prinia	XM_063412806	5557	**XP_063268876**	1286	0
*Aphelocoma coerulescens*	*Corvidae*	Florida scrub-jay	XM_069006753	5460	**XP_068862854**	1285	0
*Corvus moneduloides*	*Corvidae*	New caledonian crow	XM_032113101	7146	**XP_031968992**	1285	0
*Corvus cornix cornix*	*Corvidae*	Hooded crow	XM_010399216	5516	**XP_010397518**	1285	0
*Corvus brachyrhynchos*	*Corvidae*	American crow	XM_008629889	7145	**XP_008628111**	1285	0
*Corvus kubaryi*	*Corvidae*	Mariana crow	XM_042043762	5504	**XP_041899696**	1285	0
*Corvus hawaiiensis*	*Corvidae*	Hawaiian crow	XM_048306620	5515	**XP_048162577**	1285	0
*Sylvia atricapilla*	*Sylviidae*	Eurasian blackcap	XM_066316830	3952	**XP_066172927**	1297	0
*Malurus melanocephalus*	*Maluridae*	Red-backed fairywren	XM_057383118	5503	**XP_057239101**	1284	0
*Pipra filicauda*	*Pipridae*	Wire-tailed manakin	XM_039379659	5655	**XP_039235593**	1285	0
			XM_039379665	5291	XP_039235599	1285	
			XM_039379655	5830	XP_039235589	1285	
*Neopelma chrysocephalum*	*Pipridae*	Saffron-crested tyrant-manakin	XM_027708361	5186	**XP_027564162**	1286	0
*Manacus candei*	*Pipridae*	White-collared manakin	XM_051802540	5035	**XP_051658500**	1285	0
			XM_051802539	5439	XP_051658499	1285	
*Pseudopipra pipra*	*Pipridae*	White-crowned manakin	XM_064678593	5080	**XP_064534663**	1285	0
			XM_064678602	5471	XP_064534672	1285	

For all listed birds, species and family names are provided according to the IOC list (56). In addition, the phylogenetic relationship of the bold-typed *Passeriformes* ABCB1 sequences is shown in Figure 3, and multiple sequence alignment of these sequences is provided in the Supplementary material.

### Generation of the Δ1 and Δ2 mutants of the Eurasian bullfinch ABCB1

The pcDNA5/FRT/TO-TOPO expression vector containing the full-length wild-type Eurasian bullfinch ABCB1 cDNA sequence was used for inverse PCR deletion using back-to-back primers flanking the site of deletion to delete either one (Δ1) or both (Δ2) tandem repeat insertions using the primers listed in Table 3. PCR amplifications using the primers Δ1-mutant-F and Δ1-mutant-R, as well as Δ2-mutant-F and Δ2-mutant-R, omitted the respective insertion motifs by inverse PCR amplification of the entire vector sequence. For PCR amplification the Phusion Flash High Fidelity DNA-Polymerase (Thermo Fisher Scientific) was used and the amplification was carried out on a Peqlab Primus 96 advanced gradient PCR cycler under the following conditions: initial denaturation for 5 min at 98 °C; 30 cycles of denaturation for 30 s at 98 °C, annealing for 30 s at 63 °C (Δ2 reaction) or 69 °C (Δ1 reaction), and extension for 140 s at 72 °C; final elongation for 5 min at 72 °C; and a final hold at 4 °C. After amplification, PCR products were digested with *Dpn*I (Thermo Fisher Scientific) overnight at 37 °C to digest the template vector DNA. On the following day, purification of the PCR fragments was performed using the HiYield PCR Clean-up Kit (SLG). Afterwards, the PCR fragments were re-ligated by incubation with T4 DNA Ligase (Thermo Fisher Scientific) overnight at 16 °C. Closed vector DNA was then transformed into TOP10 chemically competent *Escherichia coli*, and plasmid preparation was performed with GeneJET Plasmid Miniprep Kit (Thermo Fisher Scientific). Mutations were verified by DNA sequencing (Microsynth Seqlab).

**Table 3:** Back-to-back primers used for inverse PCR deletion to generate the Δ1 and Δ2 mutants of the Eurasian bullfinch ABCB1 transporter mutants.

Primer	Sequence (5' → 3')	Primer T_m_
Δ1-mutant-F	aat gta ctt cca tca tca gaa aat tat gag aat gta cgt agt gtc aaa aac	71°C
Δ1-mutant-R	ctc aga att ttc tga tga tgg aac ttc agc ttc tat tgc c	72°C
Δ2-mutant-F	aat gta cgt agt gtc aaa aac agt g	63°C
Δ2-mutant-R	ctc aga att ttc tga tga tgg aac ttc	63°C

### Stably transfected HEK293 cell lines

The Flp-In T-REx Human Embryonic Kidney (HEK) 293 Cell Line (Invitrogen), further referred to as HEK293, was used for stable transfection of the wild-type and both mutant Eurasian bullfinch ABCB1 constructs via Flp-recombinase mediated FRT integration as reported (27). For expression of the gene of interest, cells were treated with 1 μg/mL tetracycline (TET, Carl Roth) for 72 h. Cells were maintained in Dulbecco's modified Eagle medium (DMEM) and F12 nutrient mixture 1:1 (Gibco, Carlsbad, CA, USA), supplemented with 10% fetal calf serum (Gibco), 4 mM L-glutamine (Gibco), 100 U/mL penicillin (Gibco), and 100 μg/mL streptomycin (Gibco) in an incubator at 5% CO_2_, 37 °C, and 95% humidity. All cell lines were routinely cultured in 100 mm Petri dishes (Sarstedt, Nümbrecht, Germany) and passaged when they reached 80–90% confluency. For stable transfection, cells were seeded on poly-D-lysine (Gibco) coated 6-well plates (Sarstedt) with 1,000,000 cells/well. The transfection procedure was performed at 70% confluence by using Lipofectamine 2000 Reagent (Invitrogen). The mixture was prepared in the Minimum Essential Medium Opti-MEM Reduced Serum Medium (Gibco). The pcDNA5/FRT/TO-TOPO vector containing the wild-type Eurasian bullfinch ABCB1 (double insertion) or the mutant (Δ1 and Δ2 deletions) constructs was co-transfected with the pOG44 Flp-Recombinase Expression Vector (Invitrogen). Transfected cells were selected by culturing in selective medium containing 100 μg/mL hygromycin B (Carl Roth) and 15 μg/mL blasticidin S HCl (Corning, Mediatech, NY, USA) for 7–10 days. The surviving cell clones were combined to generate polyclonal cell lines. HEK293 cell lines stably expressing the wild-type (WT) canine ABCB1 (dog WT) or the nt230(del4) mutant canine ABCB1 (dog KO) transporters were used as controls, as previously reported (14, 28, 30).

### Western blot analysis of Eurasian bullfinch ABCB1 protein expression

Each stably transfected cell line was seeded in three wells of a poly-D-lysine (Gibco) coated 6-well plate (Sarstedt) with 2,000,000 cells/well and induced with 1 μg/mL TET. After 72 h, cells were harvested and lysed in a buffer containing 150 mM NaCl, 50 mM Tris, 1% NP-40, 0.5% sodium deoxycholate, 0.1% sodium dodecyl sulfate (SDS), pH 7.4, supplemented with Halt Protease-Inhibitor-Cocktail (100x) (Thermo Fisher Scientific). Lysates were pooled per cell line, and the protein content was determined by using the BCA Protein Assay Kit (MilliporeSigma, Burlington, MA, USA). For each non-boiled sample, 75 mg of protein were prepared with 4x Laemmli buffer (250 mM Tris base, 8% SDS, 40% glycerol, 10% 2-mercaptoethanol, 0.2% bromophenol blue) and applied to the gel. Samples were separated on an 8% SDS polyacrylamide gel by gel electrophoresis (SDS-PAGE). Afterwards, proteins were blotted on polyvinylidene fluoride membranes (Carl Roth) for 1 h. Membranes were blocked with 5% milk solution for 1 h at room temperature (RT), incubated with the primary monoclonal mouse C219 anti-ABCB1 antibody (dilution 1:1000, Cat# 517310, Calbiochem, Billerica, MA, USA) overnight at 4 °C and then washed four times for 15 min with Tris-buffered saline with tween-20 (TBS-T buffer, containing 137 mM NaCl, 10 mM Tris base, 0.05% tween-20, pH 8.0). The same washing procedure was performed after incubation with the secondary horseradish peroxidase-conjugated polyclonal rabbit anti-mouse antibody (1:5,000 dilution, Cat# A9044, Sigma-Aldrich) for 1 h at RT. Blots were imaged in chemiluminescent solution containing Super Signal West Pico Plus Chemiluminescent Substrate (Thermo Fisher Scientific) on a ChemiDoc imaging system (Bio-Rad, Hercules, CA, USA). For loading control, β-actin was detected. Therefore, the membranes were stripped using mild stripping buffer (0.2 M glycine, 0.1% SDS, 1% Tween-20, pH 2.2) for 30 min, blocked, and incubated as described above. As a primary antibody, the monoclonal mouse anti-β-actin antibody (1:5,000 dilution, Cat# A5441, Sigma-Aldrich) was used. The blots were quantitatively evaluated for the adjusted volume of each band using Image Lab 6.1 (Bio-Rad).

### Transport experiments with Rhodamine 123 (Rh123)

The transport experiments were performed following a recently validated and published assay (30). Briefly, the stably transfected cell lines were seeded on poly-D-lysine-coated 24-well plates (Sarstedt) in the same passage as used for Western blot analysis with 450,000 cells/well. To induce ABCB1 expression, half of the cells were treated with 1 μg/mL TET (+TET). The other half was used as a non-induced control (-TET). Cells were grown to confluence over 72 h and washed with 37 °C tempered phosphate-buffered saline (PBS, 137 mM NaCl, 2.7 mM KCl, 1.5 mM KH_2_PO_4_, 7.3 mM Na_2_HPO_4_, pH 7.4). Subsequently, preincubation with or without the inhibitor TQR in a concentration of 1 μM was performed for 30 min at RT. Therefore, DMEM without phenol red (Gibco) was used as transport medium, equalized with DMSO (Carl Roth) concentration between the aliquots with or without TQR. The maximum DMSO concentration was 0.3%. Afterwards, the ABCB1 substrate Rh123 was added to a final concentration of 5 μM, and incubation lasted for 90 min at RT (uptake phase). The uptake was stopped and the substrate removed by washing twice with ice-cold PBS. Subsequently, ABCB1-mediated Rh123 efflux was allowed for 60 min at 37 °C in transport media with or without TQR (efflux phase). The transport experiments were stopped by washing twice with ice-cold PBS, and the cells were lysed for 90 min at 37 °C by using 1 N NaOH with 0.1% SDS. From the cell lysates, 3 × 100 μl aliquots were used per well for fluorescence measurement at excitation of 490 nm and emission of 510-570 nm in a GloMax-Multi+ Detection System (Promega, Madison, WI, USA) to quantify Rh123 as reported before (30). Mean values were calculated from these triplicates and scaled to a volume of 1 mL. The protein concentration was determined with standard dilutions of bovine serum albumin as reported (31). Finally, the relative fluorescence units (RFU) were normalized to the protein amount and expressed as a ratio RFU/μg protein.

### Inhibition of the ABCB1-mediated Rh123 efflux with TQR and IVM and determination of half-maximal inhibitory concentrations (IC_50_)

Inhibition of the Eurasian bullfinch ABCB1-mediated Rh123 efflux was done with TQR and IVM as reported previously (30). As a reference control, HEK293 cells stably expressing the WT canine ABCB1 efflux transporter (here referred to as dog WT) were used as reported previously (30). All cells were seeded on 96-well plates (Sarstedt) with 90,000 cells/well, and ABCB1 overexpression was induced with TET treatment at 1 μg/mL. Then, the transport of Rh123 was analyzed in the presence of increasing concentrations of TQR (at 1, 3, 10, 30, 100, 300 nM, and 1 μM concentrations) or IVM (at 0.1, 0.3, 1, 3, 10, and 30 μM concentrations). The uptake and the efflux phases were performed as described above. This time, the efflux phase was stopped by washing twice with ice-cold PBS, and the Rh123 fluorescence was directly measured in the remaining cells without cell lysis. The Rh123 efflux in the presence of 1 μM TQR served as 0% efflux control, while the Rh123 efflux in the absence of any inhibitor was set to 100%. Determination of IC_50_ values was done by non-linear regression analysis with equation log (inhibitor) vs. response—variable slope settings using GraphPad Prism 6 (GraphPad Software Inc., San Diego, CA, USA).

### General data evaluation and statistical analysis

All graphs were generated by GraphPad Prism 6. Statistical analysis was performed by one-way or two-way ANOVA with *p* < 0.05 and Bonferroni's multiple comparison test as indicated in the figure legends.

## Results

### Cloning and sequence analysis of the full-length Eurasian bullfinch ABCB1 transporter

**Figure 1:**
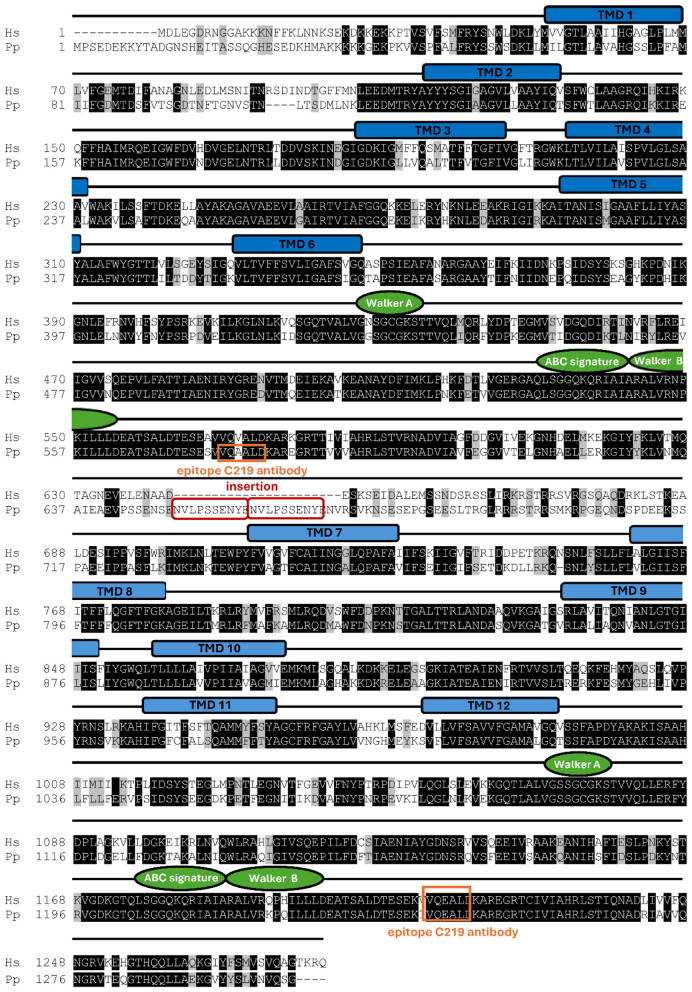
Protein alignment of the Eurasian bullfinch and human ABCB1 transporter protein sequences. The full-length ABCB1 sequence of the Eurasian bullfinch (referred to as Pp) was cloned for the first time in the present study and was deposited into the GenBank database with accession numbers HG917089 for the cDNA and CDM63410 for the protein sequence. The human ABCB1 protein sequence (referred to as Hs) was derived from GenBank accession number NP_001335873. Sequence alignment was performed with ClustalW with the software DNASTAR MegAlign Pro 16 (Lasergene, Madison, WI, USA). Black shadings indicate identical sequences; gray shadings indicate similarities. Typical structural motifs of ABC transporters are indicated in green, and the localization of the transmembrane domains of the human ABCB1 protein is depicted as blue bars. The epitope of the ABCB1 primary C219 antibody, used for Western blotting (see Figure 4), is marked with an orange box, and the tandem repeat insertion of the Eurasian bullfinch ABCB1 sequence is marked with a red box.

To clone the full-length ORF cDNA of the Eurasian bullfinch ABCB1 transporter, we used primers derived from the chicken ABCB1 cDNA (GenBank accession number NM_204894.2) to PCR amplify and sequence four overlapping segments of the ABCB1 ORF (see Table 1). The 5′- and 3′-untranslated regions upstream of the start codon and downstream of the stop codon were amplified by use of RACE-PCR (see further details in the Materials and Methods section), and these sequences were used to generate Eurasian bullfinch-specific primers that finally allowed amplification of the full ABCB1 ORF cDNA. The Eurasian bullfinch ABCB1 ORF consists of 3915 base pairs (bp) that code for the 1304-amino-acid (aa) ABCB1 transporter protein. Figure 1 shows a sequence alignment between the Eurasian bullfinch ABCB1 (Pp) and the human ABCB1 (Hs) transporters. Identical aa are shaded black, and similar aa are highlighted with gray shading. The Eurasian bullfinch ABCB1 transporter protein is slightly longer than the human ABCB1 transporter protein of 1280 aa (GenBank accession number NP_001335873). More detailed sequence analysis revealed highly conserved regions covering the transmembrane domain (TMD) part of the protein and the nucleotide binding domains (NBDs) with their ABC signature, Walker A and Walker B motifs. In contrast, a highly variable region was found in the intracellular linker region, which connects the two halves of the transporter (32). The most striking difference between the two proteins is a tandem repeat insertion of the 10-aa peptide NVLPSSENYE, which is completely absent in the human ABCB1 sequence, and is located within the intracellular linker region.

### Systematic analysis for the passerine ABCB1 insertion motif

**Figure 2:**
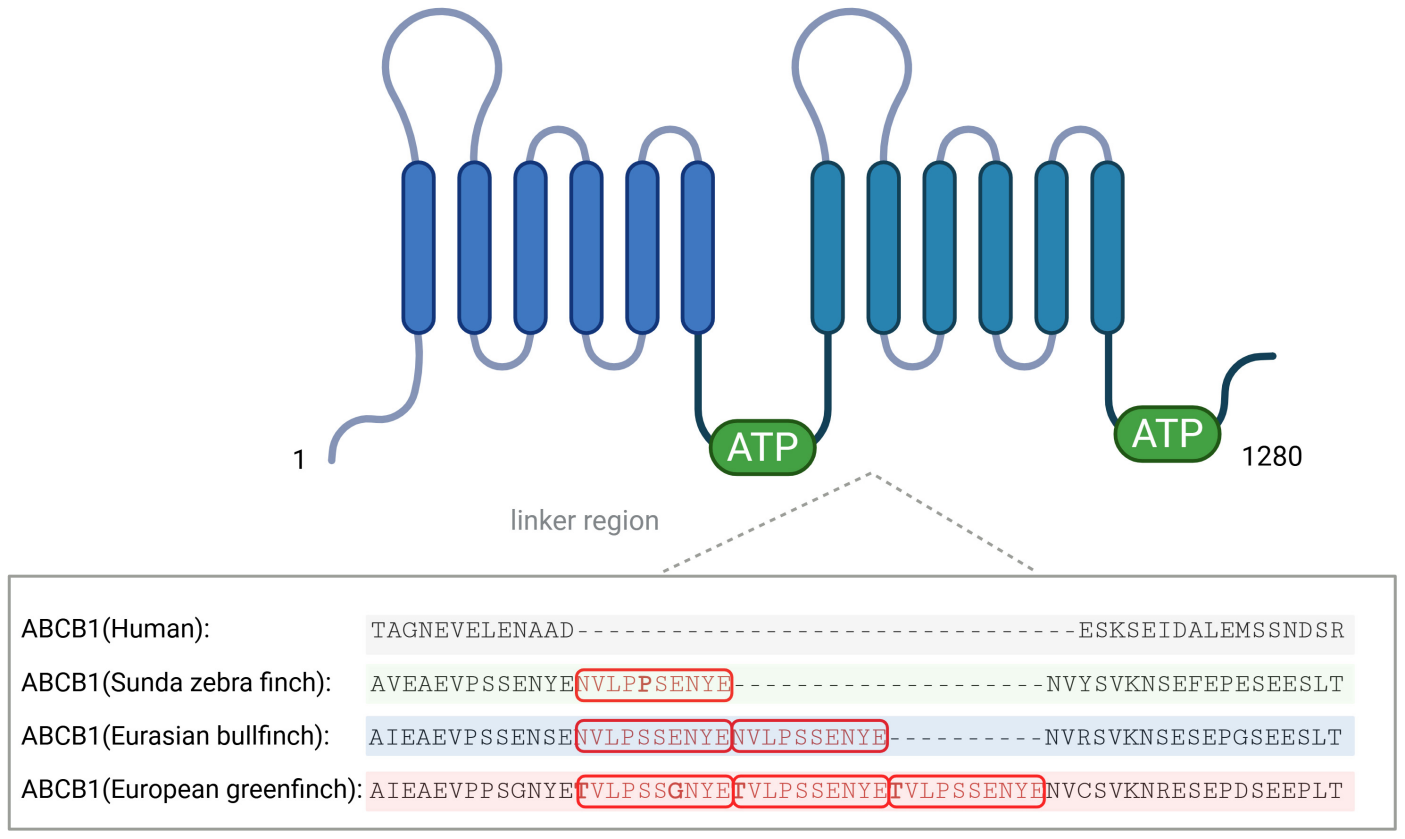
Presence of the insertion variants in representative *Passeriformes* species. Excerpt of the ABCB1 protein alignment at the site of sequence insertion of three representative *Passeriformes* species. The insertion of the 10-aa motif with the consensus sequence “NVLPSSENYE” is present as single insertion (e.g., in the Sunda zebra finch, highlighted by green shading), tandem repeat insertion (e.g., in the Eurasian bullfinch, highlighted by blue shading), or triple repeat insertion (e.g., in the European greenfinch, highlighted by red shading) in different *Passeriformes* species. The insertion occurs at position 650 of the Eurasian bullfinch ABCB1 protein. Variations from the consensus insertion sequence are indicated in bold. The human ABCB1 sequence serves as a control and has no insertion at this site. A full sequence alignment of all ABCB1 sequences from *Passeriformes* species listed in Table 2 is provided in the Supplementary material. Created with BioRender.com.

Next, we analyzed whether this tandem repeat insertion of the Eurasian bullfinch ABCB1 is also present in the ABCB1 transporters of other *Passeriformes* species. Systematic BLAST search using the NCBI blastn program with the cDNA sequence of the Eurasian bullfinch ABCB1 (GenBank accession number HG917089) as the query revealed 86 additional sequences from 51 related *Passeriformes* species that are all listed in Table 2. For some of these passerine ABCB1 sequences, multiple GenBank entries were identified. Closer analysis of the variable linker region of all these *Passeriformes* ABCB1 sequences revealed four distinct variants: a single-motif insertion, a tandem repeat insertion, a triple repeat insertion, or complete absence of the insertion (Table 2). These insertions are highlighted in a protein alignment of representative ABCB1 transporter proteins from three different *Passeriformes* species, namely Sunda zebra finch with a single-motif insertion, Eurasian bullfinch with a tandem repeat insertion, and European greenfinch with a triple repeat insertion (Figure 2). For comparison, the human ABCB1 transporter protein was included in the alignment and does not contain any insertions in the linker region. A full sequence alignment of all sequences listed in Table 2 is provided in the Supplementary material.

Apart from the Eurasian bullfinch, a tandem repeat insertion was detected in 14 *Passeriformes* species of five different families: European serin (*Serinus serinus*, Linnaeus, 1766), Atlantic canary (*Serinus canaria*, Linnaeus, 1758), Eurasian siskin (*Spinus spinus*, Linnaeus, 1758), and house finch (*Haemorhous mexicanus*, Müller, PLS, 1776) of the *Fringillidae* family; white wagtail (*Motacilla alba*, Linnaeus, 1758) of the *Motacillidae* family; rufous-necked snowfinch (*Pyrgilauda ruficollis*, Blanford, 1871), white-rumped snowfinch (*Onychostruthus taczanowskii*, Przevalski, 1876), Eurasian tree sparrow (*Passer montanus*, Linnaeus, 1758), and house sparrow (*Passer domesticus*, Linnaeus, 1758) from the *Passeridae* family; collared flycatcher (*Ficedula albicollis*, Temminck, 1815) from the *Muscicapidae* family; saltmarsh sparrow (*Ammospiza caudacuta*, Gmelin, JF, 1788), Nelson's sparrow (*Ammospiza nelsoni*, Allen, JA, 1875), swamp sparrow (*Melospiza georgiana*, Latham, 1790), and song sparrow (*Melospiza melodia melodia*, Wilson, A, 1810) from the *Passerellidae* family (Table 2). A single insertion of the 10-aa peptide was found in a total of 18 *Passeriformes* species from 9 different families, and a triple insertion with some minor sequence variations within the repeating insertion motifs was only detected in the European greenfinch of the *Fringillidae* family (Table 2). Of note, even an intensive BLAST search with the European greenfinch ABCB1 cDNA as the query against all available genomic data revealed no additional bird species with a triple repeat insertion in the ABCB1 linker region, making the European greenfinch the only currently known representative of this particular genetic variant. In contrast to these *Passeriformes* species with insertions in the ABCB1 linker region, a complete absence of insertion was detected for 18 *Passeriformes* species from 7 different families (Table 2).

**Figure 3:**
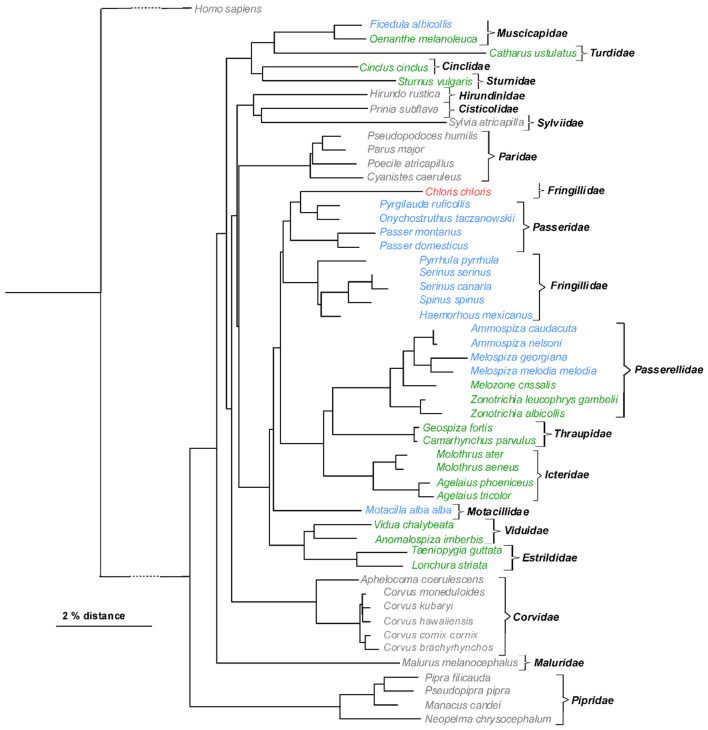
Phylogenetic relationship of ABCB1 sequences of *Passeriformes* species. A phylogenetic tree was generated based on all ABCB1 protein sequences from *Passeriformes* species listed in Table 2. The resulting tree was visualized with TreeViewer, whereby the coloring represents the number of insertions: no insertion = gray; single insertion = green; tandem repeat insertion = blue; triple repeat insertion = red. For comparison, human ABCB1 (*Homo sapiens*, GenBank accession number NP_001335873) was included.

Finally, it was analyzed whether there is any correlation between the phylogenetic relationship among the *Passeriformes* species and the occurrence of the ABCB1 linker region insertion variations (Figure 3). For most of the *Passeriformes* families, there was a unique number of repeats, and families with the identical insertion variant generally clustered. However, there were three exceptions: (I) the European greenfinch was the only member of the *Fringillidae* family with a triple repeat insertion, whereas all other members of this family (the Eurasian bullfinch, house finch, Atlantic canary, European serin, and Eurasian siskin) all showed a tandem repeat insertion; (II) within the *Muscicapidae* family, the collared flycatcher revealed a tandem repeat insertion, whereas the Eastern black-eared wheatear (*Oenanthe melanoleuca*, Güldenstädt, 1775) had a single insertion motif; and (III) within the *Passerellidae* family, saltmarsh sparrow, Nelson's sparrow, swamp sparrow, and song sparrow showed a tandem repeat insertion, whereas California towhee (*Melozone crissalis*, Vigors, 1839), Gambel's white-crowned sparrow (*Zonotrichia leucophrys gambelii*, Nuttall, 1840), and white-throated sparrow (*Zonotrichia albicollis*, Gmelin, JF, 1789) revealed a single insertion motif. Based on this, it can be concluded that the insertion variants show a predominant, though not exclusive, association with specific *Passeriformes* families.

### Protein expression and efflux function of the Eurasian bullfinch ABCB1 transporter

**Figure 4:**
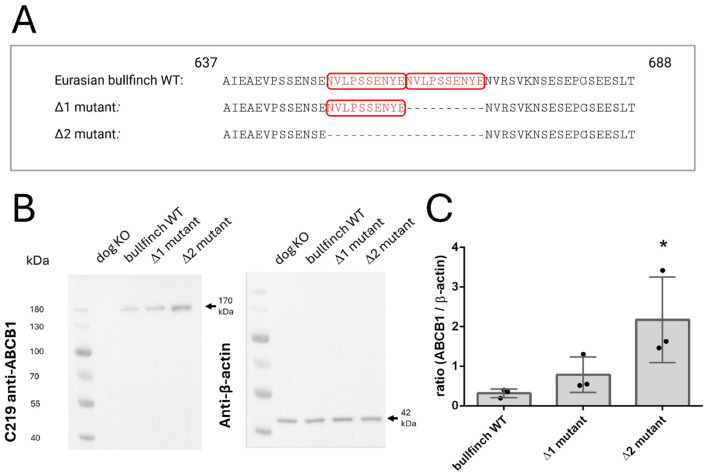
Detection of the wild-type (WT) and Δ1/Δ2 mutant Eurasian bullfinch ABCB1 transporter proteins by Western blotting. **(A)** Alignment of the tandem repeat insertion of the ABCB1 sequence of the Eurasian bullfinch and the Δ1 and Δ2 mutant constructs. The insertion peptide is marked with a red box. **(B)** Representative Western blot analysis of the Eurasian bullfinch ABCB1 transporter proteins (WT, Δ1 mutant, and Δ2 mutant) stably expressed in transfected HEK293 cells. Detection of β-actin expression served as the loading control. HEK293 cells stably transfected with the ABCB1 cDNA construct of an ABCB1 mutant dog lacking any ABCB1 protein expression [see (30)] were included as a negative control for Western blotting. The ABCB1 transporter protein of the Eurasian bullfinch revealed an apparent molecular weight of ~170 kDa, while β-actin was detected at ~42 kDa. **(C)** Relative expression of the WT (tandem insertion) and the Δ1 and Δ2 mutant ABCB1 transporter proteins of the Eurasian bullfinch in relation to β-actin. Expression levels of ABCB1 and β-actin were analyzed in three independent Western blot experiments; one representative of them is shown in **(B)**. A scan of all three Western blots and the relative adjusted band volumes is provided in the Supplementary material. *Significantly higher ABCB1 protein expression after full deletion of the tandem insertion (Δ2) compared to the WT Eurasian bullfinch ABCB1 with the tandem repeat insertion, following one-way ANOVA with *p* < 0.05. Figure 4A was created with BioRender.com.

**Figure 5:**
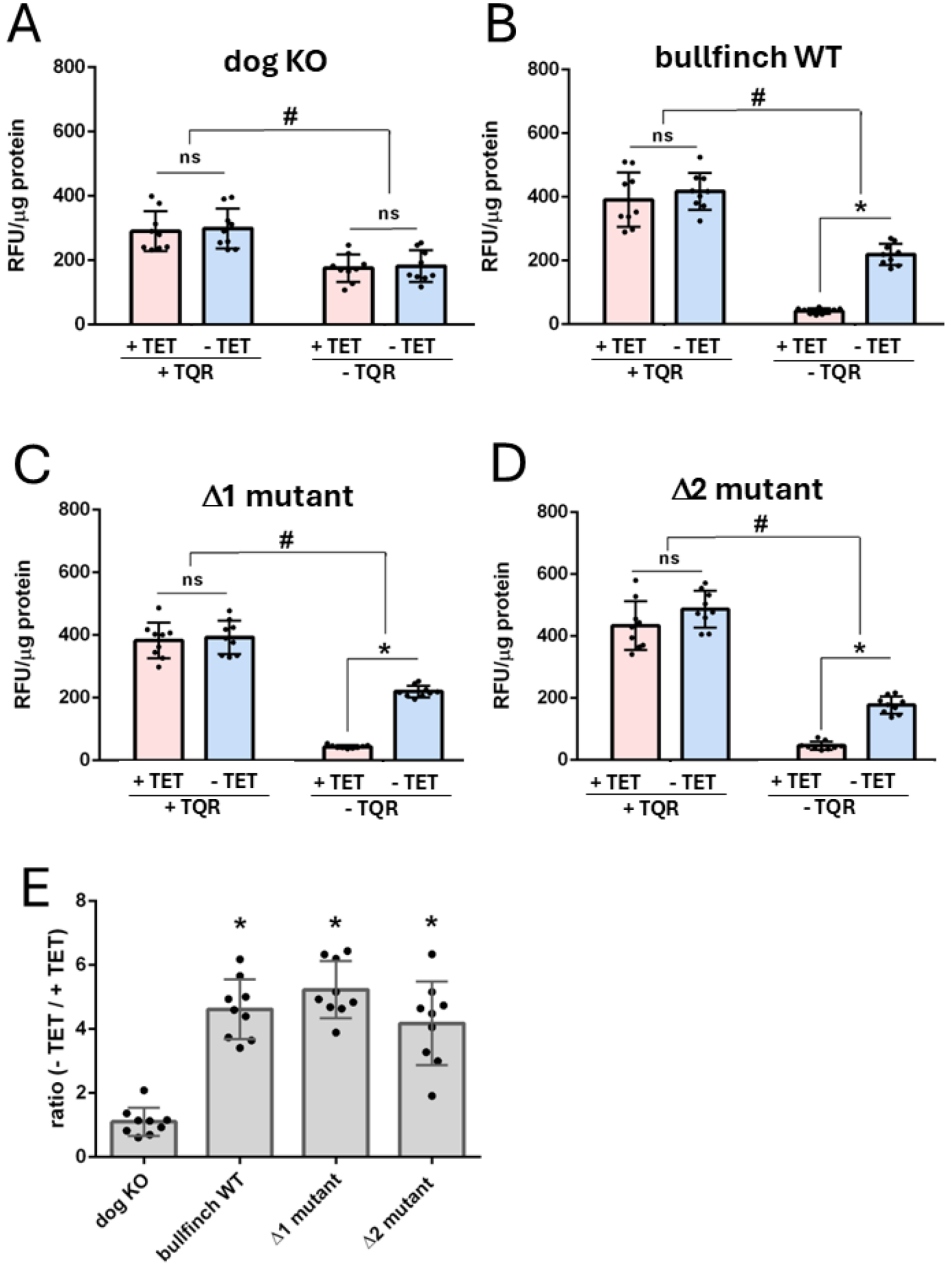
Characterization of the Rh123 efflux activity of the Eurasian bullfinch ABCB1 WT and Δ1/Δ2 mutant transporter proteins. Efflux experiments were performed with Rh123 as the ABCB1 transporter probe substrate, and after an efflux phase, the remaining cell-based Rh123 was quantified in a fluorescence reader. The data are provided on the y-axis as relative fluorescence units (RFU) that were normalized to the protein content of the cells (RFU/μg protein). Data represent mean ± SD from three independent experiments, each with triplicate determination (*n* = 9). The following ABCB1-transfected HEK293 cell lines were analyzed: **(A)** HEK293 cells stably transfected with the ABCB1 cDNA constructs of an ABCB1 mutant dog lacking any ABCB1 protein expression [see (30)] serving as a negative control; HEK293 cells stably overexpressing the WT **(B)**, Δ1 mutant **(C)**, or Δ2 mutant **(D)** of the Eurasian bullfinch ABCB1 transporter protein. In the transfected HEK293 cells, ABCB1 expression is under the control of a TET-regulated promoter. After TET treatment (+TET) the cells start to express the ABCB1 protein and are enabled to efflux Rh123. In the absence of the ABCB1 inhibitor TQR, the difference between +TET and −TET reflects the ABCB1 efflux activity for the Rh123 efflux. *Significant Rh123 efflux according to two-way ANOVA with *p* < 0.05. As HEK293 cells also show endogenous expression of human ABCB1, cells were additionally treated with the ABCB1 inhibitor TQR to block any ABCB1-mediated Rh123 efflux activity. In this situation, TET-treated (+TET) and TET-untreated (−TET) cells showed an identical level of Rh123 accumulation. ^#^Significantly higher Rh123 accumulation in the presence of TQR compared to cells without TQR treatment, according to two-way ANOVA with *p* < 0.05.

Finally, we assessed the impact of this tandem repeat insertion on the Eurasian bullfinch ABCB1 transporter efflux activity. This analysis aimed to determine whether the insertion in the ABCB1 linker region—either as a single motif or as a tandem repeat—affects ABCB1 protein expression and transporter efflux function. Therefore, three different variants of the Eurasian bullfinch ABCB1 transporter protein were generated and analyzed: the WT sequence with the tandem repeat insertion, a Δ1 mutant with only one remaining insertion motif, and a Δ2 mutant lacking the entire tandem repeat insertion motif (Figure 4A). All three constructs were stably transfected into HEK293 cells and used for Western blot detection of the ABCB1 protein expression (Figure 4B), as well as for functional efflux assays with the prototypic fluorescence ABCB1 substrate Rh123 (Figure 5). It is well known that HEK293 cells have low basal expression of the endogenous human ABCB1 protein (30). To appropriately consider this point, we included an additional control by using HEK293 cells that were stably transfected with the canine nt230(del4) mutant ABCB1 construct (dog KO) and that lack any recombinant ABCB1 protein expression as previously shown (14, 30). For Western blot analysis, the C219 anti-ABCB1 antibody was used, which reacts with a short epitope (VQEALD or VQAALD) of the ABCB1 protein (33). This epitope is not only present in the human ABCB1 protein but is also conserved in the Eurasian bullfinch ABCB1 protein (Figure 1). As shown in Figure 4B, expression of the ABCB1 transporter protein could clearly be demonstrated for all three variants of the Eurasian bullfinch ABCB1 protein, namely, the WT, the Δ1 mutant, and the Δ2 mutant. The Eurasian bullfinch ABCB1 protein revealed an apparent molecular weight of ~170 kDa. All blots were additionally probed with an anti-β-actin antibody as a loading control. The Western blot was repeated in three independent experiments, the band intensities were analyzed for each blot, and the anti-ABCB1 band intensities were then related to the band intensities of the anti-β-actin signals (see Supplementary material). As shown in Figure 4C, ABCB1 protein expression in the HEK293 cell lines stably transfected with the Δ2 mutant was significantly higher compared to the Eurasian bullfinch WT and Δ1 mutant ABCB1 proteins, indicating that the tandem insertion motif in the Eurasian bullfinch WT ABCB1 reduced total ABCB1 protein expression in the HEK293 cells.

### Efflux activity of the Eurasian bullfinch ABCB1 transporter protein

In the next step we analyzed the efflux activity of all three European bullfinch ABCB1 constructs (Figure 5). In this assay, all cell lines were preloaded with 5 μM Rh123 in the uptake phase and after an efflux phase the remaining cellular fluorescence was measured. As already mentioned above, HEK293 cells show a low basal expression of the endogenous human ABCB1 transporter protein (30). To appropriately consider this effect in our experimental approaches, we used three different controls for the Rh123 efflux assay: (1) the use of HEK293 cells stably expressing the canine nt230(del4) mutant ABCB1 construct (dog KO) that has no transport activity as reported before (30) (Figure 5A); (2) TET pretreated (+TET) vs. non-TET induced (−TET) cells; and (3) measurements in the presence (+TQR) or absence (−TQR) of the ABCB1 inhibitor TQR (Figures 5A–D).

In detail, for all cell lines, Rh123 efflux was analyzed in cells pretreated with TET (+TET) to induce overexpression of the respective recombinant Eurasian bullfinch ABCB1 variant. In this approach, low Rh123 fluorescence is expected when the recombinant ABCB1 protein is functionally active. These values can be directly compared with cells not treated with TET (−TET). These cells still express the endogenous human ABCB1 efflux carrier and some minor recombinant ABCB1 protein from the slightly leaky TET promoter (27, 34) but show higher Rh123 fluorescence compared to the +TET control when the recombinant ABCB1 proteins are transporting Rh123. To get an estimate about the unspecific background ABCB1 expression, all cell lines were additionally analyzed in the presence of TQR, which completely blocks any ABCB1-mediated Rh123 efflux and, therefore, high Rh123 accumulation and fluorescence is expected. As shown in Figures 5B–D, overexpression of the WT or Δ1/Δ2 mutant Eurasian bullfinch ABCB1 transporter proteins significantly reduced the cell-associated Rh123 fluorescence compared to the -TET control, clearly demonstrating their role as functional efflux carriers. In contrast, HEK293 cells stably expressing the canine nt230(del4) mutant ABCB1 construct (dog KO) revealed no ABCB1-mediated Rh123 efflux at all (Figure 5A). TQR completely blocked the ABCB1 activity in all cell lines, resulting in significantly higher Rh123 accumulation and fluorescence compared to any condition in the absence of TQR. Under TQR incubation, there was no difference between the +TET and -TET treated cells, demonstrating complete block of the ABCB1 transporter proteins at the used TQR inhibitor concentration. All transport assays were done in three independent experiments, each with triplicate determinations (*n* = 9). Although a quantitative comparison of transporter kinetics was not possible because only total ABCB1 expression in HEK293 cells was assessed by Western blotting, and not the number of functional transporters at the plasma membrane, relative transport rates could be compared using the Rh123 fluorescence ratio between the -TET and +TET conditions. Using this approach, the Eurasian bullfinch WT and Δ1/Δ2 mutant ABCB1 variants exhibited comparable relative transport rates that were significantly higher compared to the canine mutant ABCB1 construct, showing a transport ratio of “1” and, thereby, a complete loss-of-function. Taken together, these results clearly demonstrate functional transporter expression and efflux activity of the Eurasian bullfinch WT ABCB1 transporter and its Δ1 and Δ2 mutant variants.

### Inhibition of ABCB1-mediated Rh123 efflux by IVM and TQR

**Figure 6:**
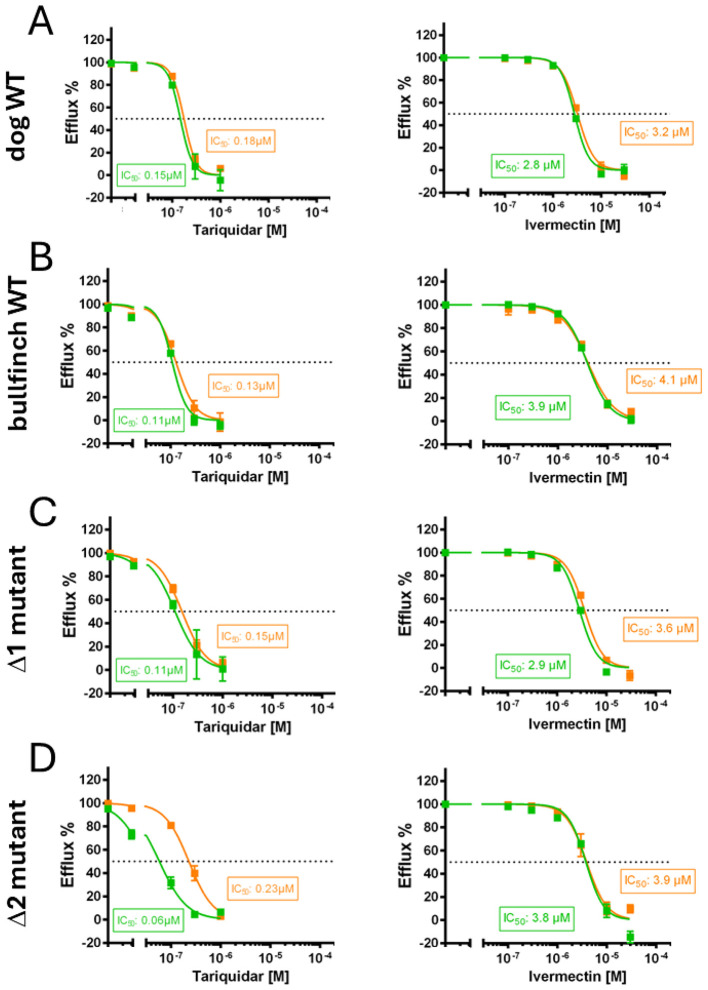
Inhibition of the ABCB1-mediated Rh123 efflux activity by the ABCB1 inhibitors tariquidar (TQR) and ivermectin (IVM), and determination of IC_50_ values. **(A)** For assay control, Rh123 efflux was analyzed for the canine wild-type ABCB transporter protein (dog WT). Rh123 efflux was also measured for the Eurasian bullfinch **(B)** wild-type (WT) and the **(C)** Δ1 and **(D)** Δ2 mutant ABCB1 transporter proteins. In all cell lines, Rh123 efflux was analyzed in the absence and presence of increasing concentrations of either TQR (left panels) or IVM (right panels). Cell-associated fluorescence in the absence of any inhibitor was set to 100% and Rh123 fluorescence in the presence of 1 μM TQR was set to 0%. Then, IC_50_ values were calculated by non-linear regression analysis. Two independent experiments are shown, each with quadruplicate determinations. Data represent means ± SD. IC_50_ values are provided in the figure. The corresponding 95% confidence intervals for all IC_50_ data are provided in the Supplementary material.

Finally, it was assessed whether the ABCB1-mediated Rh123 efflux of the Eurasian bullfinch WT and its Δ1 and Δ2 mutant variants can be inhibited with the established ABCB1 inhibitors TQR and IVM. For these experiments, ABCB1-mediated Rh123 efflux was analyzed at increasing concentrations of TQR (Figure 6, left) or IVM (Figure 6, right) to calculate IC_50_ values. For these experiments, HEK293 cells stably expressing the wild-type dog ABCB1 transporter (dog WT) were added as a control (Figure 6A). In these dog WT cells, IC_50_ values were previously determined to 3.6 and 4.2 μM for IVM, as well as 0.06 and 0.12 μM for TQR, in two independent experiments (30). All IC_50_ experiments in the present study were also performed in duplicate, and the IC_50_ values from all experiments are given in Figure 6. In addition, the 95% confidence intervals for all IC_50_ values are provided in the Supplementary material. The following IC_50_ values were determined for TQR: IC_50_ = 0.15 and 0.18 μM for dog WT ABCB1 (Figure 6A), IC_50_ = 0.11 and 0.13 μM for Eurasian bullfinch WT ABCB1 (Figure 6B), IC_50_ = 0.11 and 0.15 μM for the Δ1 mutant (Figure 6C), as well as 0.06 and 0.23 μM for the Δ2 mutant (Figure 6D). For the antiparasitic drug IVM, the following IC_50_ values were determined: IC_50_ = 2.8 and 3.2 μM for dog WT ABCB1 (Figure 6A), IC_50_ = 3.9 and 4.1 μM for Eurasian bullfinch WT ABCB1 (Figure 6B), IC_50_ = 2.9 and 3.6 μM for the Δ1 mutant (Figure 6C), as well as 3.8 and 3.9 μM for the Δ2 mutant (Figure 6D). Overall, these experiments indicate comparable inhibitory potencies of TQR and IVM between the WT and the Δ1/Δ2 mutant Eurasian bullfinch ABCB1 variants and with the WT dog ABCB1 transporter.

## Discussion

### Suspected IVM sensitivity in passerine birds

In dogs and cats, IVM sensitivity is strongly associated with a deletion mutation in the *ABCB1* gene, which abolishes ABCB1 expression at the BBB, impairs drug efflux, and finally enhances drug brain penetration (6, 10, 21, 22). On this basis, we hypothesized that the suspected IVM sensitivity in some passerine birds, including the Eurasian bullfinch (23–26), might have a genetic origin. To test this, the full-length ABCB1 sequence of the Eurasian bullfinch was cloned, sequenced, and stably transfected into HEK293 cells. Western blot analysis confirmed robust ABCB1 expression for all variants. The ABCB1-mediated efflux rates of the substrate Rh123 and inhibition of the Rh123 efflux with the inhibitors TQR and IVM were comparable for all three Eurasian bullfinch ABCB1 variants and were, in addition, comparable with the canine ABCB1 transporter protein. Based on this, we can exclude the presence of a gene deletion mutation in the Eurasian bullfinch ABCB1 comparable to that described in dogs and cats.

Reports of IVM sensitivity in passerine birds describe neurological toxicity in the European goldfinch and the Eurasian bullfinch at doses of 0.3–0.4 mg/kg (23). However, other birds have been reported to tolerate IVM treatment: budgerigars at 20 mg/kg (23); Atlantic canaries at 0.4 mg/kg (23); Sudan zebra finches at 0.2 mg/kg (35); and *Falconiformes* at 5 mg/kg (25). These observations indicate that birds are not inherently sensitive to IVM. Of note, the Atlantic canary, together with the Eurasian bullfinch, showed a tandem repeat insertion in the ABCB1 linker region, excluding this sequence insertion as the dominant genetic factor for IVM sensitivity. Moreover, the present study clearly demonstrates that Eurasian bullfinch ABCB1 is functionally intact, with efflux activity comparable to canine ABCB1, additionally arguing against a genetically mediated transporter defect as the cause of IVM sensitivity in this species. Against this background, individual cases of apparent sensitivity may reflect extrinsic factors, including comorbidities or concomitant medications that alter IVM brain penetration, as well as challenges in achieving accurate IVM dosing with formulations intended for larger animals. Such factors might contribute to the reported neurological signs in the European goldfinch and the Eurasian bullfinch (23).

Another noteworthy aspect is that even among ABCB1-deficient individuals, substantial interspecies variation in tolerated IVM doses has been reported, suggesting differences in central nervous system receptor susceptibility. For example, neurological toxicity in ABCB1-deficient CF-1 mice (PGPmut) was observed at oral IVM doses ≥ 0.35 mg/kg, as assessed by impaired rotarod performance (36). In contrast, ABCB1-deficient dogs developed neurological toxicity at IVM doses as low as ≥ 0.1 mg/kg (1, 37). Furthermore, although brain penetration of IVM is not increased in knockout mice lacking the ABC transporter breast cancer resistance protein (BCRP, ABCG2) (20), this efflux transporter may contribute to IVM transport across the BBB in other species (38) and may therefore represent an additional candidate for genetically mediated drug sensitivity in passerine birds. Given the lack of systematic *in vivo* data on IVM-induced neurotoxicity in the Eurasian bullfinch, the possibility of an increased susceptibility at the level of the target receptor remains unresolved.

### Sequence insertion in the ABCB1 linker region in passerine birds

Although the present study did not identify a deleterious mutation in the *ABCB1* gene of the Eurasian bullfinch, an intriguing genetic feature was observed: a 10-aa insertion motif in the ABCB1 linker region, present in one, two, or three tandem repeats. *In vitro*, the tandem-repeat insertion in Eurasian bullfinch WT ABCB1 significantly reduced total protein expression in transfected HEK293 cells but did not impair transporter efflux activity. A limitation of this study is that only total protein levels were measured by Western blot, without direct quantification of the ABCB1 protein fraction functionally expressed at the plasma membrane. Therefore, it is possible that the tandem repeat insertion affects the overall protein abundance in the transfected HEK293 cells while leaving the membrane-localized ABCB1 transporter fraction unchanged. To address this question, more sophisticated methods are necessary to analyze the sorting pathway of the ABCB1 protein and its distribution to different cell compartments. This could not be achieved in the present study.

The ABCB1 linker region of about 75 aa was long regardeded as a structural element connecting the two halves of the protein (39). However, subsequent studies identified phosphorylation sites, suggesting a regulatory role (40), and a tubulin-binding motif, indicating involvement in protein interactions and potential roles in signaling and protein stability (41). Proteolytic cleavage of the linker in the purified human ABCB1 protein altered the transport kinetics by increasing the ATPase activity while decreasing the substrate specificity (42). Hrycyna et al. (43) demonstrated that deletions within the linker can dramatically impair ATP hydrolysis and transport without affecting substrate binding, whereas insertions of certain sequences yielded a functional protein if the structural flexibility was maintained. These findings highlight that the secondary and tertiary structures, rather than the primary sequence at the linker region, are relevant for ABC transporter function, explaining the generally low sequence conservation in the ABCB1 linker region between species (44). Based on this, closer transport kinetic characterization and determination of protein synthesis and turnover rates of the three Eurasian bullfinch ABCB1 variants should be analyzed in subsequent studies. These analyses would require more sophisticated protein expression and analysis methods and were out of the scope of the present study.

Regarding the genetic and evolutionary origin of the identified insertion variants in the ABCB1 linker region, one can speculate about replication slippage or unequal crossing-over events during meiosis, as previously reported (45, 46). Interestingly, higher evolutionary dynamics of repetitive elements are described for bird genomes compared to mammals (47). As similar linker structures are also present in other members of the ABC family (48), a more detailed sequence analysis expanding the group of *Passeriformes* species and including ABCB1-related transporter proteins might be of interest for subsequent studies. However, even if the ABCB1 insertion variants characterized in the present study showed a predominant appearance within specific *Passeriformes* families, there were some exceptions in the variant pattern within other *Passeriformes* families. This supports the high evolutionary dynamics of repetitive elements in birds.

### Limitations of the *in vitro* Rh123 transport assays to predict *in vivo* IVM drug tolerance

Rh123 is a commonly used fluorescent tracer dye for *in vitro* transport assays aimed at assessing the ABCB1 efflux function (14, 30, 49). Previous studies have shown that factors such as membrane potential and mitochondrial abundance can influence the intracellular sequestration and metabolism of Rh123 (50, 51). These processes complicate the interpretation of experimental data as well as comparisons across different cell lines. In addition, HEK293 cells show a low basal expression of the endogenous human ABCB1 transporter protein (30) that contributes to the Rh123 efflux in all transfected and non-transfected HEK293 cells. Furthermore, it has to be considered that minor leakage of the TET repressor might occur even in the absence of TET, as previously reported (34). This can result in a minor background expression of the recombinant Eurasian bullfinch ABCB1 protein even under -TET conditions. Despite these limitations, transport assays with Rh123 remain useful for obtaining a general assessment of overall transporter activity when the appropriate controls are used. In the present study, we used three types of controls. Firstly, we used TET-treated cells in which the respective ABCB1 carrier variants are overexpressed and compared them with non-TET-treated cells. In this assay, all ABCB1 variants from the Eurasian bullfinch showed significant and comparable Rh123 efflux activity. Specificity of this ABCB1-mediated Rh123 efflux was controlled by additionally analyzing HEK293 cells overexpressing the mutant canine ABCB1 that is functionally inactive (30). Accordingly, in this cell line, no TET-induced decline of Rh123 efflux could be detected. As an additional control, we used the ABCB1 inhibitor TQR that blocked any ABCB1 efflux activity from the overexpressed recombinant ABCB1 transporter protein variants, as well as from the endogenously expressed human ABCB1. Accordingly, under TQR treatment, all cells accumulated significantly higher amounts of Rh123 compared with the non-TQR-treated cells. This increase in Rh123 fluorescence can be explained by complete TQR blockade of the endogenously expressed human ABCB1 transporter protein, as well as TQR blockade of some residual recombinant Eurasian bullfinch ABCB1 transporter proteins expressed from leaky TET promoters even in the absence of TET (34).

However, even with this well-controlled experimental setup, the assessment of transporter kinetic characteristics is limited in the Rh123 assay. Furthermore, direct transport experiments with IVM would have been more meaningful in the context of the present study. However, running a direct transport assay in transfected HEK293 cells with IVM as the probe substrate was not successful in the present study. In this assay, we could not obtain robust and reproducible data, even under different experimental conditions, such as different IVM substrate concentrations and different incubation times in the uptake and/or efflux phases (data not shown). Such assays were previously reported only for transepithelial transport experiments using, for example, Caco-2 cells (52). Due to this limitation, we took advantage of the double role of IVM as substrate and inhibitor of ABCB1 (53–55) and analyzed IVM as an inhibitor of the ABCB1-mediated Rh123 efflux in the stably transfected HEK293 cells. This assay has recently been used to characterize the interaction of IVM with the ABCB1 transporter proteins from dog and cat (30). In this assay, we obtained comparable IC_50_ values for IVM for the Eurasian bullfinch WT and Δ1/Δ2 mutant ABCB1 transporter variants, indicating that the linker insertion does not measurably affect the ability of IVM to inhibit the ABCB1-mediated efflux of Rh123. Moreover, as the IC_50_ values for IVM were comparable between the canine, feline, and Eurasian bullfinch ABCB1, the IVM inhibitor binding site seems to be highly conserved among these homologous transporter proteins.

## Conclusion

In the present study, sequence analysis of the Eurasian bullfinch ABCB1 revealed a linker insertion apparently unique to some *Passeriformes* species. This insertion reduced the total ABCB1 protein expression in HEK293 cells, but this had no functional consequence for the Rh123 efflux activity of the WT and Δ1/Δ2 mutant Eurasian bullfinch ABCB1 transporter variants, when expressed in HEK293 cells. Moreover, all variants showed comparable IC_50_ values for TQR and IVM as inhibitors of the ABCB1-mediated Rh123 efflux. Based on this, the *in vitro* data of the present study do not support any role of this sequence insertion in the ABCB1 linker region for the suspected IVM sensitivity in the Eurasian bullfinch.

## Data Availability

The datasets presented in this study can be found in the following online repository: https://www.ncbi.nlm.nih.gov/genbank/, with the accession numbers listed in Table 2.
