# Supplementary material for: Cloning, structural and functional characterization of the ABCB1 transporter of the Eurasian bullfinch (Pyrrhula pyrrhula)
Source: Front Vet Sci. 2026 Apr 10;13:1759019. doi: 10.3389/fvets.2026.1759019 (PMC13105923; doi:10.3389/fvets.2026.1759019)
Supplement: Supplementary file 1 [file Data_Sheet_1.docx]

Cloning, structural and functional characterization of the Eurasian bullfinch (*Pyrrhula pyrrhula*) ABCB1 transporter

Katharina Heilmann^1^, Luise Charlotte Kempf^1^, Lisa Siegl^1^, Melanie Hamann^1^, Michael Lierz^2^, Joachim Geyer^1^

*^1^Institute of Pharmacology and Toxicology, Faculty of Veterinary Medicine, Biomedical Research Center Seltersberg (BFS), Justus Liebig University of Giessen, Schubertstr. 81, 35392 Giessen, Germany*

*^2^Clinic for Birds, Reptiles, Amphibians and Fish, Faculty of Veterinary Medicine, Justus Liebig University of Giessen, Frankfurter Strasse 114, Giessen 35392, Germany*

**Short running title:** Characterization of bullfinch ABCB1

**Supplementary Material**

1. **Supplementary Figure 1: Multi sequence alignment of ABCB1 from *Passeriformes* species**
2. **Supplementary Figure 2: Western blot scans**
3. **Supplementary Table 1: Adjusted band volumes of the Western blot analysis**
4. **Supplementary Table 2: Confidence intervals for IC_50_ calculation**

**CDM63410.1 1 MPSEDEKK-YTADGNSHEITASSQGHESEDKHMAKKK-KKGEKPKVVSPFALFRYSSWSDKLLMILGTLLAVAHGSSLPF 78**

**XP_059698659.1 1 MHSEDEKK-YTADGNSHEIAASSQGHESEDKHKAKKKQKKGEKPKVVSPFALFRYSSWSDKLLMILGTLLAVAHGSSLPF 79**

**XP_050827218.1 1 MHSEDEKK-YAADGNSHEIAASSQGHESEDKHKSKKKQKKGEKPKVVSPFALFRYSSSSDKLLMILGTLLAVAHGSSLPI 79**

**XP_037984436.1 1 MHSEDEKK-YTADGNSHEIAASSQGHESEDKHKAKK-QKKGEKPKVVSPFTLFRYSSWSDKLLMILGTLLAVAHGSSLPF 78**

**XP_041322342.1 1 MHSEDEKK-HTADGNSHEIAASSQDHESEDK-QAKKKQKKGEKPKVVSPFALFRYSSWSDKLLMILGTLLAIAHGSSLPF 78**

**XP_041264549.1 1 MHSEEEKK-YTADGNSHEIAASSQDHESEDKHRAKKKQKKGEKPKVVSPFALFRYSSWSDKLLMILGTLMAIAHGSSLPF 79**

**XP_039567137.1 1 MHSEDEKK-CTADGNSHEITAASQDDESEDKHKAKKKQKKGEKPKVVSPFALFRYSSWSDKLLMILGTLLAVAHGSSLPF 79**

**XP_064265165.1 1 MHSEDEKK-CSADGNSHEIAAASQDDESEDKHKAKKKQKKGEKPKVVSPFALFRYSSWSDKLLMILGTLLAVAHGSSLPF 79**

**XP_016151870.1 1 MHSEDEKK-YTADGNSHEIAASSQDHESEDKHKAKKKQKKGEKPKMVSPFTLFRYSSWSDKLLMILGTLLAVAHGSSLPI 79**

**XP_058670934.1 1 MPSEDEKK-YTADGNSHEIAASSPGHESEDKHKAKKKQKKGEKPKVVSPFALFRYSSWSDKLLMILGTVMAVAHGSSLPF 79**

**XP_059323184.1 1 MPSEDEKK-YTADGNSHEIAASSPGHESEDKHKAKKKQKKGEKPKVVSPFALFRYSSWSDKLLMILGTVMAVAHGSSLPF 79**

**XP_057876310.1 1 MPSEDEKK-YTADGNSHEIAASSPGHESEDKHKAKKKQKKGEKPKVVSPFALFRYSSWSDKLLMILGTVMAVAHGSSLPF 79**

**XP_063006839.1 1 MPSEDEKK-YSADGNSHEIAASSPGHESEDKHKAKTKQKKGEKPKVVSPFALFRYSSWSDKLLMILGTVMAVAHGSSLPF 79**

**XP_053808094.1 1 MHSENEK--YTGDGNSNEIVASSQGHESEDKHKAKKKQKKGEKPKVVSPFTLFRYSSWSDKLLMILGTLLAVAHGSSLPF 78**

**XP_068068048.1 1 MHSENEK--YTGDGNSHEIAASSQGHESEDKHKAKK-QKKGEKPKVVSPFTLFRYSSWSDKILMILGTLLAVAHGSSLPF 77**

**XP_004186266.5 1 MHSEDEKK-YTEDGNSHGIAASSQGHELEDKDKAKKKQKKGEKPKVVSPFTLFRYSSWSDKLLMILGTLLAIAHGSSLPF 79**

**XP_021407324.2 1 MHSEDKKK-YTEDGNSHEIAASSQGHELEDKEKTEKKQKKGEKPKVVSPFTLFRYSSWSDKLLMILGTLLAVAHGSSLPF 79**

**XP_054129694.1 1 MPSEDEKK-YTADGNSHEIASSSQGHESEDKHKAKKKQKKGEKPKVVSPFALFRYSSWSDKLLMIVGTVMAVAHGSSLPF 79**

**XP_064560295.1 1 MPSEDEKK-YTADGNSHEIAASNQGHESEDKHKAKKKQKKGEKPKVVSPFALFRYSSWSDKLLMILGTVMAVAHGSSLPF 79**

**XP_074391572.1 1 MPSEDEKK-YTADGNSHEIAASNQDHESEDKHKAKKKQKKGEKPKVVSPFALFRYSSWSDKLLMILGTVTAVAHGSSLPF 79**

**XP_056339284.1 1 MLSEDEKK-YTADGNSHEIAASSQDHESEDKHKAKKKQKKGEKPKMVSPFTLFRYSSWSDKLLMILGTLLAVAHGSSLPI 79**

**XP_036262392.1 1 MHSEDEKK-YTADGNSHEIIASSQGHESEDKHKAKKKQKKGEKPKVVSPFALFRYSSWSDKLLMILGTVMAVAHGSSLPF 79**

**XP_066408995.1 1 MHSEDEKK-YTADGNSHEIIASSQGHESEDKHKAKKKQKKGEKPKVVSPFALFRYSSWSDKLLMILGTVMAVAHGSSLPF 79**

**XP_054484009.2 1 MHSEDEKK-YTADGNSHEIIASSQGHESEDKHKAKKKQKKGERPKVVSPFALFRYSSWSDKFLMILGTVMAVAHGSSLPF 79**

**XP_071285034.1 1 MHSEDEKK-YTADGNSHEIIASSQGHESEDKHKAKKKQKKGEKPKVVSPFALFRYSSWSDKFLMILGTVMAVAHGSSLPF 79**

**XP_030922622.1 1 MHSVDEKK-YTADGNSHEIAASSQGHESEDKHKEKKKQKKGEKPKVVSPFALFRYSSWSDKLLMILGTVLAVAHGSSLPF 79**

**XP_030826393.1 1 MHSVDEKK-YTADGNSHEIAASSQGHESEDKHKEKKKQKKGEKPKVVSPFALFRYSSWSDKLLMILGTVLAVAHGSSLPF 79**

**XP_062350659.1 1 MHSEDENK-YTADGNSHEIAASSQD--DEDKHKAKKKQKKGEKPKMVSPFTLFRYSSRSDKLLMILGTLLAVAHGSSLPI 77**

**XP_014731144.1 1 MHSEDEKK-YTADGNSHEIAASSQDHESEDKHKEKKKKKKKEKPKMVSPFTLFRYSSWSDKSLMILGTLMAVAHGSSLPI 79**

**XP_032926696.1 1 MHSEDEKK-YTADRNSHELTASSQDHEPEDKHKAKK-QKKGEKPKVVSPLALFRYSSWSDKLLMLLGTLLAIAHGSSLPI 78**

**XP_005518835.2 1 MPSEDEKK-YSADGNSHEIAASSQDHESEDKEKTKKKKK-GEKPKVVSPFTLFRYSSWSDKLLMILGTVLAVAHGSSLPI 78**

**XP_058687902.1 1 MPSEDEKK-YSADGNSHEIAASSQDHESEDKEKAKKKKKNGEKPKVVSPLTLFRYSSWSDKLLMILGTVLAVAHGSSLPI 79**

**XP_015471143.1 1 MPSEDEKK-YSADGNSHEIAAFSQDHESEDKEKTKKKKKKGEKPKVVSPFTLFRYSTWSDKLLMILGTVLAVAHGSSLPI 79**

**XP_023776043.1 1 MPSEDEKK-YSADGNSHDIAASSQDHESEDKEKAKKKKKKGEKPKVVSPFTLFRYSSWSDKLLMILGTVLAVAHGSSLPI 79**

**XP_058275891.1 1 MNSEDEKK-YTADGNHHEIAASSQGHESEDKHREKKKQKKGEKPKMVSPFTLFRYSSWSDKLLMILGTLLAVAHGSSLPF 79**

**XP_063268876.1 1 MPSEDEKK-YTADGNSHELAASSQGHESEDQHKAKKKQKKGEKPKVVSPLTLFRYSSWSDKLLMILGTLLAVVHGSSLPV 79**

**XP_068862854.1 1 MPSEDEKK-YTADGNSHEIAASSQDHEFEDKHQGKKKQKKGEKPKMVSPLTLFRYSSWSDKLLMILGTLLAVAHGSSLPF 79**

**XP_031968992.1 1 MPSEDEKK-YTADGNSHEIAASSQGHEFEDKHQGKKKQKKGEKPKTVSPLALFRYSSWSDKLLMILGTLLAVAHGSSLPF 79**

**XP_010397518.2 1 MPSEDEKK-YTADGNSHEIAASSQGHEFEDKHQGKKKQKKGEKPKTVSPLALFRYSSWSDKLLMILGTLLAVAHGSSLPF 79**

**XP_008628111.1 1 MPSEDEKK-YTADGNSHEIAASSQGHEFEGKHQGKKKQKKGEKPKTVSPLALFRYSSWSDKLLMILGTLLAVAHGSSLPF 79**

**XP_041899696.1 1 MPSEDEKK-YTADGNSHEIAASSQGHEFEDKHQGKKKQKKGEKPKTVSPLALFRYSSWSDKLLMILGTLLAVAHGSSLPF 79**

**XP_048162577.1 1 MPSEDEKK-YTADGNSHEIAASSQGHEFEDKHQGKKKQKKGEKPKTVSPLALFRYSSWSDKLLMILGTLLAVAHGSSLPF 79**

**XP_066172927.1 1 MHSGDEKR-YTADGNSYEIVASSQGNESEDKHKAKKKQKKGEKPKMVSPFTLFRYSSWSDKLLMILGTLLAVAHGSSLPF 79**

**XP_066054226.1 1 MHSGDEKN-YTADGNSHEIAAFSQGDESEDIHKAKKKQKKGEKPKMVSPFTLFRYSSCSDKLLMILGTLLAVAHGSSLPI 79**

**XP_057239101.1 1 MHSEDEKX-YTADGNSHEITPFSQGHESEDKDKEKKK-KKAEKPKMVSPFTLFRYSSWSDKLLMILGTLLAVAHGSSLPI 78**

**XP_039235593.1 1 MHSEDEKN-YTADGNIHDVA--NQGHESEDKQKKKKKQKKGEKPKVVSPFALFRYSTWSDKWLMILGTIMAAAHGSSLPI 77**

**XP_027564162.1 1 MNSEDEKNNYTADGNSHDVA--SQGHGSEDKHKKNKKQKKGEKPKVVSPFALFRYSTCSDKLLMILGTILAVAHGSALPI 78**

**XP_051658500.1 1 MHSEDEKN-YTADGNIHDVA--NQGHESEDKQKKKKKQKKGEKPKVVSPFALFRYSTWSDKWLMILGTILAVAHGSALPI 77**

**XP_064534663.1 1 MHSEDEKN-YTADGNIHDVA--NQGHESEDKQKKKKKQKKGEKPKVVSPFALFRYSTWSDKMLMILGTILAAAHGSALPI 77**

**CDM63410.1 79 AMIIFGDMTDSFV---TSGDTNFTGNVS--------TNLTSDML------------------NKLEEDMTRYAYYYSGIA 129**

**XP_059698659.1 80 AMIIFGDMTDSFV---ASGDTNFTGNIS--------MNLTSEML------------------NKLEEDMTRYAYYYSGIA 130**

**XP_050827218.1 80 AMVIFGDMTDSFV---ASGDTNFTGNTS--------TNFTSEML------------------HKLEEDMTRYAYYYSGIA 130**

**XP_037984436.1 79 AMIIFGDMTDSFV---ASGDTNFTGNFS--------MNLTSDML------------------GKLEEDMTRYAYYYSGIA 129**

**XP_041322342.1 79 AMIIFGDMTDSFV---ASGDTNFTGNIS--------MNFTSEML------------------HKLEEDMTRYAYYYSGIA 129**

**XP_041264549.1 80 AMIIFGDMTDSFV---ASGDTNFTGNSS--------MNFTSEML------------------HKLEEDMTRYAYYYSGIA 130**

**XP_039567137.1 80 AMIIFGDMTDSFV---ASGDTNFTGNIS--------INFTSEIL------------------HKLEDDMTRYAYYYSGIA 130**

**XP_064265165.1 80 AMIIFGDMTDSFV---ASGDTNFTGNIS--------INLTSQIL------------------HKLEEDMTRYAYYYSGIA 130**

**XP_016151870.1 80 AMIIFGDMTDSFV---ASGDKNFTGNFS--------MNFTSEIL------------------NKLEEDMTRYAYYYSGIA 130**

**XP_058670934.1 80 AMMIFGDMTDSFV---AFGEKNFTGNSSVNEL----MNVTAEML------------------SQLEEDMTRYAYYYSGIA 134**

**XP_059323184.1 80 AMMIFGDMTDSFV---AFGEKNFTGNSSVNEL----MNVTAEML------------------SQLEEDMTRYAYYYSGIA 134**

**XP_057876310.1 80 AMMIFGDMTDSFV---GSGEKNLTENSSMNEL----TNVTAEMF------------------NQLEKDMTRYAYYYSGIA 134**

**XP_063006839.1 80 AMMIFGDMTDSFV---GSGEKNFSENSSMDEL----KNVTAEML------------------NQLEEDMTRYAYYYSGIA 134**

**XP_053808094.1 79 AMIIFGDMTDSFV---ASGDKNFTGNTS--------LNFTSDML------------------DKLEEDMTRYAYYYSGIA 129**

**XP_068068048.1 78 AMIIFGDMTDSFV---ASGDKNFTGNAS--------LNFTSDML------------------NKLEEDMTRYAYYYSGIA 128**

**XP_004186266.5 80 AMIIFGDMTDSFV---SSGDKNPTGDFS--------QNFTSDML------------------HKLEEDMTRYAYYYSGIA 130**

**XP_021407324.2 80 AMIIFGDMTDSFV---ASEDKNSTGKFS--------QNFTSDML------------------HKLEEDMTRYAYYYSGIA 130**

**XP_054129694.1 80 AMMIFGDMTDSFV---ASGEKNFTGNSSMEEW----MNVTAEML------------------NQLEEDMTRYAYYYSGIA 134**

**XP_064560295.1 80 AMMIFGDMTDSFVMTDTSGEKNLTVNSSMDEW----MNVTADML------------------NQLEADMTRYAYYYSGIA 137**

**XP_074391572.1 80 AMMIFGDMTDSFVMTDTSGEKNLTVNSSMDEW----MNVTADML------------------NQLEADMTRYAYYYSGIA 137**

**XP_056339284.1 80 AMIIFGDMTDSFV---ASGDTNFTGNFS--------MNFTSEVL------------------NKLEEDMTRYAYYYSGIA 130**

**XP_036262392.1 80 AMMIFGDMTDSFV---ASGNKNFTEDPTINEW----MNITSEVL------------------NKLEEEMTRYAYYYSGIA 134**

**XP_066408995.1 80 AMMIFGDMTDSFV---ASGNKNFTEDPTINEW----MNITSEVL------------------NKLEEEMTRYAYYYSGIA 134**

**XP_054484009.2 80 AMMIFGDMTDSFV---ASANKNLTEDPTIDDW----MNITSDML------------------NKLEKEMTRYAYYYSGIG 134**

**XP_071285034.1 80 AMMIFGDMTDSFV---ASANKNLTEDPTINDW----MNITSDML------------------NKLEKEMTRYAYYYSGIG 134**

**XP_030922622.1 80 AMMIFGDMTDSFV---ASGDKEFTGNSSMDEW----MNFTSEML------------------NKLEEDMTRYAYYYSGIA 134**

**XP_030826393.1 80 AMMIFGDMTDSFV---ASGDKEFTGNSSMDEW----MNFTSEML------------------NKLEEDMTRYAYYYSGIA 134**

**XP_062350659.1 78 AMIIFGDMTDSFV---ASGDRNFTENIP--------LNFTPDIL------------------NKLEEDMTRYAYYYSGIA 128**

**XP_014731144.1 80 AMIIFGDMTDSFV---ASEGRNDTGKNL--------TNITSEIF------------------NNLEENMTRYAYYYSGIA 130**

**XP_032926696.1 79 AMKIFGDMTDSFITP-----GNFTGNFSSDELKNLSMNSTSDML------------------NQLEENMTRYAYYYSGIA 135**

**XP_005518835.2 79 AMIIFGDMTDSFV---ASGNNNFTGNIS--------MNFTSDML------------------DKLEEDMTRYAYYYSGIA 129**

**XP_058687902.1 80 AMIIFGDMTDSFV---ASGNNNFTGNVS--------MNFTSDML------------------DKLEEDMTRYAYYYSGIA 130**

**XP_015471143.1 80 AMIIFGDMTDSFV---ASGNNNFTGNIS--------MNFTSDML------------------DKLEEDMTRYAYYYSGIA 130**

**XP_023776043.1 80 AMVIFGDMTDSFV---ASGNNNFTGNIS--------MNITSDML------------------DKLEEDMTRYAYYYSGIA 130**

**XP_058275891.1 80 AMIIFGDMTDSFV---ASGDKNFS------------VNFTEGML------------------HKLEEDMTRYAYYYSGIA 126**

**XP_063268876.1 80 AMVIFGDMTDSFV---ASGNKSFAGNISL-------QNFTSEIL------------------HKLEEDMTRYAYYYSAIA 131**

**XP_068862854.1 80 AMIIFGDMTDSFV---TSGDTNFTGNISM--------NFTHDML------------------EKLEEDMTRYAYYYSGIA 130**

**XP_031968992.1 80 AMIIFGDMTDSFV---ISGDTNFTGNFSM--------NFTHDMF------------------EKLEEDMTRYAYYYSGIA 130**

**XP_010397518.2 80 AMIIFGDMTDSFV---ISGDTNFTGDFSM--------NFTHNML------------------ENLEEDMTRYAYYYSGIA 130**

**XP_008628111.1 80 AMIIFGDMTDSFV---ISGDTNFTGNFSM--------NFTHNML------------------EDLEEDMTRYAYYYSGIA 130**

**XP_041899696.1 80 AMIIFGDMTDSFV---ISGDTNFTGNFSM--------NFTHNMF------------------EKLEEDMTRYAYYYSGIA 130**

**XP_048162577.1 80 AMIIFGDMTDSFV---ISGDTNFTGDFSM--------NFTHNMF------------------EKLEEDMTRYAYYYSGIA 130**

**XP_066172927.1 80 AMIIFGDMTDSFV---ASGNTGSTGNNSM--------NFTEDMK-------NISMNFTAEMLHKLEEDMTRYAYYYSGIA 141**

**XP_066054226.1 80 AMVIFGDMTDSFV---ASGQKNFTGNSSM--------NFTEDMLPKPEDMTSNSTNVTADMLHKLEEDMTRYAYYYSGIA 148**

**XP_057239101.1 79 AMVIFGDMTDSFV---ASGDTNFTGNITI--------NITSAVL------------------HQLEEDMTRYAYYYSGIA 129**

**XP_039235593.1 78 AMIIFGDMTDSFV---ASGDLNFTGLNSSQ------MNFTSEML------------------EKLEEDMTRYAYYYCGIA 130**

**XP_027564162.1 79 AMIIFGDMTDSFV---ASGDLNSTGSNSSQ------MNFTSDML------------------EKLEEDMTRYAYYYCGIA 131**

**XP_051658500.1 78 AMIIFGDMTDSFV---ASGDLNFTGLNSSQ------MNFTADML------------------EKLEEDMTRYAYYYCGIA 130**

**XP_064534663.1 78 AMIIFGDMTDSFV---ASGDLNFTGLNSSQ------MNFTADML------------------EKLEEDMTRYAYYYCGIA 130**

**CDM63410.1 130 AGVLLAAYIQTSFWTLAAGRQIKKIREKFFHAIMRQEIGWFDVNDVGELNTRLLDDVSKINDGIGDKIGLLVQALTTFVT 209**

**XP_059698659.1 131 AGVLLAAYIQTSFWTLAAGRQIKKIRQKFFHAIMRQEIGWFDVNDVGELNTRLLDDVSKINDGIGDKIGLLVQALTTFVT 210**

**XP_050827218.1 131 AGVLLAAYIQTSFWTLAAGRQIKKIREKFFHAIMRQEIGWFDVNDVGELNTRLLDDVSKINDGIGDKVGLLVQALTTFVT 210**

**XP_037984436.1 130 AGVLLAAYIQTSFWTLAAGRQIKKIREKFFHAIMRQEIGWFDVNDVGELNTRLLDDVSKISEGIGDKIGLLVQALTTFVT 209**

**XP_041322342.1 130 AGVLLAAYIQTSFWTLAAGRQIKKIRENFFHAIMRQEIGWFDVNDVGELNTRLLDDVSKINEGIGDKIGLLVQALTTFVT 209**

**XP_041264549.1 131 AGVLLAAYIQTSFWTLAAGRQIKKIRENFFHAIMRQEIGWFDVNDVGELNTRLLDDVSKINEGIGDKIGLLVQALTTFVT 210**

**XP_039567137.1 131 AGVLLAAYIQTSFWTLAAGRQIKKIRENFFHAIMRQEIGWFDVNDVGELNTRLLDDVSKINEGIGDKIGLLVQALTTFVT 210**

**XP_064265165.1 131 AGVLLAAYIQTSFWTLAAGRQIKKIREKFFHAIMRQEIGWFDVNDVGELNTRLLDDVSKINEGIGDKIGLLVQSLTTFVT 210**

**XP_016151870.1 131 AGVLLAAYVQTSFWTLAAGRQIRKIREKFFHAIMRQEIGWFDVNDVGELNTRLLDDVSKINEGIGDKIGLLVQSLTTFVT 210**

**XP_058670934.1 135 AGVLLAAYIQTSFWTLAAGRQIKKIREKFFHAIMRQEIGWFDVNDVGELNTRLLDDVSKINDGIGDKVGLLVQALTTFVT 214**

**XP_059323184.1 135 AGVLLAAYIQTSFWTLAAGRQIKKIREKFFHAIMRQEIGWFDVNDVGELNTRLLDDVSKINDGIGDKVGLLVQALTTFVT 214**

**XP_057876310.1 135 AGVLLAAYIQTSFWTLAAGRQIKKIREKFFHAIMRQEIGWFDVNDVGELNTRLLDDVSKINDGIGDKVGLLVQALTTFVT 214**

**XP_063006839.1 135 AGVLLAAYIQTSFWTLAAGRQIKKIREKFFHAIMRQEIGWFDVNDVGELNTRLLDDVSKINDGIGDKVGLLVQALTTFVT 214**

**XP_053808094.1 130 AGALLAAYIQTSFWTLAAGRQIKKIREKFFHAIMRQEIGWFDVNDVGELNTRLLDDVSKINEGIGDKIGLLVQSLTTFVT 209**

**XP_068068048.1 129 AGVLLAAYIQTSFWTLAAGRQIKKIREKFFHAVMRQEIGWFDVNDVGELNTRLLDDVSKINDGIGDKIGLLVQSLTTFVT 208**

**XP_004186266.5 131 AGVLLAAYIQTSFWTLAAGRQIKKIRENFFHAIMRQEIGWFDVNDVGELNTRLLDDVSKINEGIGDKIGLLVQSLTTFVT 210**

**XP_021407324.2 131 AGVLLAAYIQTSFWTLAAGRQIKKIREKFFHAIMRQEIGWFDVNDVGELNTRLLDDVSKINEGIGDKIGLLVQSLTTFVT 210**

**XP_054129694.1 135 AGVLLAAYIQTSFWTLAAGRQIKKIREKFFHAIMRQEIGWFDVNDVGELNTRLLDDVSKINDGIGDKVGLLVQALTTFMT 214**

**XP_064560295.1 138 AGVLLAAYIQTSFWTLAAGRQIKKIREKFFHAIMRQEIGWFDVNDVGELNTRLLDDVSKINDGIGDKVGLLVQSLTTFVT 217**

**XP_074391572.1 138 AGVLLAAYIQTSFWTLAAGRQIKKIREKFFHAIMRQEIGWFDVNDVGELNTRLLDDVSKINDGIGDKVGLLIQSLTTFVT 217**

**XP_056339284.1 131 AGVLLAAYVQTSFWTLAAGRQIRKIREKFFHAIMRQEIGWFDVNDVGELNTRLLDDVSKINEGIGDKVGLLVQSLTTFVT 210**

**XP_036262392.1 135 AGVLLAAYIQTSFWTLAAGRQIKKIREKFFHAIMRQEIGWFDVNDVGELNTRLLDDVSKINDGIGDKIGLLVQALTTFVT 214**

**XP_066408995.1 135 AGVLLAAYIQTSFWTLAAGRQIKKIREKFFHAIMRQEIGWFDVNDVGELNTRLLDDVSKINDGIGDKIGLLVQALTTFVT 214**

**XP_054484009.2 135 AGVLLAAYIQTSFWTLAAGRQIKKIRENFFHAIMRQEIGWFDVNDVGELNTRLLDDVSKINDGIGDKIGLLVQALTTFVT 214**

**XP_071285034.1 135 AGVLLAAYIQTSFWTLAAGRQIKKIRENFFHAIMRQEIGWFDVNDVGELNTRLLDDVSKINDGIGDKIGLLVQALTTFVT 214**

**XP_030922622.1 135 AGVLLAAYIQTSFWTLAAGRQIKKIRENFFHAIMRQEIGWFDVNDVGELNTRLLDDVSKINDGIGDKVGLLVQALTTFVT 214**

**XP_030826393.1 135 AGVLLAAYIQTSFWTLAAGRQIKKIRENFFHAIMRQEIGWFDVNDVGELNTRLLDDVSKINDGIGDKVGLLVQALTTFVT 214**

**XP_062350659.1 129 AGVLLAAYIQTSFWTLAAGRQIKKIREHFFHAIMRQEIGWFDVNDVGELNTRLLDDVSKINEGIGDKVGLLVQSLTTFVA 208**

**XP_014731144.1 131 AAVLLAAYVQTSFWTLAAGRQIKKIRENFFHAIMRQEIGWFDVNDVGELNTRLLDDVSKINEGIGDKVGLLVQSVTTFVT 210**

**XP_032926696.1 136 VGVLIAAYVQTAFWTLAAGRQIKRIREKFFHAIMRQEIGWFDVNDAGELNTRLLDDVSKINEGIGDKIGLLVQSVTTFVA 215**

**XP_005518835.2 130 AGVLLAAYIQTSFWTLAAGRQIKKIREKFFHAIMRQEIGWFDVNDVGELNTRLLDDVSKINDGIGDKIGLLVQALTTFVT 209**

**XP_058687902.1 131 AGVLLAAYIQTSFWTLAAGRQIKKIREKFFHAIMRQEIGWFDVNDVGELNTRLLDDVSKINDGIGDKIGLLVQALTTFVT 210**

**XP_015471143.1 131 AGVLLAAYIQTSFWTLAAGRQIKKIREKFFHAIMRQEIGWFDVNDVGELNTRLLDDVSKINDGIGDKIGLLVQALTTFVT 210**

**XP_023776043.1 131 AGVLLAAYIQTSFWTLAAGRQIKKIREKFFHAIMRQEIGWFDVNDVGELNTRLLDDVSKINDGIGDKIGLLVQALTTFVT 210**

**XP_058275891.1 127 AGVLLAAYIQTSFWTLAAGRQIKKIREKFFHAIMRQEIGWFDVNDVGELNTRLLDDVSKINDGIGDKIGLLVQALTTFVT 206**

**XP_063268876.1 132 AGVLLAAYIQTSFWTLAAGRQIKKIREKFFHAIMRQEIGWFDVNDVGELNTRLLDDVSKINDGIGDKIGLLVQAITTFVT 211**

**XP_068862854.1 131 AGVLLAAYVQTSFWTLAAGRQIKKIREKFFHAIMRQEIGWFDVNDVGELNTRLIDDVSKINEGIGDKIGLLVQSLTTFVV 210**

**XP_031968992.1 131 AGVLLAAYVQTSFWTLAAGRQIKKIREKFFHAIMRQEIGWFDVNDVGELNTRLLDDVSKINEGIGDKMGLLVQSLTTFVV 210**

**XP_010397518.2 131 AGVLLAAYVQTSFWTLAAGRQIKKIREKFFHAIMRQEIGWFDVNDVGELNTRLLDDVSKINEGIGDKMGLLVQSLTTFVV 210**

**XP_008628111.1 131 AGVLLAAYVQTSFWTLAAGRQIKKIREKFFHAIMRQEIGWFDVNDVGELNTRLLDDVSKINEGIGDKMGLLVQSLTTFVV 210**

**XP_041899696.1 131 AGVLLAAYVQTSFWTLAAGRQIKKIREKFFHAIMRQEIGWFDVNDVGELNTRLLDDVSKINEGIGDKMGLLVQSLTTFVV 210**

**XP_048162577.1 131 AGVLLAAYVQTSFWTLAAGRQIKKIREKFFHAIMRQEIGWFDVNDVGELNTRLLDDVSKINEGIGDKMGLLVQSLTTFVV 210**

**XP_066172927.1 142 AGVLVAAYIQTSFWTLAAGRQIKKIREKFFHAIMRQEIGWFDVNDVGELNTRLLDDVAKINEGIGDKIGLLVQSITTFVA 221**

**XP_066054226.1 149 AGVLLAAYIQTSFWTLAAGRQIKKIREKFFHAIMRQEIGWFDVNDVGELNTRLLDDVSKINEGIGDKIGLLVQAVTTFVT 228**

**XP_057239101.1 130 AGVLLAAYVQTSFWTLAAGRQIRKIREKFFHAIMRQEIGWFDVNDVGELNTRLLDDVSKINEGIGDKIGLLVQALTTFVT 209**

**XP_039235593.1 131 AGVLLAAYLQTSFWTLTAGRQIKKIREKFFHAIMRQEIGWFDVNDVGELNTRLLDDVSKINEGIGDKVGLLVQQLTAFVA 210**

**XP_027564162.1 132 AGVLLAAYIQTSFWTLTAGRQIKKIREKFFHAIMRQEIGWFDVNDVGELNTRLLDDVSKINEGIGDKVGLLIQQLTAFVA 211**

**XP_051658500.1 131 AGVLLAAYVQTSFWTLTAGRQIKKIREKFFHAIMRQEIGWFDVNDVGELNTRLLDDVSKINEGIGDKVGLLVQQLTAFVA 210**

**XP_064534663.1 131 VGVLLAAYLQTSFWTLTAGRQIKKIREKFFHAIMRQEIGWFDVNDVGELNTRLLDDVSKINEGIGDKVGLLVQQLTAFVT 210**

**CDM63410.1 210 GFIVGLIRGWKLTLVILAVSPVLGLSAALWAKVLSAFTDKEQAAYAKAGAVAEEVLGAIRTVIAFGGQEKEIKRYHKNLE 289**

**XP_059698659.1 211 GFIVGLIRGWKLTLVILAVSPVLGLSAALWAKVLSAFTDKEQAAYAKAGAVAEEVLGAIRTVIAFGGQEKEIKRYHKNLE 290**

**XP_050827218.1 211 GFIVGLIRGWKLTLVILAVSPVLGLSAALWAKVLSAFTDKEQAAYAKAGAVAEEVLGAIRTVIAFGGQEKEIKRYHKNLE 290**

**XP_037984436.1 210 GFIVGLIRGWKLTLVILAVSPVLGLSAALWAKVLSAFTDKEQAAYAKAGAVAEEVLAAIRTVIAFGGQEKEIKRYHKNLE 289**

**XP_041322342.1 210 GFIVGLIRGWKLTLVILAVSPVLGLSAALWAKVLSAFTDKEQAAYAKAGAVAEEVLAAIRTVIAFGGQEKEIKRYHKNLE 289**

**XP_041264549.1 211 GFIVGLIRGWKLTLVILAVSPVLGLSAALWAKVLSAFTDKEQAAYAKAGAVAEEVLAAIRTVIAFGGQEKEIKRYHKNLE 290**

**XP_039567137.1 211 GFVVGLIRGWKLTLVILAVSPVLGLSAALWAKVLSAFTDKEQAAYAKAGAVAEEVLAAIRTVIAFGGQEKEIKRYHKNLE 290**

**XP_064265165.1 211 GFIVGLIRGWKLTLVILAVSPVLGLSAALWAKVLSAFTDKEQAAYAKAGAVAEEVLAAIRTVIAFGGQEKEIKRYHKNLE 290**

**XP_016151870.1 211 GFVVGLIRGWKLTLVILAVSPVLGLSAALWAKVLSAFTDKEQAAYAKAGAVAEEVLGAIRTVIAFGGQEKEIKRYHKNLE 290**

**XP_058670934.1 215 GFIVGLIRGWKLTLVILAVSPVLGLSAALWAKVLSAFTDKEQAAYAKAGAVAEEVLAAIRTVIAFGGQEKEIKRYHKNLE 294**

**XP_059323184.1 215 GFIVGLIRGWKLTLVILAVSPVLGLSAALWAKVLSAFTDKEQAAYAKAGAVAEEVLAAIRTVIAFGGQEKEIKRYHKNLE 294**

**XP_057876310.1 215 GFIVGLIRGWKLTLVILAVSPVLGLSAALWAKVLSAFTDKEQAAYAKAGAVAEEVLAAIRTVIAFGGQEKEIKRYHKNLE 294**

**XP_063006839.1 215 GFIVGLIRGWKLTLVILAVSPVLGLSAALWAKVLSAFTDKEQAAYAKAGAVAEEVLAAIRTVIAFGGQEKEIKRYHKNLE 294**

**XP_053808094.1 210 GFIVGLIRGWKLTLVILAVSPVLGLSAALWAKVLSAFTDKEQAAYAKAGAVAEEVLAAIRTVIAFGGQEKEIKRYHKNLE 289**

**XP_068068048.1 209 GFIVGLIRGWKLTLVILAVSPVLGLSAALWAKVLSAFTDKEQAAYAKAGAVAEEVLAAIRTVIAFGGQEKEIKRYHKNLE 288**

**XP_004186266.5 211 GFIVGLIRGWKLTLVILAVSPVLGLSAALWAKVLSAFTDKEQAAYAKAGAVAEEVLAAIRTVIAFGGQEKEIKRYHKNLE 290**

**XP_021407324.2 211 GFIVGLIRGWKLTLVILAVSPVLGLSAALWAKVLSAFTDKEQAAYAKAGAVAEEVLAAIRTVIAFGGQEKEIKRYHKNLE 290**

**XP_054129694.1 215 GFIVGLIRGWKLTLVILAVSPVLGLSAALWAKVLSAFTDKEQAAYAKAGAVAEEVLAAIRTVIAFGGQEKEIKRYHKNLE 294**

**XP_064560295.1 218 GFIVGLIRGWKLTLVILAVSPVLGLSAALWAKVLSAFTDKEQAAYAKAGAVAEEVLAAIRTVIAFGGQEKEIKRYHKNLE 297**

**XP_074391572.1 218 GFIVGLIRGWKLTLVILAVSPVLGLSAALWAKVLSAFTDKEQAAYAKAGAVAEEVLAAIRTVIAFGGQEKEIKRYHKNLE 297**

**XP_056339284.1 211 GFVVGLIRGWKLTLVILAVSPVLGLSAAIWAKVLSAFTDKEQAAYAKAGAVAEEVLGAIRTVIAFGGQEKEIKRYHKNLE 290**

**XP_036262392.1 215 GFIVGLIRGWKLTLVILAVSPVLGLSAALWAKVLSAFTDKEQAAYAKAGAVAEEVLAAIRTVIAFGGQEKEIKRYHKNLE 294**

**XP_066408995.1 215 GFIVGLIRGWKLTLVILAVSPVLGLSAALWAKVLSAFTDKEQAAYAKAGAVAEEVLAAIRTVIAFGGQEKEIKRYHKNLE 294**

**XP_054484009.2 215 GFIVGLIRGWKLTLVILAVSPVLGLSAALWAKVLSAFTDKEQAAYAKAGAVAEEVLAAIRTVIAFGGQEKEIKRYHKNLE 294**

**XP_071285034.1 215 GFIVGLIRGWKLTLVILAVSPVLGLSAALWAKVLSAFTDKEQAAYAKAGAVAEEVLAAIRTVIAFGGQEKEIKRYHKNLE 294**

**XP_030922622.1 215 GFIVGLIRGWKLTLVILAVSPVLGLSAALWAKVLSAFTDKEQAAYAKAGAVAEEVLAAIRTVIAFGGQEKEIKRYHKNLE 294**

**XP_030826393.1 215 GFIVGLIRGWKLTLVILAVSPVLGLSAALWAKVLSAFTDKEQAAYAKAGAVAEEVLAAIRTVIAFGGQEKEIKRYHKNLE 294**

**XP_062350659.1 209 GFIVGLIRGWKLTLVILAVSPVLGLSAALWAKVLSAFTDKEQAAYAKAGAVAEEVLGAIRTVIAFGGQEKEIKRYHKNLE 288**

**XP_014731144.1 211 GFIVGLIRGWKLTLVILAVSPVLGLSAALWAKVLSAFTDKEQAAYAKAGAVAEEVLGAIRTVIAFGGQEKEIKRYHKNLE 290**

**XP_032926696.1 216 GFVVGLIRGWKLTLVILAVSPVLGLSAALWAKVLSAFTDKEQAAYAKAGAVAEEVLGAIRTVIAFGGQEKEIKRYHKNLE 295**

**XP_005518835.2 210 GFIVGLIRGWKLTLVILAVSPVLGLSAALWAKVLSAFTDKEQAAYAKAGAVAEEVLAAIRTVIAFGGQEKEIKRYHKNLE 289**

**XP_058687902.1 211 GFVVGLIRGWKLTLVILAVSPVLGLSAALWAKVLSAFTDKEQAAYAKAGAVAEEVLAAIRTVIAFGGQEKEIKRYHKNLE 290**

**XP_015471143.1 211 GFIVGLIRGWKLTLVILAVSPVLGLSAALWAKVLSAFTDKEQAAYAKAGAVAEEVLAAIRTVIAFGGQEKEIKRYHKNLE 290**

**XP_023776043.1 211 GFIVGLIRGWKLTLVILAVSPVLGLSAALWAKVLSAFTDKEQAAYAKAGAVAEEVLAAIRTVIAFGGQEKEIKRYHKNLE 290**

**XP_058275891.1 207 GFIVGLIRGWKLTLVILAVSPVLGLSAALWAKVLSAFTDKEQAAYAKAGAVAEEVLAAVRTVIAFGGQEKEIKRYHQNLE 286**

**XP_063268876.1 212 GFIVGLIRGWKLTLVILAVSPVLGLSAALWAKVLSAFTDKEQAAYAKAGAVAEEVLAAIRTVIAFGGQEKEIKRYHKNLE 291**

**XP_068862854.1 211 GFIVGLIRGWKLTLVILAVSPVLGLSAALWAKVLSAFTDKEQAAYAKAGAVAEEVLAAIRTVIAFGGQEKEIKRYHKNLE 290**

**XP_031968992.1 211 GFIVGLIRGWKLTLVILAVSPVLGLSAALWAKVLSAFTDKEQAAYAKAGAVAEEVLAAIRTVIAFGGQEKEIKRYHKNLE 290**

**XP_010397518.2 211 GFIVGLIRGWKLTLVILAVSPVLGLSAALWAKVLSAFTDKEQAAYAKAGAVAEEVLAAIRTVIAFGGQEKEIKRYHKNLE 290**

**XP_008628111.1 211 GFIVGLIRGWKLTLVILAVSPVLGLSAALWAKVLSAFTDKEQAAYAKAGAVAEEVLAAIRTVIAFGGQEKEIKRYHKNLE 290**

**XP_041899696.1 211 GFIVGLIRGWKLTLVILAVSPVLGLSAALWAKVLSAFTDKEQAAYAKAGAVAEEVLAAIRTVIAFGGQEKEIKRYHKNLE 290**

**XP_048162577.1 211 GFIVGLIRGWKLTLVILAVSPVLGLSAALWAKVLSAFTDKEQAAYAKAGAVAEEVLAAIRTVIAFGGQEKEIKRYHKNLE 290**

**XP_066172927.1 222 GFVVGLIRGWKLTLVILAVSPVLGLSAALWAKVLSAFTDKEQAAYAKAGAVAEEVLAAIRTVIAFGGQEKEIKRYHKNLE 301**

**XP_066054226.1 229 GFVVGLIRGWKLTLVILAVSPVLGLSAALWAKVLSAFTDKEQAAYAKAGAVAEEVLAAIRTVIAFGGQEKEIKRYHKNLE 308**

**XP_057239101.1 210 GFVVGLIRGWKLTLVILAVSPVLGLSAALWAKVLSAFTDKEQAAYAKAGAVAEEVLAAIRTVIAFGGQEKEIKRYHKNLE 289**

**XP_039235593.1 211 GFIVGLVRGWKLTLVILAVSPVLGLSAAIWAKVLSAFTDKEQAAYAKAGAVAEEVLAAVRTVIAFGGQEKEIKRYHKNLE 290**

**XP_027564162.1 212 GFIVGLIRGWKLTLVILAVSPVLGLSAALWAKVLSAFTDKEQAAYAKAGAVAEEVLAAIRTVIAFGGQEKEIKRYHKNLE 291**

**XP_051658500.1 211 GFIVGLIRGWKLTLVILAVSPVLGLSAALWAKVLSAFTDKEQAAYAKAGAVAEEVLAAIRTVIAFGGQEKEIKRYHKNLE 290**

**XP_064534663.1 211 GFIVGLIRGWKLTLVILAVSPVLGLSAAIWAKVLSAFTDKEQAAYAKAGAVAEEVLAAIRTVIAFGGQEKEIKRYHKNLE 290**

**CDM63410.1 290 DAKRIGIRKAITANISMGAAFLLIYASYALAFWYGTTLILTDDYTIGKVLTVFFSVLIGAFSIGQTAPSIEAFASARGAA 369**

**XP_059698659.1 291 DAKRIGIRKAITANISMGAAFLLIYASYALAFWYGTTLILTDDYTIGKVLTVFFSVLIGAFSIGQTAPSIEAFASARGAA 370**

**XP_050827218.1 291 DAKRIGIRKAITANISMGAAFLLIYASYALAFWYGTTLILNDDYTIGKVLTVFFSVLIGAFSIGQTAPSIEAFASARGAA 370**

**XP_037984436.1 290 DAKRIGIRKAITANISMGAAFLLIYASYALAFWYGTTLILSDDYTIGKVLTVFFSVLIGAFSIGQTAPSIEAFASARGAA 369**

**XP_041322342.1 290 DAKRIGIRKAITANISMGAAFLLIYASYALAFWYGTTLILSDDYTIGKVLTVFFSVLIGAFSIGQTAPSIEAFASARGAA 369**

**XP_041264549.1 291 DAKRIGIRKAITANISMGAAFLLIYASYALAFWYGTTLILTDDYTIGKVLTVFFSVLIGAFSIGQTAPSIEAFASARGAA 370**

**XP_039567137.1 291 DAKRIGIRKAITANISMGAAFLLIYASYALAFWYGTTLILNDDYTIGKVLTVFFSVLIGAFSIGQTAPSIEAFASARGAA 370**

**XP_064265165.1 291 DAKRIGIRKAITANISMGAAFLLIYASYALAFWYGTTLILTDDYTIGKVLTVFFSVLIGAFSIGQTAPSIEAFASARGAA 370**

**XP_016151870.1 291 DAKRIGIRKSITANISMGAAFLLIYASYALAFWYGTTLILNDDYTIGKVLTVFFSVLIGAFSIGQTAPSIEAFASARGAA 370**

**XP_058670934.1 295 DAKRMGIRKAITANISMGAAFLLIYASYALAFWYGTTLVLTDDYTIGKVLTVFFSVLIGAFSIGQTAPSIEAFASARGAA 374**

**XP_059323184.1 295 DAKRMGIRKAITANISMGAAFLLIYASYALAFWYGTTLVLTDDYTIGKVLTVFFSVLIGAFSIGQTAPSIEAFASARGAA 374**

**XP_057876310.1 295 DAKRMGIRKAITANISMGAAFLLIYASYALAFWYGTTLVLTDDYTIGKVLTVFFSVLIGAFSIGQTAPSIEAFATARGAA 374**

**XP_063006839.1 295 DAKRMGIRKAITANISMGAAFLLIYASYALAFWYGTTLVLTDDYTIGKVLTVFFSVLIGAFSIGQTAPSIEAFATARGAA 374**

**XP_053808094.1 290 DAKRIGIRKAITANISMGAAFLLIYASYALAFWYGTTLILTDDYTIGKVLTVFFSVLIGAFSIGQTAPSIEAFASARGAA 369**

**XP_068068048.1 289 DAKRIGIRKAITANISMGAAFLLIYASYALAFWYGTTLILTDDYTIGKVLTVFFSVLIGAFSIGQTAPSIEAFASARGAA 368**

**XP_004186266.5 291 DAKRIGIRKSITANISMGAAFLLIYASYALAFWYGTTLILTDDYTIGKVLTVFFSVLIGAFSIGQTAPSIEAFASARGAA 370**

**XP_021407324.2 291 DAKRIGIRKAITANISMGAAFLLIYASYALAFWYGTTLILTDDYTIGKVLTVFFSVLIGAFSIGQTAPSIEAFASARGAA 370**

**XP_054129694.1 295 DAKRMGIRKAITANISMGAAFLLIYASYALAFWYGTTLVLTDDYTIGKVLTVFFSVLIGAFSIGQTAPSIEAFASARGAA 374**

**XP_064560295.1 298 DAKRMGIRKAITANISMGAAFLLIYASYALAFWYGTTLVLTDDYTIGKVLTVFFSVLIGAFSIGQTAPSIEAFASARGAA 377**

**XP_074391572.1 298 DAKRMGIRKAITANISMGAAFLLIYASYALAFWYGTTLVLTDDYTIGKVLTVFFSVLIGAFSIGQTAPSIEAFASARGAA 377**

**XP_056339284.1 291 DAKRIGIRKSITANISMGAAFLLIYASYALAFWYGTTLILNDDYTIGKVLTVFFSVLIGAFSIGQTAPSIEAFASARGAA 370**

**XP_036262392.1 295 DAKRIGIRKAITANISMGAAFLLIYASYALAFWYGTTLILTDDYTIGKVLTVFFSVLIGAFSIGQTAPSIEAFASARGAA 374**

**XP_066408995.1 295 DAKRIGIRKAITANISMGAAFLLIYASYALAFWYGTTLILTDDYTIGKVLTVFFSVLIGAFSIGQTAPSIEAFASARGAA 374**

**XP_054484009.2 295 DAKRIGIRKAITANISMGAAFLLIYASYALAFWYGTTLILTDDYTIGKVLTVFFSVLIGAFSIGQTAPSIEAFASARGAA 374**

**XP_071285034.1 295 DAKRIGIRKAITANISMGVAFLLIYASYALAFWYGTTLILTDDYTIGKVLTVFFSVLIGAFSIGQTAPSIEAFASARGAA 374**

**XP_030922622.1 295 DAKRIGIRKAITANISMGAAFLLIYASYALAFWYGTTLILTDDYTIGKVLTVFFSVLIGAFSIGQTAPSIEAFASARGAA 374**

**XP_030826393.1 295 DAKRIGIRKAITANISMGAAFLLIYASYALAFWYGTTLILTDDYTIGKVLTVFFSVLIGAFSIGQTAPSIEAFASARGAA 374**

**XP_062350659.1 289 DAKRIGIRKAITANISMGAAFLLIYASYALAFWYGTTLILNDDYTIGKVLTVFFSVLIGAFSIGQTAPSIEAFASARGAA 368**

**XP_014731144.1 291 DAKRIGIRKAITSNISMGAAFLLIYASYALAFWYGTTLILTEDYTIGKVLTVFFSVLIGAFSIGQTAPSIEAFASARGAA 370**

**XP_032926696.1 296 DAKRIGIRKAITANISMGAAFFLIYASYALAFWYGTTLILKDDYTIGKVLTVFFSVLIGAFSIGQTAPSIEAFASARGAA 375**

**XP_005518835.2 290 DAKRIGIRKAITANISMGAAFLLIYASYALAFWYGTTLILTDDYTIGKVLTVFFSVLIGAFSIGQTAPSIEAFASARGAA 369**

**XP_058687902.1 291 DAKRIGIRKAITANISMGAAFLLIYASYALAFWYGTTLILTDDYTIGKVLTVFFSVLIGAFSIGQTAPSIEAFASARGAA 370**

**XP_015471143.1 291 DAKRIGIRKAITANISMGAAFLLIYASYALAFWYGTTLILTDDYTIGKVLTVFFSVLIGAFSIGQTAPSIEAFASARGAA 370**

**XP_023776043.1 291 DAKRIGIRKAITANISMGAAFLLIYASYALAFWYGTTLILTDDYTIGKVLTVFFSVLIGAFSIGQTAPSIEAFASARGAA 370**

**XP_058275891.1 287 DAKRIGIRKAITANISMGAAFLLIYASYALAFWYGTTLILSDEYTIGKVLTVFFSVLIGAFSIGQTAPSIEAFASARGAA 366**

**XP_063268876.1 292 DAKRIGIRKAITANISMGAAFLLIYASYALAFWYGTTLVLTDDYTIGKVLTVFFSVLIGAFSIGQTAPSIEAFASARGAA 371**

**XP_068862854.1 291 DAKRIGIRKAITANISMGAAFLLIYASYALAFWYGTTLILTDDYTIGKVLTVFFSVLIGAFSIGQTAPSIEAFASARGAA 370**

**XP_031968992.1 291 DAKRIGIRKAITANISMGAAFLLIYASYALAFWYGTTLILTEDYTIGKVLTVFFSVLIGAFSIGQTAPSIEAFASARGAA 370**

**XP_010397518.2 291 DAKRIGIRKAITANISMGAAFLLIYASYALAFWYGTTLILTEDYTIGKVLTVFFSVLIGAFSIGQTAPSIEAFASARGAA 370**

**XP_008628111.1 291 DAKRIGIRKAITANISMGAAFLLIYASYALAFWYGTTLILTEDYTIGKVLTVFFSILIGAFSIGQTAPSIEAFASARGAA 370**

**XP_041899696.1 291 DAKRIGIRKAITANISMGAAFLLIYASYALAFWYGTTLILTEDYTIGKVLTVFFSVLIGAFSIGQTAPSIEAFASARGAA 370**

**XP_048162577.1 291 DAKRIGIRKAITANISMGAAFLLIYASYALAFWYGTTLILTEDYTIGKVLTVFFSVLIGAFSIGQTAPSIEAFASARGAA 370**

**XP_066172927.1 302 EAKRIGIKKSITANISMGAAFLLIYASYALAFWYGTTLILADDYTIGKVLTVFFSVLIGAFSIGQTAPSIEAFASARGAA 381**

**XP_066054226.1 309 DAKRIGIRKAITANISMGAAFLLIYASYALAFWYGTTLILTDDYTIGKVLTVFFSVLIGAFSIGQTAPSIEAFASARGAA 388**

**XP_057239101.1 290 DAKRIGIKKSITANISMGAAFLLIYASYALAFWYGTTLILSDDYTIGKVLTVFFSVLIGAFSIGQTAPSIEAFASARGAA 369**

**XP_039235593.1 291 DAKRIGIRKAITANISMGAAFLLIYASYALAFWYGTTLVLNDDYTIGKVLTVFFSVLIGAFSIGQTAPSIEAFASARGAA 370**

**XP_027564162.1 292 DAKRIGIRKAITANISMGAAFLLIYASYALAFWYGTTLILTDDYTIGKVLTVFFSVLIGAFSIGQTAPSIEAFASARGAA 371**

**XP_051658500.1 291 DAKRIGIRKAITANISMGAAFLLIYASYALAFWYGTTLVLTDDYTIGKVLTVFFSVLIGAFSIGQTAPSIEAFASARGAA 370**

**XP_064534663.1 291 DAKRIGIRKAITANISMGAAFLLIYASYALAFWYGTTLVLNDDYTIGKVLTVFFSVLIGAFSIGQTAPSIEAFASARGAA 370**

**CDM63417.1 1 --IFNIIDNEPQIDSYSEAGYKPDHIKGNLELKNVYFNYPSRPDVEILKGLNLKINSGQTVALVGGSGCGKSTTVQLIQR 78**

**CDM63410.1 370 YTIFNIIDNEPQIDSYSEAGYKPDHIKGNLELNNVYFNYPSRPDVEILKGLNLKIDSGQTVALVGGSGCGKSTTVQLIQR 449**

**XP_059698659.1 371 YTIFNIIDNEPQIDSYSEAGYKPDHIKGNLELNNVYFNYPSRPDVEILKGLNLKIDSGQTVALVGGSGCGKSTTVQLIQR 450**

**XP_050827218.1 371 YTIFNIIDNEPQIDSYSEAGYKPDHIKGNLELNNVYFNYPSRPDVEILKGLNLKIDSGQTVALVGGSGCGKSTTVQLIQR 450**

**XP_037984436.1 370 YTIFNIIDNEPQIDSYSETGYKPDHIKGNLELKDVYFSYPSRPDVEILKGLNLKINSGQTVALVGGSGCGKSTTVQLIQR 449**

**XP_041322342.1 370 YAIFNIIDNEPQIDSYSETGYKPDHIKGNLELKNVYFNYPSRPDVEILKGLNLKINSGQTVALVGGSGCGKSTTVQLIQR 449**

**XP_041264549.1 371 YAIFNIIDNEPQIDSYSETGYKPDHIKGNLELKNVYFNYPSRPDVEILKGLNLKINSGQTVALVGGSGCGKSTTVQLIQR 450**

**XP_039567137.1 371 YTIFNIIDNEPQIDSYSETGYKPDHIKGNLELKNVYFNYPSRPDVEILKGLNLKINSGQTVALVGGSGCGKSTTVQLIQR 450**

**XP_064265165.1 371 YTIFNIIDNEPQIDSYSETGYKPDHIKGNLELKNVYFNYPSRPDVEILKGLNLKINSGQTVALVGGSGCGKSTTVQLIQR 450**

**XP_016151870.1 371 YTVFNIIDTEPQIDSYSEAGYKPDHIKGNLEFTNVYFNYPSRPDVEILKGLNLKINSGQTVALVGGSGCGKSTTVQLIQR 450**

**XP_058670934.1 375 YAIFNIIDNEPQIDSYSEAGYKPDHIKGNLELINVYFNYPSRPDVEILKGLNLKINSGQTVALVGGSGCGKSTTVQLIQR 454**

**XP_059323184.1 375 YAIFNIIDNEPQIDSYSEAGYKPDHIKGNLELINVYFNYPSRPDVEILKGLNLKINSGQTVALVGGSGCGKSTTVQLIQR 454**

**XP_057876310.1 375 YAIFNIIDNEPQIDSYSEAGYKPDHIKGNLELINVYFNYPSRPDVEILKGLNLKINSGQTVALVGGSGCGKSTTVQLVQR 454**

**XP_063006839.1 375 YAIFNIIDNEPQIDSYSEAGYKPDHIKGNLELINVYFNYPSRPDVEILKGLNLKINSGQTVALVGGSGCGKSTTVQLIQR 454**

**XP_053808094.1 370 YTIFNIIDNEPQIDSYSETGYKPDHIKGNLEFKNVYFNYPSRPDVEILKGLNLKINSGQTVALVGGSGCGKSTTVQLIQR 449**

**XP_068068048.1 369 YTIFNIIDNEPQIDSYSETGYKPDHIKGNLELKNVYFNYPSRPHVEILKGLNLKINSGQTVALVGGSGCGKSTTVQLIQR 448**

**XP_004186266.5 371 YTIFNIIDNEPQIDSYSETGYKPDHIKGNLELKNVYFNYPSRPDVEILKGLNLKINSGQTVALVGGSGCGKSTTVQLIQR 450**

**XP_021407324.2 371 YTIFNIIDNEPQIDSYSETGYKPDHIKGNLELKNVYFNYPSRPDVEILKGLNLKIDSGQTVALVGGSGCGKSTTVQLIQR 450**

**XP_054129694.1 375 YAIFNIIDNEPQIDSYSEAGYKPDHIKGNLELINVYFNYPSRPDVEILKGLNLKINSGQTVALVGGSGCGKSTTVQLIQR 454**

**XP_064560295.1 378 YTIFNIIDNEPQIDSYSEAGYKPDHIKGNLELINVYFNYPSRPDVEILKGLNLKINSGQTVALVGGSGCGKSTTVQLIQR 457**

**XP_074391572.1 378 YAIFNIIDNEPQIDSYSEAGYKPDHIKGNLELINVYFNYPSRPDVEILKGLNLKINSGQTVALVGGSGCGKSTTVQLIQR 457**

**XP_056339284.1 371 YTVFNIIDNEPQIDSYSEAGYKPDHIKGNLEFTNVYFNYPSRPDVEILKGLNLKINSGQTVALVGGSGCGKSTTVQLIQR 450**

**XP_036262392.1 375 YTIFNIIDNEPQIDSYSEAGYKPDHIKGNLELINVYFNYPSRPDVEILKGLNLKINSGQTVALVGGSGCGKSTTVQLIQR 454**

**XP_066408995.1 375 YTIFNIIDNEPQIDSYSEAGYKPDHIKGNLELINVYFNYPSRPDVEILKGLNLKINSGQTVALVGGSGCGKSTTVQLIQR 454**

**XP_054484009.2 375 YTIFNIIDNEPQIDSYSEAGYKPDHIKGNLELINVYFNYPSRPEVEILKGLNLKINSGQTVALVGGSGCGKSTTVQLIQR 454**

**XP_071285034.1 375 YTIFNIIDNEPQIDSYSEAGYKPDHIKGNLELINVYFNYPSRPEVEILKGLNLKINSGQTVALVGGSGCGKSTTVQLIQR 454**

**XP_030922622.1 375 YAIFNIIDNEPQIDSYSEAGYKPDHIQGNLELINVYFNYPSRPDVEILKGLNLKINSGQTVALVGGSGCGKSTTVQLIQR 454**

**XP_030826393.1 375 YAIFNIIDNEPQIDSYSEAGYKPDHIKGNLELINVYFNYPSRPDVEILKGLNLKINSGQTVALVGGSGCGKSTTVQLIQR 454**

**XP_062350659.1 369 YTIFNIIDNEPQIDSYSEAGYKPDHIKGNLEFKNVYFNYPSRPDVEILKGLNLKINSGQTVALVGGSGCGKSTTVQLIQR 448**

**XP_014731144.1 371 YTIFNIIDNEPQIDSYSETGYKPDHIKGNVEFTNVYFNYPSRPDVEILKGLNLKINSGQTVALVGGSGCGKSTTIQLIQR 450**

**XP_032926696.1 376 YMIFNIIDNEPQIDSYSEAGYKPDHIKGNLEFRNVYFNYPSRPDVEILKGLNLKINSGQTVALVGGSGCGKSTTVQLIQR 455**

**XP_005518835.2 370 YTIFNIIDNEPQIDSYSETGYKPDHIKGNLEFKNVYFNYPSRPDVEILKGLNLKINSGQTVALVGGSGCGKSTTVQLIQR 449**

**XP_058687902.1 371 YTVFNIIDNEPQIDSYSETGYKPDHIKGNLEFKNVYFNYPSRPDVEILKGLNLKINSGQTVALVGGSGCGKSTTVQLIQR 450**

**XP_015471143.1 371 YTIFNIIDNEPQIDSYSETGYKPDHIKGNLEFKNVYFNYPSRPDVEILKGLNLKINSGQTVALVGGSGCGKSTTVQLIQR 450**

**XP_023776043.1 371 YTVFNIIDNEPQIDSYSETGYKPDHIKGNLEFKNVYFNYPSRPDVEILKGLNLKINSGQTVALVGGSGCGKSTTVQLIQR 450**

**XP_058275891.1 367 YTVFNIIDNEPQIDSYSETGYKPDHIKGNLEFKNVYFNYPSRPDVEILKGLNLKINSGQTVALVGGSGCGKSTTVQLIQR 446**

**XP_063268876.1 372 YTIFNIIDNEPQIDSYSETGYKPDHIKGNLEFKNVYFNYPSRPDVEILKGLNLKINSGQTVALVGGSGCGKSTTVQLIQR 451**

**XP_068862854.1 371 YTIFNIIDNEPQIDSYSETGYKPDHIKGNLEFKNVYFNYPSRPDVEILKGLNLKINSGQTVALVGGSGCGKSTTVQLIQR 450**

**XP_031968992.1 371 YTVFNIIDNEPQIDSYSETGYKPDHIKGNLEFKNVYFNYPSRPDVEILKGLNLKINSGQTVALVGGSGCGKSTTVQLIQR 450**

**XP_010397518.2 371 YTVFNIIDNEPQIDSYSETGYKPDHIKGNLEFKNVYFNYPSRPDVEILKGLNLKINSGQTVALVGGSGCGKSTTVQLIQR 450**

**XP_008628111.1 371 YTVFNIIDNEPQIDSYSETGYKPDHIKGNLEFKNVYFNYPSRPDVEILKGLNLKINSGQTVALVGGSGCGKSTTVQLIQR 450**

**XP_041899696.1 371 YTVFNIIDNEPQIDSYSETGYKPDHIKGNLEFKNVYFNYPSRPDVEILKGLNLKINSGQTVALVGGSGCGKSTTVQLIQR 450**

**XP_048162577.1 371 YTVFNIIDNEPQIDSYSETGYKPDHIKGNLEFKNVYFNYPSRPDVEILKGLNLKINSGQTVALVGGSGCGKSTTVQLIQR 450**

**XP_066172927.1 382 YTIFNIIDNEPQIDSYSETGHKPDHIKGNLEFKNVYFNYPSRPDVEILKGLNLKINSGQTVALVGGSGCGKSTTVQLIQR 461**

**XP_066054226.1 389 YTVFNIIDNEPQIDSYSETGHKPDHIKGNLEFKDVYFNYPSRPDVEILKGLNLKINSGQTVALVGGSGCGKSTTVQLIQR 468**

**XP_057239101.1 370 YVIFNIIDNEPQIDSYSETGYKPDHMKGNVEFKNVYFNYPSRQDVEILKGLNLKISSGQTVALVGSSGCGKSTTVQLIQR 449**

**XP_039235593.1 371 YVIFNIIDNEPQIDSYSEAGYKPDYIKGNLEFENVYFNYPSRPDVEILKGLNLKISSGQTVALVGSSGCGKSTTVQLIQR 450**

**XP_027564162.1 372 YVIFNIIDNEPQIDSYSEVGYKPDYIKGNLEFKNVYFNYPSRPDVEILKGLNLKISSGQTVALVGSSGCGKSTTVQLIQR 451**

**XP_051658500.1 371 YVIFNIIDNEPQIDSYSEAGYKPDYIKGNLEFENVYFNYPSRPDVEILKGLNLKISSGQTVALVGSSGCGKSTTVQLIQR 450**

**XP_064534663.1 371 YVIFNIIDNEPQIDSYSEAGYKPDYIKGNLEFENVYFNYPSRPDVEILKGLNLKISSGQTVALVGSSGCGKSTTVQLIQR 450**

**CDM63417.1 79 FYDPKEGTVTLDGQDIKTLNIRYLREIIGVVNQEPVLFATTIAENIRYGREDVTMEEIEEATKEANAYDFIMKLPNKFET 158**

**CDM63410.1 450 FYDPKEGMVTIDGQDIKTLNIRYLREVIGVVNQEPVLFATTIAENIRYGREDVTMQEIEKATKEANAYDFIMKLPNKFET 529**

**XP_059698659.1 451 FYDPKEGTVTIDGQDIKTLNIRYLREVIGVVNQEPVLFATTIAENIRYGREDVTMEEIEKATKEANAYDFIMKLPKKFET 530**

**XP_050827218.1 451 FYDPKEGTVTIDGQDIKTLNIRYLREVIGVVNQEPVLFATTIAENIRYGREDVTMEEIEKATKEANAYDFIMKLPKKFET 530**

**XP_037984436.1 450 FYDPKEGTVIIDGQDIKTLNVRYLREVIGVVNQEPVLFATTIAENIRYGREDVTMEEIEKATKEANAYDFIMKLPNKFET 529**

**XP_041322342.1 450 FYDPKEGTVTIDGQDIKTLNVRYLREVIGVVNQEPVLFATTIAENIRYGREDVTMEEIEKATKEANAYDFIMKLPNKFET 529**

**XP_041264549.1 451 FYDPKEGTVTIDGQDIKTLNVRYLREVIGVVNQEPVLFATTIAENIRYGREDVTMEEIEKATKEANAYDFIMKLPNKFET 530**

**XP_039567137.1 451 FYDPKEGTVTIDGQDIKTLNVRYLREVIGVVNQEPVLFATSIAENIRYGREDVTMEEIEKATKEANAYDFIMKLPNKFET 530**

**XP_064265165.1 451 FYDPKEGTVTIDGQDIKTLNVRYLREVIGVVNQEPVLFATSIAENIRYGREDVTMEEIEKATKEANAYDFIMKLPNKFET 530**

**XP_016151870.1 451 FYDPKEGTVTIDGQDIKTLNVRYLREVIGVVNQEPVLFATTIAENIRYGREDVTMEEIEKATKEANAYDFIMKLPNKFET 530**

**XP_058670934.1 455 FYDPKKGTVTIDGQDIKSLNIRYLREVIGVVNQEPVLFATTIAENIRYGREDVTMEEIEKATKEANAYDFIMKLPNKFET 534**

**XP_059323184.1 455 FYDPKKGTVTIDGQDIKSLNIRYLREVIGVVNQEPVLFATTIAENIRYGREDVTMEEIEKATKEANAYDFIMKLPNKFET 534**

**XP_057876310.1 455 FYDPKEGTVTIDGQDIKSLNIRYLREVIGVVNQEPVLFATTIAENIRYGREDVTMEEIEKATKEANAYDFIMKLPNKFET 534**

**XP_063006839.1 455 FYDPKEGTVTIDGQDIKSLNIRYLREVIGVVNQEPVLFATTIAENIRYGREDVTMEEIEKATKEANAYDFIMKLPNKFET 534**

**XP_053808094.1 450 FYDPKEGTVTIDGQDIKTLNVRYLREVIGVVNQEPVLFATTIAENIRYGREDVTMEEIEKATKEANAYDFIMKLPNKFET 529**

**XP_068068048.1 449 FYDPKEGMVTIDGQDIKTLNVRYLREVIGVVNQEPVLFATTIAENIRYGREDVTMEEIEKATKEANAYDFIMKLPNKFET 528**

**XP_004186266.5 451 FYDPKEGTITIDGQDIKTLNVRYLREVIGVVNQEPVLFATTIAENIRYGREDVTMEEIEKATKEANAYDFIMKLPNKFET 530**

**XP_021407324.2 451 FYDPKEGMVTIDGQDIKTLNVRYLREVIGVVNQEPVLFATTIAENIRYGREDVTMEEIEKATKEANAYDFIMKLPNKFET 530**

**XP_054129694.1 455 FYDPKEGTVTLDGQDIKSLNIRYLREVIGVVNQEPVLFATTIAENIRYGREDVTMEEIEKATKEANAYDFIMKLPNKFET 534**

**XP_064560295.1 458 FYDPKEGTVTIDGQDIKSLNIRYLREVIGVVNQEPVLFATTIAENIRYGREDVTMEEIEKATKEANAYDFIMKLPNKFET 537**

**XP_074391572.1 458 FYDPKEGTVTIDGQDIKSLNIRYLREVIGVVNQEPVLFATTIAENIRYGREDVTMEEIEKATKEANAYDFIMKLPNKFET 537**

**XP_056339284.1 451 FYDPKEGTVTIDGQDIKSLNVRYLREVIGVVNQEPVLFATTIAENIRYGREDVTMEEIEKATKEANAYDFIMKLPNKFET 530**

**XP_036262392.1 455 FYDPKEGTVTIDGQDIKTLNVRYLREVIGVVNQEPVLFATTIAENIRYGREDVTMEEIEKATKEANAYDFIMKLPNKFET 534**

**XP_066408995.1 455 FYDPKEGTVTIDGQDIKTLNVRYLREVIGVVNQEPVLFATTIAENIRYGREDVTMEEIEKATKEANAYDFIMKLPNKFET 534**

**XP_054484009.2 455 FYDPKEGTVTIDGQDIKTLNVRYLRDVIGVVNQEPVLFATTIAENIRYGREDVTMEEIEKATKEANAYDFIMKLPNKFET 534**

**XP_071285034.1 455 FYDPKEGTVTIDGQDIKTLNVRYLRDIIGVVNQEPVLFATTIAENIRYGREDVTMEEIEKATKEANAYDFIMKLPNKFET 534**

**XP_030922622.1 455 FYDPKEGTITLDGQDIKTLNVRYLREVIGVVNQEPVLFATTIAENIRYGREDVTMEEIEKATREANAYDFIMKLPNKFET 534**

**XP_030826393.1 455 FYDPKEGTITLDGQDIKTLNVRYLREVIGVVNQEPVLFATTIAENIRYGREDVTMEEIEKATREANAYDFIMKLPNKFET 534**

**XP_062350659.1 449 FYDPKEGTVTIDGQDIKTLNVRYLREVIGVVNQEPVLFATSIAENIRYGREDVTMEEIEKATKEANAYDFIMKLPNKFET 528**

**XP_014731144.1 451 FYDPKEGTVTIDGQDIKTLNVRYLREVIGVVNQEPVLFATTIAENIRYGREDVTMEEIEKATKEANAYDFIMKLPNKFET 530**

**XP_032926696.1 456 FYDPKEGMVTLDGQDIKTLNVRYLREIIGVVNQEPVLFATTIAENIRYGREDVTMEEIEKATKEANAYDFIMKLPNKFET 535**

**XP_005518835.2 450 FYDPKEGTVTIDGQDIKTLNVRYLREVIGVVNQEPVLFATTIAENIRYGREDVTMEEIEKATKEANAYDFIMKLPKKFET 529**

**XP_058687902.1 451 FYDPKEGTVTIDGQDIRTLNVRCLREVIGVVNQEPVLFATTIAENIRYGREDVTMEEIEKATKEANAYDFIMKLPKKFET 530**

**XP_015471143.1 451 FYDPKEGTVTIDGQDIKTLNVRYLREVIGVVNQEPVLFATTIAENIRYGREDVTMEEIEKATKEANAYDFIMKLPKKFET 530**

**XP_023776043.1 451 FYDPKEGTVTIDGQDIKTLNVRYLREVIGVVNQEPVLFATTIAENIRYGREDVTMEEIEKATKEANAYDFIMKLPKKFET 530**

**XP_058275891.1 447 FYDPKEGMVTIDGEDIRTLNVRYLREVIGVVNQEPVLFATTIAENIRYGREDVTMEEIEKATKEANAYDFIMKLPNKFET 526**

**XP_063268876.1 452 FYDPKEGMVTIDGQDIRTLNVRYLREVIGVVNQEPVLFATTIAENIRYGRENVTMEEIEKATKEANAYDFIMKLPNKFET 531**

**XP_068862854.1 451 FYDPKEGTVTIDGHDIKTLNIRYLREVIGVVNQEPVLFATTIAENIRYGREDVTMEEIEKATKEANAYDFIMKLPNKFET 530**

**XP_031968992.1 451 FYDPKEGTVTIDGHDIKTLNVRYLREVIGVVNQEPVLFATTIAENIRYGREDVTMEEIEKATKEANAYDFIMKLPNKFET 530**

**XP_010397518.2 451 FYDPKEGTVTIDGHDIKTLNVRYLREVIGVVNQEPVLFATTIAENIRYGREDVTMEEIEKATKEANAYDFIMKLPNKFET 530**

**XP_008628111.1 451 FYDPKEGTVTIDGHDIKTLNVRYLREVIGVVNQEPVLFATTIAENIRYGREDVTMEEIEKATKEANAYDFIMKLPNKFET 530**

**XP_041899696.1 451 FYDPKEGTVTIDGHDIKTLNVRYLREVIGVVNQEPVLFATTIAENIRYGREDVTMEEIEKATKEANAYDFIMKLPNKFET 530**

**XP_048162577.1 451 FYDPKEGTVTIDGHDIKTLNVRYLREVIGVVNQEPVLFATTIAENIRYGREDVTMEEIEKATKEANAYDFIMKLPNKFET 530**

**XP_066172927.1 462 FYDPKEGMVTIDGQDIRTLNVRYLREVIGVVNQEPVLFATTIAENIRYGREDVTMEDIEKATKDANAYDFIMKLPNKFET 541**

**XP_066054226.1 469 FYDPKEGMVTIDGQDIRTLNVRYLREVIGVVNQEPVLFATTIAENIRYGREDVTMEDIEKATKEANAYDFIMKLPSKFET 548**

**XP_057239101.1 450 FYDPREGTVTIDGQDIKTLNIRYLREVIGVVNQEPVLFATTIAENIRYGREDVTMEEIEKATKEANAYDFIMKLPNKFET 529**

**XP_039235593.1 451 FYDPKEGTVTIDGQDIKTLNVRYLREIIGVVNQEPVLFATTIAENIRYGREDVTMEEIEKATKEANAYDFIMKLPNKFET 530**

**XP_027564162.1 452 FYDPKEGTVTIDGQDIKTLNVRYLREIIGVVNQEPVLFATTIAENIRYGREDVTMEEIEKATKEANAYDFIMKLPKKFET 531**

**XP_051658500.1 451 FYDPKEGTVTIDGQDIKTLNVRYLREIIGVVNQEPVLFATTIAENIRYGREDVTMEEIEKATKEANAYDFIMKLPNKFET 530**

**XP_064534663.1 451 FYDPKEGTVTIDGQDIKTLNVRYLREIIGVVNQEPVLFATTIAENIRYGREDVTMEEIEKATKEANAYDFIMKLPNKFET 530**

**CDM63417.1 159 VVGERGAQLSGGQKQRIAIARALVRNPKILLLDEATSALDTESESVVQAALDKAREGRTTVVVAHRLSTVRNADVIAVFE 238**

**CDM63410.1 530 VVGERGAQLSGGQKQRIAIARALVRNPKILLLDEATSALDTESESVVQAALDKAREGRTTVVVAHRLSTVRNADVIAVFE 609**

**XP_059698659.1 531 VVGERGAQLSGGQKQRIAIARALVRNPKILLLDEATSALDTESESVVQAALDKAREGRTTVVVAHRLSTVRNADVIAVFE 610**

**XP_050827218.1 531 VVGERGAQLSGGQKQRIAIARALVRNPKILLLDEATSALDTESESVVQAALDKAREGRTTVVVAHRLSTVRNADVIAVFE 610**

**XP_037984436.1 530 VVGERGAQLSGGQKQRIAIARALVRNPKILLLDEATSALDTESESVVQAALDKAREGRTTVVVAHRLSTVRNADVIAVFE 609**

**XP_041322342.1 530 VVGERGAQLSGGQKQRIAIARALVRNPKILLLDEATSALDTESESVVQAALDKAREGRTTVVVAHRLSTVRNADVIAVFE 609**

**XP_041264549.1 531 VVGERGAQLSGGQKQRIAIARALVRNPKILLLDEATSALDTESESVVQAALDKAREGRTTVVVAHRLSTVRNADVIAVFE 610**

**XP_039567137.1 531 VVGERGAQLSGGQKQRIAIARALVRNPKILLLDEATSALDTESESVVQAALDKAREGRTTVVVAHRLSTVRNADVIAVFE 610**

**XP_064265165.1 531 VVGERGAQLSGGQKQRIAIARALVRNPKILLLDEATSALDTESESVVQAALDKAREGRTTVVVAHRLSTVRNADVIAVFE 610**

**XP_016151870.1 531 VVGDRGAQLSGGQKQRIAIARALVRNPKILLLDEATSALDTESESVVQAALDKAREGRTTVVVAHRLSTVRNADLIAVFE 610**

**XP_058670934.1 535 VVGERGAQLSGGQKQRIAIARALVRNPKILLLDEATSALDTESESIVQAALDKAREGRTTVVVAHRLSTVRNADVIAVFE 614**

**XP_059323184.1 535 VVGERGAQLSGGQKQRIAIARALVRNPKILLLDEATSALDTESESIVQAALDKAREGRTTVVVAHRLSTVRNADVIAVFE 614**

**XP_057876310.1 535 VVGERGAQLSGGQKQRIAIARALVRNPKILLLDEATSALDTESESIVQAALDKAREGRTTVVVAHRLSTVRNADVIAVFE 614**

**XP_063006839.1 535 VVGERGAQLSGGQKQRIAIARALVRNPKILLLDEATSALDTESESIVQAALDKAREGRTTVVVAHRLSTVRNADVIAVFE 614**

**XP_053808094.1 530 VVGERGAQLSGGQKQRIAIARALVRNPKILLLDEATSALDTESESVVQAALDKAREGRTTVVVAHRLSTVRNADVIAVFE 609**

**XP_068068048.1 529 VVGERGAQLSGGQKQRIAIARALVRNPKILLLDEATSALDTESESVVQAALDKAREGRTTVVVAHRLSTVRNADVIAVFE 608**

**XP_004186266.5 531 VVGERGAQLSGGQKQRIAIARALVRNPKILLLDEATSALDTESEAVVQAALDKAREGRTTVVVAHRLSTVRNADVIAVFE 610**

**XP_021407324.2 531 VVGERGAQLSGGQKQRIAIARALVRNPKILLLDEATSALDTESESVVQAALDKAREGRTTVVVAHRLSTVRNADVIAVFE 610**

**XP_054129694.1 535 VVGDRGAQLSGGQKQRIAIARALVRNPKILLLDEATSALDTESESIVQAALDKAREGRTTVVVAHRLSTVRNADVIAVFE 614**

**XP_064560295.1 538 VVGERGAQLSGGQKQRIAIARALVRNPKILLLDEATSALDTESESIVQAALDKAREGRTTVVVAHRLSTVRNADVIAVFE 617**

**XP_074391572.1 538 VVGERGAQLSGGQKQRIAIARALVRNPKILLLDEATSALDTESESIVQAALDKAREGRTTVVVAHRLSTVRNADVIAVFE 617**

**XP_056339284.1 531 VVGDRGAQLSGGQKQRIAIARALVRNPKILLLDEATSALDTESESVVQAALDKAREGRTTVVVAHRLSTVRNADLIAVFE 610**

**XP_036262392.1 535 VVGERGAQLSGGQKQRIAIARALVRNPKILLLDEATSALDTESESVVQAALDKAREGRTTVVVAHRLSTVRNADVIAVFE 614**

**XP_066408995.1 535 VVGERGAQLSGGQKQRIAIARALVRNPKILLLDEATSALDTESESVVQAALDKAREGRTTVVVAHRLSTVRNADVIAVFE 614**

**XP_054484009.2 535 VVGERGAQLSGGQKQRIAIARALVRNPKILLLDEATSALDTESESVVQAALDKAREGRTTVVVAHRLSTVRNADVIAVFE 614**

**XP_071285034.1 535 VVGERGAQLSGGQKQRIAIARALVRNPKILLLDEATSALDTESESVVQAALDKAREGRTTVVVAHRLSTVRNADVIAVFE 614**

**XP_030922622.1 535 VVGERGAQLSGGQKQRIAIARALVRNPKILLLDEATSALDTESESVVQAALDKAREGRTTVVVAHRLSTVRNADVIAVFE 614**

**XP_030826393.1 535 VVGERGAQLSGGQKQRIAIARALVRNPKILLLDEATSALDTESESVVQAALDKAREGRTTVVVAHRLSTVRNADVIAVFE 614**

**XP_062350659.1 529 VVGERGAQLSGGQKQRIAIARALVRNPKILLLDEATSALDTESESVVQAALDKAREGRTTVVVAHRLSTVRNADLIAVFE 608**

**XP_014731144.1 531 VVGERGAQLSGGQKQRIAIARALVRNPKILLLDEATSALDTESESVVQAALDKAREGRTTVVVAHRLSTVRNADLIAVFE 610**

**XP_032926696.1 536 VVGDRGAQLSGGQKQRIAIARALVRNPKILLLDEATSALDTESESVVQAALDKAREGRTTVVVAHRLSTVRNADLIAVFE 615**

**XP_005518835.2 530 VVGERGAQLSGGQKQRIAIARALVRNPKILLLDEATSALDTESESVVQAALDKAREGRTTVVVAHRLSTVRNADLIAVFE 609**

**XP_058687902.1 531 VVGERGAQLSGGQKQRIAIARALVRNPKILLLDEATSALDTESESVVQAALDKAREGRTTVVVAHRLSTVRNADLIAVFE 610**

**XP_015471143.1 531 VVGERGAQLSGGQKQRIAIARALVRNPKILLLDEATSALDTESESVVQAALDKAREGRTTVVVAHRLSTVRNADLIAVFE 610**

**XP_023776043.1 531 VVGERGAQLSGGQKQRIAIARALVRNPKILLLDEATSALDTESESVVQAALDKAREGRTTVVVAHRLSTVRNADLIAVFE 610**

**XP_058275891.1 527 VVGERGAQLSGGQKQRIAIARALVRNPKILLLDEATSALDTESESIVQAALDKAREGRTTVVVAHRLSTVRNADMIAVFE 606**

**XP_063268876.1 532 VVGERGAQLSGGQKQRIAIARALVRNPKILLLDEATSALDTESESIVQAALDKAREGRTTVVVAHRLSTVRNADLIAVFE 611**

**XP_068862854.1 531 VVGERGAQLSGGQKQRIAIARALVRNPKILLLDEATSALDTESESVVQAALDKAREGRTTVVVAHRLSTVRNADLIAVFN 610**

**XP_031968992.1 531 VVGERGAQLSGGQKQRIAIARALVRNPKILLLDEATSALDTESESVVQAALDKAREGRTTVVVAHRLSTVRNADLIAVFN 610**

**XP_010397518.2 531 VVGERGAQLSGGQKQRIAIARALVRNPKILLLDEATSALDTESESVVQAALDKAREGRTTVVVAHRLSTVRNADLIAVFN 610**

**XP_008628111.1 531 VVGERGAQLSGGQKQRIAIARALVRNPKILLLDEATSALDTESESVVQAALDKAREGRTTVVVAHRLSTVRNADLIAVFN 610**

**XP_041899696.1 531 VVGERGAQLSGGQKQRIAIARALVRNPKILLLDEATSALDTESESVVQAALDKAREGRTTVVVAHRLSTVRNADLIAVFN 610**

**XP_048162577.1 531 VVGERGAQLSGGQKQRIAIARALVRNPKILLLDEATSALDTESESVVQAALDKAREGRTTVVVAHRLSTVRNADLIAVFN 610**

**XP_066172927.1 542 VVGERGAQLSGGQKQRIAIARALVRNPKILLLDEATSALDTESESIVQAALDTAREGRTTVVVAHRLSTVRNADQIVVFE 621**

**XP_066054226.1 549 VVGERGAQLSGGQKQRIAIARALVRNPKILLLDEATSALDTESESIVQAALDKAREGRTTVVVAHRLSTVRNADQIAVFE 628**

**XP_057239101.1 530 VVGERGAQLSGGQKQRIAIARALVRNPKILLLDEATSALDTESESIVQAALDKAREGRTTVVVAHRLSTVRNADLIAVFE 609**

**XP_039235593.1 531 VVGERGAQLSGGQKQRIAIARALVRNPKILLLDEATSALDTESESVVQAALDKAREGRTTVVVAHRLSTVRNADHIAVFE 610**

**XP_027564162.1 532 VVGERGAQLSGGQKQRIAIARALVRNPKILLLDEATSALDTESESVVQAALDKAREGRTTVVVAHRLSTVRNADHIAVFE 611**

**XP_051658500.1 531 VVGERGAQLSGGQKQRIAIARALVRNPKILLLDEATSALDTESESVVQAALDKAREGRTTVVVAHRLSTVRNADHIAVFE 610**

**XP_064534663.1 531 VVGERGAQLSGGQKQRIAIARALVRNPKILLLDEATSALDTESESVVQAALDKAREGRTTVVVAHRLSTVRNADHIAVFE 610**

**CDM63417.1 239 GGVITELGNHAELLERKGIYYKLVNMQAIEAEVPPSGNYETVLPSSGNYETVLPSSENYETVLPSSENYENVCSVKNRES 318**

**CDM63410.1 610 GGVVTELGNHAELLERKGIYYKLVNMQAIEAEVPSSENSENVLPSSENYENVLPSSENYE----------NVRSVKNSES 679**

**XP_059698659.1 611 GGVVTELGNHAELLERKGIYYKLVNMQAIEAEVPSSENYENVLPSSENYENVLPPSENYE----------NVRSVKNSDS 680**

**XP_050827218.1 611 GGAVTELGNHTELLEKKGIYYKLVNMQAIEAEVPSSENNENVLPSSENYEHVLPSSENYE----------NVHSVKNSEC 680**

**XP_037984436.1 610 RGVIKELGNHAELLERKGIYYKLVNMQAIEAEVPSSENYEHVLPSSENYENVLPSSENYE----------NVRSVKNSES 679**

**XP_041322342.1 610 GGVITEIGNHAELLERKGIYYKLVNMQAIEAEVPSSENHENVLPSSENYETVLPLSENYE----------NVRSVKNRES 679**

**XP_041264549.1 611 GGVITEIGNHAELLERKGIYYKLVNMQAIEAEVPSSENYENVLPSSENYESVLPLSENYE----------NVCSVKNRES 680**

**XP_039567137.1 611 GGVITEIGNHAELLERKGIYYKLVNMQAIEAEVPSSENYENVLPSSENYKNILPSSENYE----------NVLSVKNSEA 680**

**XP_064265165.1 611 GGVITEIGNHAELLERKGIYYKLVNMQAIEAEVPSSENYEKVLPSSENYENILPSSENYE----------NVLSVKNSEA 680**

**XP_016151870.1 611 GGVIKEQGNHAQLLERKGIYYKLVSMQAIEAEVPSSENYEYVLPSSENYEYVLPSSENYE----------NVRSVKNSES 680**

**XP_058670934.1 615 GGVIKELGNHAELLEKKGIYYKLVNMQSIEAEVPSSENYEYVLASSENYENVLPSSENYE----------NLHSVKSSES 684**

**XP_059323184.1 615 GGVIKELGNHAELLEKKGIYYKLVNMQSIEAEVPSSENYEYVLASSENYENVLPSSENYE----------NLHSVKSSES 684**

**XP_057876310.1 615 GGVIKELGNHAELLEKKGIYYKLVNMQSIETEVPSSENYEYVLASSENYENVLPSSENYE----------NLHSVKSSES 684**

**XP_063006839.1 615 GGVIKELGNHAELLEKKGIYYKLVNMQSIEAEVPSSENYEYVLASSENYENVLPSSENYE----------NLHSVKSSES 684**

**XP_053808094.1 610 GGVITELGNHAKLLEKKGIYYKLVNMQTIEAEVPSSENYEDVLPSSENYE--------------------NVCSVINSEF 669**

**XP_068068048.1 609 GGVITELGNHAKLLEKKGIYYKLVNMQAIEAEVPSSENYENVLPSSENYE--------------------NVHCVKTSEF 668**

**XP_004186266.5 611 GGVITELGNHAKLLEKKGIYYKLVNMQAVEAEVPSSENYENVLPPSENYE--------------------NVYSVKNSEF 670**

**XP_021407324.2 611 GGVITELGNHAKLLEKKGIYYKLVNMQAIEAEVPSSENYENVLPSSENYE--------------------NVYSIKKSEF 670**

**XP_054129694.1 615 GGVIKELGNHAELLEKKGIYYKLVNMQSIEAEVPSSENYENVLPSSENYE--------------------NLHSVKSSES 674**

**XP_064560295.1 618 GGVIKELGNHAELLEKKGIYYKLVNMQSIEAEVPSSENYEYVPASSENYE--------------------NLHSVKSSES 677**

**XP_074391572.1 618 GGVIKELGNHAELLEKKGIYYKLVNMQSIEAEVPSSENYEYVPASSENYE--------------------NLHSVKSSES 677**

**XP_056339284.1 611 GGVIKEQGNHAQLLERKGIYYKLVSMQAIEAEVPSSENYEYVLPSSENYE--------------------NVRSVKNSES 670**

**XP_036262392.1 615 GGVITELGNHAELLKRKGIYYKLVNMQTIEAEVPSSENYENVLPSSENYE--------------------NVRSVKSSES 674**

**XP_066408995.1 615 GGVITELGNHAELLKRKGIYYKLVNMQTIEAEVPSSENYENVLPSSENYE--------------------NVRSVKSSES 674**

**XP_054484009.2 615 GGVITELGNHAELLERKGIYYKLVNMQTIEAEVPSSENYEKVLPSSENYE--------------------NVRSVKSSES 674**

**XP_071285034.1 615 GGVITELGNHAELLERKGIYYKLVNMQTIEAEVPSSENYEKVLPSSENYE--------------------NVHSVKSTES 674**

**XP_030922622.1 615 GGVITELGNHAELLEKKGIYYKLVNMQAIEAEVPSSENYENVLPSSENYE--------------------NVCSVKSSES 674**

**XP_030826393.1 615 GGVITELGNHAELLEKKGIYYKLVNMQAIEAEVPSSENYENVLPSSENYE--------------------NICSVKSSES 674**

**XP_062350659.1 609 GGVITEQGNHAKLLERKGIYYKLVNMQAIEAEVPSSENYEYVLPSSENYE--------------------NVSSVKNRES 668**

**XP_014731144.1 611 GGVIKEQGNHAKLLERKGIYYKLVNMQAIEAEVPSSENYEHVLPSSENYE--------------------NVRSVKNSES 670**

**XP_032926696.1 616 GGVIKEQGNHAKLLETKGIYYKLVNMQTIDAEAPSSENYEYVLPSSENYE--------------------NVRSIKSSES 675**

**XP_005518835.2 610 GGVITEQGNHAKLLERKGIYHKLVNMQAIEGEVPSSENYE------------------------------NVRSVKNNES 659**

**XP_058687902.1 611 GGVITEQGNHAKLLEKKGIYHKLVNMQAIEAEVPSSENDE------------------------------NVRSVKSNES 660**

**XP_015471143.1 611 GGVITEQGNHAKLLERKGIYHKLVNMQAIEGEVPSSENDE------------------------------NVRSVKNNES 660**

**XP_023776043.1 611 GGVITEQGNHAKLLERKGIYHKLVNMQAIEAEVPSSENYE------------------------------NVHSVKNNES 660**

**XP_058275891.1 607 GGVIKEQGNHVKLLERKGIYHKLVNMQAIEAEVPSSENYE------------------------------NARSVKNSEY 656**

**XP_063268876.1 612 GGVITEQGNHAKLLERKGIYHKLVNMQTIEAEVPSSENHD------------------------------NADSVKNSES 661**

**XP_068862854.1 611 GGVITEQGNHAKLLERKGIYHKLVNMQAIEAEVPLSEDDE------------------------------NAHSAKNSGS 660**

**XP_031968992.1 611 GGVITEQGNHAKLLERKGIYHKLVNMQAIEAEVPPSEDDE------------------------------NAHSAKNSGS 660**

**XP_010397518.2 611 GGVITEQGNHAKLLERKGIYHKLVNMQAIEAEVPPSEDDE------------------------------NAHSAKNSGS 660**

**XP_008628111.1 611 GGVITEQGNHAKLLERKGIYHKLVNMQAIEAEVPPSEDDE------------------------------NAHSAKNSGS 660**

**XP_041899696.1 611 GGVITEQGNHAKLLERKGIYHKLVNMQAIEAEVPPSEDDE------------------------------NAHSAKNSGS 660**

**XP_048162577.1 611 GGVITEQGNHAKLLERKGIYHKLVNMQAIEAEVPPSEDDE------------------------------NAHSAKNSGS 660**

**XP_066172927.1 622 GGVIAEQGNHAQLLERKGIYHKLVNMQSIEAEVPSSGNYE------------------------------NVRSVKNSES 671**

**XP_066054226.1 629 GGVIAEQGNHAKLLERKGIYHKLVNMQAIEAEAPSSENYE------------------------------NVRSVKNSES 678**

**XP_057239101.1 610 GGVIKEQGTHAELLERKGIYHKLVNMQAIDAEVPSSENGE------------------------------NAHGILSGES 659**

**XP_039235593.1 611 GGVVAEQGNHLKLLERKGIYYKLVNMQAIEAEVPSSEKDE------------------------------NALSVQKRES 660**

**XP_027564162.1 612 GGVVAEQGNHVKLLERKGIYYKLVNMQAIEGEVPSSEKDE------------------------------NALSVQNRES 661**

**XP_051658500.1 611 GGVVAEQGNHVTLLERKGIYYKLVNMQAIEAEVPSSEKDE------------------------------NALSVQKRES 660**

**XP_064534663.1 611 GGVVAEQGNHVKLLERKGIYYKLVNMQAIEAEVPSSEKDE------------------------------NALSVQKRES 660**

**CDM63417.1 319 EPDSEEPLTRGLRRRSTRRSMKKPGQQNDSPDEEKTSPAEEIPPASFLKIMKLNKTEWPYFVIGTLCAIINGALQPAFAV 398**

**CDM63410.1 680 EPGSEESLTRGLRRRSTRRSMKRPGEQNDSPDEEKSSPAEEIPPASFLKIMKLNKTEWPYFVAGTFCAIINGALQPAFAV 759**

**XP_059698659.1 681 EPGSEEPLTRGLRRRSTRRSMKKPGEQNDSPDEEKTSPAEEIPPASFLKIMKLNKTEWPYFVAGTLCAIINGALQPAFAV 760**

**XP_050827218.1 681 EPGSEEPLTRGLRRRSTRRSMKKPGEQSDSPDEEKASPAEEIPPASFLKIMKLNKTEWPYFVAGTLCAIINGALQPAFAV 760**

**XP_037984436.1 680 GPDSEESLTRGLRRRSTRRSMKKPGGQNDSSDEEKTSPAEEIPPASFLKIMKLNKTEWPYFVAGTLCAIINGALQPAFAV 759**

**XP_041322342.1 680 EPDSEESLTRGLRRRSTRRSMKKPGEQNDSPDEEKTSPAEEIPPASFLKIMKLNKTEWPYFVAGTLCAIINGALQPAFAV 759**

**XP_041264549.1 681 EPDSEESLTRGLRRRSTRRSMKKPGEQNDSPDEEKTSPAEEIPPASFLKIMKLNKTEWPYFVAGTLCAIINGALQPAFAV 760**

**XP_039567137.1 681 EPDSEESLTRGLRRRSTRRSMKKLGEKNDSPDEEKTSPAEEIPPASFLKIMKLNKTEWPYFVAGTLCAIINGALQPAFAV 760**

**XP_064265165.1 681 EPDSEESLTRGLRRRSTRRSMKKPGEQNDSPDEEKTSPAEEMPPASFLKIMKLNKTEWPYFVAGTLCAIINGALQPAFAV 760**

**XP_016151870.1 681 EPEFKEPLTRGLRRRSTRRSMKKPGGQSDSPDEEKASPAEELPPASFMKIMKLNKTEWPYFVAGLLCAIINGALQPAFAI 760**

**XP_058670934.1 685 EPDSEESLTRGLRRRSTRRSMKKPGEQNDSPDEEKSSPAEELPPASFLKIMKLNKTEWPYFVAGTLCAIINGALQPAFSV 764**

**XP_059323184.1 685 EPDSEESLTRGLRRRSTRRSMKKPGEQNDSPDEEKSSPAEELPPASFLKIMKLNKTEWPYFVAGTLCAIINGALQPAFSV 764**

**XP_057876310.1 685 GPDSEESLTRGLRRRSTRRSMKKPREQNDSPDEEKSSPAEELPPASFLKIMKLNKTEWPYFVVGLLCAIINGALQPAFSV 764**

**XP_063006839.1 685 EPDSEEPLTRGLRRRSTRRSMKKPGEQNDSPDEEKSSPAEELPPASFLKIMKLNKTEWPYFVVGILCAIINGALQPAFSV 764**

**XP_053808094.1 670 EPESEESLTRGLRRRSTRRSMKKPGEQNDSPDEEKASPAEEVPPASFLKIMKLNKTEWPYFVAGTLCAIINGALQPAFAV 749**

**XP_068068048.1 669 EPESEESLTRGLRRRSTRRSMKKPGEQNDSPDEEKTSPAEEVPPASFLKIMKLNKTEWPYFVAGTLCAIINGGLQPAFAV 748**

**XP_004186266.5 671 EPESEESLTRGLRRRSTRRSMKKPGEQNYSPDEEKTSPAEELPPASFLKIMKLNKTEWPYFVAGTLCAIINGGLQPAFAV 750**

**XP_021407324.2 671 EPESEESLSRGLRRRSTRRSMKKPGEQNDSPDEEKTSPAEEVPPASFLKIMKLNKTEWPYFVAGTLCAIINGGLQPAFAV 750**

**XP_054129694.1 675 EPDSEESLTRGLRRRSTRRSMKKPGEQNYSPDEEKSSPAEELPPASFLKIMKLNKTEWPYFVAGTLCAIINGALQPAFAV 754**

**XP_064560295.1 678 EPDSEESLTRGLRRRSTRRSMKKPGEQNDSPDEEKSSPAEELPPASFLKIMKLNKTEWPYFVVGTLCAIINGALQPAFAV 757**

**XP_074391572.1 678 EPDSEESLTRGLRRRSTRRSMKKPGEQNDSPDEEKSSPAEELPPASFLKIMKLNKTEWPYFVVGTLCAIINGALQPAFAV 757**

**XP_056339284.1 671 EPEFEESLTRGLRRRSTRRSMRKPGEQSDSPDEEKASPAEELPPASFMKIMKLNKTEWPYFVAGLLCAIINGALQPAFSI 750**

**XP_036262392.1 675 EPDSEESLTRGLRRRSTRRSMKKPGEQNDSPDEEKTSPAEELPPASFLKIMKLNKTEWPYFVAGTVCAIINGALQPAFSV 754**

**XP_066408995.1 675 EPDSEESLTRGLRRQSTRRSMKKPGEQNDSPDEEKTSPAEELPPASFLKIMKLNKTEWPYFVAGTLCAIINGALQPAFSV 754**

**XP_054484009.2 675 EPDSEESLTRGLRRQSTRRSMKKPGEQNDSPDEEKTSPAEELPPASFLKIMKLNKTEWPYFVAGTLCAIINGALQPAFSV 754**

**XP_071285034.1 675 EPDSEESLTRGLRRQSTRRSMKKPGEQNDSPDEEKTSPAEELPPASFLKIMKLNKTEWPYFVAGTLCAIINGALQPAFSV 754**

**XP_030922622.1 675 EPASEESLTRGLRRRSTRRSTKKPGEQNDSPDEEKTSPAEELPPASFLKIMKLNKTEWPYFVVGTLCAIISGALQPAFAV 754**

**XP_030826393.1 675 EPASEESLTRGLRRRSTRRSTKKPGEQNDSPDEEKTSPAEELPPASFLKIMKLNKTEWPYFVVGTLCAIISGALQPAFAV 754**

**XP_062350659.1 669 EPEFEESLTIGLRRRSTRRSMKKPGEQNDRTDEEKTSPDEELPPASFLKIMKLNKTEWPYFVAGLLCAIINGALQPAFAI 748**

**XP_014731144.1 671 ELEFEEPLTRGLRRRSTRRSMKKPGEQNDRLDEEKTSPAEELPPASFMKIMKLNKTEWPYFVAGLLCAIINGALQPAFAV 750**

**XP_032926696.1 676 EPEFEDSLSRGLRRRSTRRSLKKPGEKNDSPDEEKTSPDEELPPASFMKILKLNKTEWPYFVAGLLCAIINGGLQPAFAV 755**

**XP_005518835.2 660 EPESEESLTKGLRRQSTRRSMKKPGEQNDSPDEEKTSPAEELPPASFLKIMKLNKTEWPYFVAGILCAIINGALQPAFAI 739**

**XP_058687902.1 661 EPESEESLTKGLRRQSTRRSMKKPGEQNDSTDEEKTSPAEELPPASFLKIMKLNKTEWPYFVAGILCAIINGALQPAFAI 740**

**XP_015471143.1 661 EPESEESLTKGLRRQSIRRSMKKPGEQNDSPDEEKTSPDEELPPASFLKIMKLNKTEWPYFVAGILCAIINGALQPAFAI 740**

**XP_023776043.1 661 QPESEESLTKGLRRQSTRRSMKKPGEQNDSPDEEKASPAEELPPASFLKIMKLNKTEWPYFVAGIFCAIINGALQPAFAV 740**

**XP_058275891.1 657 EPEPEESLTKGLRRRSTRRSMKKPGEQNESLDEEKTSPAEELPPVSFLKIMKLNKTEWPYFVAGTLCAIINGALQPSFSV 736**

**XP_063268876.1 662 EAEFEESLTKGLRRRSTRRSMKKPGEQNDSPDEEKTSPAEEVPPASFLKIMKLNKTEWPYFVAGTLCAIINGALQPAFAI 741**

**XP_068862854.1 661 EPEFEESLTTGLRRRSTRRSMKKPGEQNNGPDEEETSPAEELRPASFLKIMKLNKTEWPYFVAGTLCAIINGGLQPAFAI 740**

**XP_031968992.1 661 EPEFEESLTTGLRRRSTRRSMKKPGEQNNGPDEEKTSPAEELRPASFLKIMKLNKTEWPYFVAGTLCAIINGGLQPAFAV 740**

**XP_010397518.2 661 EPEFEESLTTGLRRRSTRRSMKKPGEQNNGPDEEKTSPAEELRPASFLKIMKLNKTEWPYFVAGTLCAIINGGLQPAFAV 740**

**XP_008628111.1 661 EPEFEESLTTGLRRRSTRRSMKKPGEQNNGPDEEKTSPAEELRPASFLKIMKLNKTEWPYFVAGTLCAIINGGLQPAFAV 740**

**XP_041899696.1 661 EPEFEESLTTGLRRQSTRRSMKKPGEQNNGPDEEKTSPAEELRPASFLKIMKLNKTEWPYFVAGTLCAIINGGLQPAFAV 740**

**XP_048162577.1 661 EPEFEESLTTGLRRRSTRRSMKKPGEQNNGPDEEKTSPAEELRPASFLKIMKLNKTEWPYFVAGTLCAIINGGLQPAFAV 740**

**XP_066172927.1 672 ELEFEESLTKGLRRRSTRRSMKKPGEQNDNPDEEKTSPAEELPPASFLKIMKLNKXEWPYFVAGTLCAIINGALQPAFAI 751**

**XP_066054226.1 679 DPEFDESLTKGLRRRSTRRSIKKPGEQNNSPDEEKTSPAEELPPASFMKIMKLNKTEWPYFVTGTLCAIINGALQPAFAI 758**

**XP_057239101.1 660 EPEFGES-SKGLRRRSTRRSMKKPGEQNDGTDEEKTSPAEELPPASFLKIMKLNKTEWPYFVGGTLCAIINGALQPAFAV 738**

**XP_039235593.1 661 EPESEESLSRGLRRRSTRRSMKKPGEQDDGPDEKKSSPEEELPPASFMKIMKLNKTEWPYFVAGILCAVINGALQPGFAI 740**

**XP_027564162.1 662 EPESEESLTRGLRRRSTRRSMKKPGEQDDGPDEKKSSPEEELPPASFMKIMKLNKTEWPYFVAGTLCAVINGALQPGFAI 741**

**XP_051658500.1 661 EPEFEESLSSGLRRRSTRRSMKKPGEQDDGPDEKKSSPEEELPPASFMKIMKLNKTEWPYFVAGILCAVINGALQPGFAI 740**

**XP_064534663.1 661 EPESEESLSRGLRRRSTRRSMKKPGEQDDGPDEKKSSPEEELPPASFMKIMKLNKTEWPYFVAGILCAVINGALQPGFAI 740**

**CDM63417.1 399 IFSEIIGIFSETDKDLLRKQSNLYSLLFLVLGIISFFTFFFQ-GFTFGKAGEILTMRLRFMAFKAMLRQDMAWFDNPKNS 477**

**CDM63410.1 760 IFSEIIGIFSETDKDLLRKQSNLYSLLFLVLGIISFFTFFFQ-GFTFGKAGEILTMRLRFMAFKAMLRQDMAWFDNPKNS 838**

**XP_059698659.1 761 IFSEIIGIFSETDKDLLRKQSNLYSLLFLVLGIISFFTFFFQ-GFTFGKAGEILTMRLRFMAFKAMLRQDMAWFDNPKNS 839**

**XP_050827218.1 761 IFSEIIGIFSETDKDLLRKQSNLYSLLFLVLGIISFFTFFFQ-GFAFGKAGEILTMRLRFMAFKAMLRQDMAWFDNPKNS 839**

**XP_037984436.1 760 IFSEIIGIFSETNKDVLRKQSNLYSLLFLVLGIISFFTFFLQ-GFTFGKAGEILTMRLRFMAFKAMLRQDMAWFDNPKNS 838**

**XP_041322342.1 760 IFSEIIGIFSETDKDILRKQSNLYSLLFLVLGIISFFTFFFQ-GFTFGKAGEILTMRLRFMAFKAMLRQDMAWFDNPKNS 838**

**XP_041264549.1 761 IFSEIIGIFSETDKDILRKQSNLYSLLFLVLGIISFFTFFFQ-GFTFGKAGEILTMRLRFMAFKAMLRQDMAWFDNPKNS 839**

**XP_039567137.1 761 IFSEIIGIFSETDKDVLRKQSNLYSLLFLVLGIISFFTFFFQ-GFTFGKAGEILTMRLRFMAFKAMLRQDMAWFDNPKNS 839**

**XP_064265165.1 761 IFSEIIGIFSETDKDVLRKQSNLYSLLFLVLGIISFFTFFFQ-GFTFGKAGEILTMRLRFMAFKAMLRQDMAWFDNPKNS 839**

**XP_016151870.1 761 IFSEIIGIFSETDKDILRKQSNLYSLLFLVLGIISFFTFFFQ-GFTFGKAGEILTMRLRFMAFKAMLRQDMGWFDNPKNS 839**

**XP_058670934.1 765 IFSEIIGIFTETDKELLRKQSNLYSLLFLVLGIISFFTFFFQ-GFAFGKAGEILTMRLRFMAFKAMLRQDMAWFDNPKNS 843**

**XP_059323184.1 765 IFSEIIGIFTETDKELLRKQTNLYSLLFLVLGIISFFTFFFQ-GFAFGKAGEILTMRLRFMAFKAMLRQDMAWFDNPKNS 843**

**XP_057876310.1 765 IFSEIIGIFTETDRELLRKQSNLYSLLFLVLGIISFFTFFFQ-GFAFGKAGEILTMRLRFMAFKAMLRQDMAWFDNPKNS 843**

**XP_063006839.1 765 IFSEIIGIFTETDRELLRKQSNLYSLLFLVLGIISFFTFFFQ-GFAFGKAGEILTMRLRFMAFKAMLRQDMAWFDNPKNS 843**

**XP_053808094.1 750 IFSEIIGIFSETDKNVLRKQSNLYSLLFLVLGIISFFTFFFQ-GFTFGKAGEILTMRLRFMAFKAMLRQDMAWFDNPKNS 828**

**XP_068068048.1 749 IFSEIIGIFSETDKNFLRKQSNLYSLLFLALGIISFFTFFFQ-GFTFGKAGEILTMRLRFMAFKAMLRQDMAWFDNPKNS 827**

**XP_004186266.5 751 IFSEIIGIFSETDKDVLRKQSNLYSLLFLALGIISFFTFFFQ-GFTFGKAGEILTMRLRFMAFKAMLRQDMAWFDNPKNS 829**

**XP_021407324.2 751 IFSEIIGIFSETDKDVLRKQSNLYSLLFLALGIISFFTFFFQ-GFTFGKAGEILTMRLRFMAFKAMLRQDMAWFDNPKNS 829**

**XP_054129694.1 755 IFSEIIGIFTETDKEVLRKQSNLYSLLFLVLGIISFFTFFFQ-GFAFGKAGEILTMRLRFMAFKAMLRQDMAWFDNPKNS 833**

**XP_064560295.1 758 IFSEIIGIFTETDKEALRKQSNLYSLLFLVLGIISFFTFFFQ-GFAFGKAGEILTMRLRFMAFKAMLRQDMAWFDNPKNS 836**

**XP_074391572.1 758 IFSEIIGIFAETDKEALRKQSNLFSLLFLVLGIISFFTFFFQ-GFAFGKAGEILTMRLRFMAFKAMLRQDMAWFDNPKNS 836**

**XP_056339284.1 751 IFSEIIGIFSETDKDFLRKQSNLYSLLFLVLGIISFFTFFFQ-GFTFGKAGEILTMRLRFMAFKAMLRQDMGWFDNPKNS 829**

**XP_036262392.1 755 IFSEIIGIFSETDKTILRKQSNLYSLLFLVLGIISFFTFFFQ-GFTFGKAGEILTMRLRFMAFKAMLRQDMAWFDNPKNS 833**

**XP_066408995.1 755 IFSEIIGIFSETDKTVLRKQSNLYSLLFLVLGIISFFTFFFQ-GFTFGKAGEILTMRLRFMAFKAMLRQDMAWFDNPKNS 833**

**XP_054484009.2 755 IFSEIIGIFSETDKTFLRKQSNLYSLLFLVLGIISFFTFFFQ-GFAFGKAGEILTMRLRFMAFKAMLRQDMAWFDNPKNS 833**

**XP_071285034.1 755 IFSEIIGIFSETDKTFLRKQSNLYSLLFLVLGIISFFTFFFQ-GFAFGKAGEILTMRLRFMAFKAMLRQDMAWFDNPKNS 833**

**XP_030922622.1 755 IFSEIIGIFSETDKDALRKQSNIYSLLFLVVGIISFFTFFFQ-GFAFGKAGEILTMRLRFMAFKAMLRQDMAWFDNPKNS 833**

**XP_030826393.1 755 IFSEIIGIFSETDKDALRKQSNIYSLLFLVVGIISFFTFFFQ-GFAFGKAGEILTMRLRFMAFKAMLRQDMAWFDNPKNS 833**

**XP_062350659.1 749 IFSEIIGIFSESDKVILRKQSNLYSLLFLVLGIISFFTFFLQ-GYTFGKAGEILTMRLRFMAFKAMLRQDMGWFDNPKNT 827**

**XP_014731144.1 751 IFSEIIGIFSETDKTILRKQSNLYSLLFLVLGIISFFTFFFQ-GFTFGKAGEILTMRLRFMAFKAMLRQDMGWFDNPKNS 829**

**XP_032926696.1 756 IFSEIIGIFSTTDKVYLRKQSDLYSLLFLVLGIISFFTFFFQ-GYTFGKAGEILTMRLRFMAFKAMLRQDMGWFDNPKNS 834**

**XP_005518835.2 740 IFSEIIGIFRETDKDILRKQSNLYSLLFLVLGIISFFTFFFQ-GFTFGKAGEILTMRLRFMAFKAMLRQDMGWFDNPKNS 818**

**XP_058687902.1 741 IFSEIIGIFNETDKDILRKQSNLYSLLFLVLGIISFFTFFFQ-GFTFGKAGEILTMRLRFMAFKAMLRQDMGWFDNPKNS 819**

**XP_015471143.1 741 IFSEIIGIFRETDKDILRKQSNLYSLLFLVLGIISFFTFFFQ-GFTFGKAGEILTMRLRFMAFKAMLRQDMGWFDNPKNS 819**

**XP_023776043.1 741 IFSEIIGIFTETDKDILRKQSNLYSLLFLVLGIISFFTFFFQ-GFTFGKAGEILTMRLRFMAFKAMLRQDMGWFDNPKNS 819**

**XP_058275891.1 737 IFSEIIGIFSETDKDILRKKSNLYSLLFLGLGIISFFTFFLQ-GFTFGKAGEILTMRLRFMAFKAMLRQDMGWFDNPKNS 815**

**XP_063268876.1 742 IFSEIIGIFSETDKDLLRKQSNLYSLLFLALGIISFFTFFVQ-GFAFGKAGEILTMRLRFMAFKAMLRQDMGWFDNPKNS 820**

**XP_068862854.1 741 IFSEIIGIFSETDKDILREKSNLYSLLFLVLGIVSFFTFFFQ-GFTFGKAGEILTMRLRFMAFKAMLRQDMGWFDNPKNS 819**

**XP_031968992.1 741 IFSEIIGIFSETDKDILREKSNLYSLLFLVIGIISFFTFFFQ-GFTFGKAGEILTMRLRFMAFKAMLRQDMGWFDNPKNS 819**

**XP_010397518.2 741 IFSEIIGIFSETDKDILREKSNLYSLLFLVIGIISFFTFFFQ-GFTFGKAGEILTMRLRFMAFKAMLRQDMGWFDNPKNS 819**

**XP_008628111.1 741 IFSKIIGIFSETDKDILREKSNLYSLLFLVIGIISFFTFFFQ-GFTFGKAGEILTMRLRFMAFKAMLRQDMGWFDNPKNS 819**

**XP_041899696.1 741 IFSEIIGIFSETDKDILREKSNLYSLLFLVIGIISFFTFFFQ-GFTFGKAGEILTMRLRFMAFKAMLRQDMGWFDNPKNS 819**

**XP_048162577.1 741 IFSEIIGIFSETDKDILREKSNLYSLLFLVIGIISFFTFFFQ-GFTFGKAGEILTMRLRFMAFKAMLRQDMGWFDNPKNS 819**

**XP_066172927.1 752 IFSKIIGIFSETDKDYLRKQSNLYSLLFLVLGIISFLLSFFRYGFAFGKAGEILTMRLRFMAFKAMLRQDMGWFDNPXNS 831**

**XP_066054226.1 759 IFSEIIGIFSETDKDILRKKSNLYSLLFLVLGIISFFTFFFQ-GFTFGKAGEILTMRLRFMAFKAMLRQDMGWFDNPKNS 837**

**XP_057239101.1 739 IFSEIIGIFQETDKDILRQKSNLYSLLFLVLGIISFFTFFFQ-GFSFGKAGEILTMRLRFMAFKAMLRQDMGWFDNPKNS 817**

**XP_039235593.1 741 IFSEIIGVFSETDKDVLREQSNLYSLLFLVLGIISFFTFFFQ-GFTFGKSGEILTMRLRFMAFKAMLRQDMGWFDNPKNS 819**

**XP_027564162.1 742 IFSEIIGVFSETDKDVLRQQSNLYSLLFLVLGIISFFTFFFQ-GFTFGKSGEILTMRLRFMAFKAMLRQDMSWFDDPKNS 820**

**XP_051658500.1 741 IFSEIIGVFSETDKDILRQQSNLYSLLFLVLGIISFFTFFFQ-GFTFGKSGEILTMRLRFMAFKAMLRQDMGWFDNPKNS 819**

**XP_064534663.1 741 IFSEIIGVFSETDKDILREQSNLYSLLFLVLGIISFFTFFFQ-GFTFGKSGEILTMRLRFMAFKAMLRQDMGWFDNPKNS 819**

**CDM63417.1 478 TGALTTRLANDASQVKGATGVRLALIAQNIANLGTGILISLI-------------------------------------- 519**

**CDM63410.1 839 TGALTTRLANDASQVKGATGVRLALIAQNVANLGTGILISLIYGWQLTLLLLAVVPIIAVAGMIEMKMLAGHAKKDKREL 918**

**XP_059698659.1 840 TGALTTRLANDASQVKGATGVRLALIAQNVANLGTGILISLIYGWQLTLLLLAVVPIIAVAGMIEMKMLAGHAKKDKREL 919**

**XP_050827218.1 840 TGALTTRLANDASQVKGATGVRLALIAQNIANLGTGILISLIYGWQLTLLLLAVVPIIAVAGMIEMKMLAGHAKKDKREL 919**

**XP_037984436.1 839 TGALTTRLANDASQVKGATGVRLALIAQNIANLGTGIIISLIYGWQLTLLLLAVVPIIAVAGMIEMKMLAGHAKKDKREL 918**

**XP_041322342.1 839 TGALTTRLANDASQVKGATGVRLALIAQNVANLGTGILISLIYGWQLTLLLLAVVPIIAVAGMIEMKMLAGHAKKDKQEL 918**

**XP_041264549.1 840 TGALTTRLANDASQVKGATGARLALIAQNVANLGTGILISLIYGWQLTLLLLAVVPIIAVAGMIEMKMLAGHAKKDKREL 919**

**XP_039567137.1 840 TGALTTRLANDASQVKGATGVRLALIAQNVANLGTGIIISLIYGWQLTLLLLAVVPIIAVAGMIEMKMLAGHAKKDKREL 919**

**XP_064265165.1 840 TGALTTRLANDASQVKGATGVRLALIAQNVANLGTGIIISLIYGWQLTLLLLAVVPIIAVAGMIEMKMLAGHAKKDKREL 919**

**XP_016151870.1 840 TGALTTRLANDASQVKGATGVRLALIAQNIANLGTGIIISLVYGWQLTLLLLAVVPIIAVAGMIEMKMLAGHAKKDKQEL 919**

**XP_058670934.1 844 TGALTTRLANDASQVKGATGVRLALIAQNIANLGTGIVISLVYGWQLTLLLLAVVPIIAVAGMIEMKMLAGHAKKDKREL 923**

**XP_059323184.1 844 TGALTTRLANDASQVKGATGVRLALIAQNIANLGTGIVISLVYGWQLTLLLLAVVPIIAVAGMIEMKMLAGHAKKDKREL 923**

**XP_057876310.1 844 TGALTTRLANDASQVKGATGVRLALIAQNIANLGTGIVISLVYGWQLTLLLLAVVPIIAVAGMIEMKMLAGHAKKDKREL 923**

**XP_063006839.1 844 TGALTTRLANDASQVKGATGVRLALIAQNIANLGTGIVISLVYGWQLTLLLLAVVPIIAVAGMIEMKMLAGHAKKDKREL 923**

**XP_053808094.1 829 TGALTTRLANDASQVKGATGVRLALIVQNIANLGTGILISLIYGWQLTLLLLAVVPIIAVAGMIEMKMLAGHAKKDKREL 908**

**XP_068068048.1 828 TGALTTRLANDASQVKGATGVRLALIAQNIANLGTGILISLIYGWQLTLLLLAVVPIIAVAGMIEMKMLAGHAKKDKREL 907**

**XP_004186266.5 830 TGALTTRLANDASNVKGATGVRLALIAQNIANLGTGIIISLIYVWKLTLLLLAVVPIIAVAGMIEMKMLAGHAKKDKREL 909**

**XP_021407324.2 830 TGALTTRLANDASNVKGATGVRLALIAQNIANLGTGILISLIYVWKLTLLLLAVVPIIAVAGMIEMKMLAGHAKKDKREL 909**

**XP_054129694.1 834 TGALTTRLANDASQVKGATGVRLALIAQNIANLGTGIVISLVYGWQLTLLLLAVVPIIAVAGMIEMKMLAGHAKKDKREL 913**

**XP_064560295.1 837 TGALTTRLANDASQVKGATGVRLALIAQNIANLGTGIIISLVYGWQLTLLLLAVVPIIAVAGMIEMKMLAGHAKKDKREL 916**

**XP_074391572.1 837 TGALTTRLANDASQVKGATGVRLALIAQNIANLGTGIIISLVYGWQLTLLLLAVVPIIAVAGMIEMKMLAGHAKKDKREL 916**

**XP_056339284.1 830 TGALTTRLANDASQVKGATGVRLALIAQNIANLGTGIIISLVYGWQLTLLLLAVVPIIAVAGMIEMKMLAGHAKKDKQEL 909**

**XP_036262392.1 834 TGALTTRLANDASQVKGATGVRLALIAQNIANLGTGIVISLVYGWQLTLLLLAVVPIIAVAGMIEMKMLAGHAKKDKQEL 913**

**XP_066408995.1 834 TGALTTRLANDASQVKGATGVRLALIAQNIANLGTGIVISLVYGWQLTLLLLAVVPIIAVAGMIEMKMLAGHAKKDKQEL 913**

**XP_054484009.2 834 TGALTTRLANDASQVKGATGVRLALIAQNIANLGTGIVISLVYGWQLTLLLLAVVPIIAVAGMIEMKMLAGHAKKDKREL 913**

**XP_071285034.1 834 TGALTTRLANDASQVKGATGVRLALIAQNIANLGTGIVISLVYGWQLTLLLLAVVPIIAVAGMIEMKMLAGHAKKDKREL 913**

**XP_030922622.1 834 TGALTTRLANDASQVKGATGVRLALIAQNIANLGTGIIISLVYGWQLTLLLLAVVPIIAVAGMIEMKMLAGHAKKDKREL 913**

**XP_030826393.1 834 TGALTTRLANDASQVKGATGVRLALIAQNIANLGTGIIISLVYGWQLTLLLLAVVPIIAVAGMIEMKMLAGHAKKDKREL 913**

**XP_062350659.1 828 TGALTTRLANDASQVKGATGVRLALIAQNIANLGTGIIISLIYGWQLTLLLLAVVPIIAVAGMIEMKMLAGHAKKDKQEL 907**

**XP_014731144.1 830 TGALTTRLANDASQVKGATGVRLALIAQNIANLGTGIIISLIYGWQLTLLLLAVVPIIAVAGMIEMKMLAGHAKKDKQEL 909**

**XP_032926696.1 835 TGALTTRLANDASQVKGATGTRLALIAQNIANLGTGIIISLVYGWQLTLLLLAVVPIIAVAGMIEMKMLAGHAKKDKQEL 914**

**XP_005518835.2 819 TGALTTRLANDASQVKGATGVRLALIAQNIANLGTGIIISLVYGWQLTLLLLAVVPIIAVAGMIEMKMLAGHAKKDKREL 898**

**XP_058687902.1 820 TGALTTRLANDASQVKGATGVRLALIAQNIANLGTGIIISLVYGWQLTLLLLAVVPIIAVAGMIEMKMLAGHAKKDKREL 899**

**XP_015471143.1 820 TGALTTRLANDASQVKGATGVRLALIAQNIANLGTGIIISLVYGWQLTLLLLAVVPIIAVAGMIEMKMLAGHAKKDKREL 899**

**XP_023776043.1 820 TGALTTRLANDASQVKGATGVRLALIAQNIANLGTGIIISLVYGWQLTLLLLAVVPIIAVAGMIEMKMLAGHAKKDKKEL 899**

**XP_058275891.1 816 TGALTTRLANDASQVKGATGVRLALIAQNIANLGTGIIISLVYGWKLTLLLLAVVPIIAVAGMIEMKMLAGHAKKDKREL 895**

**XP_063268876.1 821 TGALTTRLANDASQVKGATGVRLALIAQNIANLGTGIIISLVYGWQLTLLLLAVVPIIAVAGMIEMKMLAGHAKKDKREL 900**

**XP_068862854.1 820 TGALTTRLANDASQVKGATGVRLALIAQNVANLGTGIIISLVYGWQLTLLLLAVVPIIAVAGMIEMKMLAGHAKKDKQEL 899**

**XP_031968992.1 820 TGALTTRLANDASQVKGATGVRLALIAQNIANLGTGIIISLVYGWQLTLLLLAVVPIIAVAGMIEMKMLAGHAKKDKQEL 899**

**XP_010397518.2 820 TGALTTRLANDASQVKGATGVRLALIAQNIANLGTGIIISLVYGWQLTLLLLAVVPIIAVAGMIEMKMLAGHAKKDKQEL 899**

**XP_008628111.1 820 TGALTTRLANDASQVKGATGVRLALIAQNIANLGTGIIISLVYGWQLTLLLLAVVPIIAVAGMIEMKMLAGHAKKDKQEL 899**

**XP_041899696.1 820 TGALTTRLANDASQVKGATGVRLALIAQNIANLGTGIIISLVYGWQLTLLLLAVVPIIAVAGMIEMKMLAGHAKKDKQEL 899**

**XP_048162577.1 820 TGALTTRLANDASQVKGATGVRLALIAQNIANLGTGIIISLVYGWQLTLLLLAVVPIIAVAGMIEMKMLAGHAKKDKQEL 899**

**XP_066172927.1 832 TGALTTRLANDASQVKGATGVRLALIAQNVANLGTGIIISLVYGWQLTLLLLAVVPIIAVAGMIEMKMLAGHAKKDKREL 911**

**XP_066054226.1 838 TGALTTRLANDASQVKGATGVRLALIAQNIANLGTGIIISLVYGWQLTLLLLAVVPIIAVAGMIEMKMLAGHAKKDKREL 917**

**XP_057239101.1 818 TGALTTRLANDASQVKGATGVRLALIAQNIANLGTGIIISLVYGWQLTLLLLAVVPIIAVAGMIEMKMLAGHAKKDKREL 897**

**XP_039235593.1 820 TGALTTRLANDASQVKGATGVRLALIAQNIANLGTGIIISLIYGWQLTLLLLAVVPIIAVAGMIEMKMLAGHAKKDKKEL 899**

**XP_027564162.1 821 TGALTTRLANDASQVKGATGVRLALIAQNIANLGTGIIISLVYGWQLTLLLLAVVPIIAVAGMIEMRMLAGHAKKDKKEL 900**

**XP_051658500.1 820 TGALTTRLANDASQVKGATGVRLALIAQNIANLGTGIIISLIYGWQLTLLLLAVVPIIAVAGMIEMKMLAGHAKKDKKEL 899**

**XP_064534663.1 820 TGALTTRLANDASQVKGATGVRLALIAQNIANLGTGIIISLIYGWQLTLLLLAVVPIIAVAGMIEMKMLAGHAKKDKKEL 899**

**CDM63410.1 919 EAAGKIATEAIENIRTVVSLTRERKFESMYGEHLIVPYRNSVKKAHIFGFCFALSQAMMFFTYAGCFRFGAYLVVNGHME 998**

**XP_059698659.1 920 EAAGKIATEAIENIRTVVSLTRERKFESMYGEHLIVPYRNSVKKAHIFGFCFALSQAMMFFTYAGCFRFGAYLVVNGHME 999**

**XP_050827218.1 920 EAAGKIATEAIENIRTVVSLTRERKFESMYGEHLIVPYRNSVKKAHIFGFCFALSQAMMFFTYAGCFRFGAYLVVNGHME 999**

**XP_037984436.1 919 EAAGKIATEAIENIRTVASLTRERKFESMYGEHLLVPYRNSVKKAHIFGFCFALSQAMMFFTYAGCFRFGAYLVVNGHME 998**

**XP_041322342.1 919 EAAGKIATEAIENIRTVVSLTRERKFESMYGEHLIVPYRNSVKKAHIFGFCFALSQAMMFFTYAGCFRFGAYLVVNGHME 998**

**XP_041264549.1 920 EAAGKIATEAIENIRTVVSLTRERKFESMYGEHLIVPYRNSVKKAHIFGFCFALSQAMMFFTYAGCFRFGAYLVVNGHME 999**

**XP_039567137.1 920 EAAGKIATEAIENIRTVVSLTRERKFESMYGEHLIVPYRNSVKKAHIFGFCFALSQAMMFFTYAGCFRFGAYLVVNGYME 999**

**XP_064265165.1 920 EAAGKIATEAIENIRTVVSLTRERKFESMYGEHLIVPYRNSVKKAHIFGFCFALSQAMMFFTYAGCFRFGAYLVVNGYME 999**

**XP_016151870.1 920 ESAGKIATQAIENIRTVASLTRERKFESMYGEYLVVPYRNSVKKAHIFGFCFALSQAMMFFTYAGCFRFGAYLVVNGHME 999**

**XP_058670934.1 924 EAAGKIATEAIENIRTVVSLTRERKFELMYGEHLIVPYRNSVKKAHIFGFCFALSQAMMFFTYAGCFRFGAYLVVNDLME 1003**

**XP_059323184.1 924 EAAGKIATEAIENIRTVVSLTRERKFELMYGEHLIVPYRNSVKKAHIFGFCFALSQAMMFFTYAGCFRFGAYLVVNDLME 1003**

**XP_057876310.1 924 EAAGKIATEAIENIRTVVSLTRERKFELMYGEHLIVPYRNSVKKAHIFGFCFALSQAMMFFTYAGCFRFGAYLVVNDLME 1003**

**XP_063006839.1 924 EAAGKIATEAIENIRTVVSLTRERKFELMYGEHLIVPYRNSVKKAHIFGFCFALSQAMMFFTYAGCFRFGAYLVVNDLME 1003**

**XP_053808094.1 909 EAAGKIATEAIENIRTVVSLTRERKFESMYGEHLILPYRNSVKKAHIFGFCFALSQAMMFFTYAGCFRFGAYLVVNGHME 988**

**XP_068068048.1 908 EAAGKIATEAIENIRTVVSLTRERKFESMYGEHLILPYRNSVKKAHIFGFCFALSQAMMFFTYAGCFRFGAYLVVNGHME 987**

**XP_004186266.5 910 EAAGKIATEAIENIRTVVSLTLERKFELMYGEHLILPYRNSVKKAHIFGFCFALSQAMMFFTYAGCFRFGAYLVVNDHTE 989**

**XP_021407324.2 910 EAAGKIATEAIENIRTVVSLTLERKFELMYGEHLILPYRNSVKKAHIFGFCFALSQAMMFFTYAGCFRFGAYLVVNDHTE 989**

**XP_054129694.1 914 EAAGKIATEAIENIRTVVSLTRERKFELMYGEHLIVPYRNSVKKAHIFGFCFALSQAMMFFTYAGCFRFGAYLVVNDLME 993**

**XP_064560295.1 917 EAAGKIATEAIENIRTVVSLTRERKFELMYGEHLIVPYRNSVKKAHIFGFCFALSQAMMFFTYAGCFRFGAYLVVNDLME 996**

**XP_074391572.1 917 EAAGKIATEAIENIRTVVSLTRERKFELMYGEHLIVPYRNSVKKAHIFGFCFALSQAMMFFTYAGCFRFGAYLVVNDFME 996**

**XP_056339284.1 910 ESAGKIATQAIENIRTVASLTRERKFESMYGEYLIVPYRNSVKKAHIFGFCFALSQAMMFFTYAGCFRFGAYLVVNGHMQ 989**

**XP_036262392.1 914 EAAGKIATEAIENIRTVVSLTQERKFELMYGEHLIVPYRNSVKKAHIFGFCFALSQAMMFFTYAGCFRFGAYLVVNDLME 993**

**XP_066408995.1 914 EAAGKIATEAIENIRTVVSLTQERKFELMYGEHLIVPYRNSVKKAHIFGFCFALSQAMMFFTYAGCFRFGAYLVVNDLME 993**

**XP_054484009.2 914 EAAGKIATEAIENIRTVVSLTQERKFELMYGEHLIVPYRNSVKKAHIFGFCFALSQAMMFFTYAGCFRFGAYLVVNDLMK 993**

**XP_071285034.1 914 EAAGKIATEAIENIRTVVSLTQERKFELMYGEHLIVPYRNSVKKAHIFGFCFALSQAMMFFTYAGCFRFGAYLVVNDLME 993**

**XP_030922622.1 914 EAAGKIATEAIENIRTVVSLTRERKFELMYGEHLIVPYRNSVKKAHIFGFCFALSQAMMFFTYAGCFRFGAYLVVNDLME 993**

**XP_030826393.1 914 EAAGKIATEAIENIRTVVSLTRERKFELMYGEHLIVPYRNSVKKAHIFGFCFALSQAMMFFTYAGCFRFGAYLVVNDLME 993**

**XP_062350659.1 908 ETAGKIATQAIENIRTVVSLTRERKFESMYGEHLIVPYRNSVKKAHVFGFCFALSQAMMFFTYAGCFRFGAYLVVNGHME 987**

**XP_014731144.1 910 ETAGKVATEAIENIRTVVSLTRERKFELMYGEHLIVPYRNSVKKAHVFGFCFALSQAMMFFTYAGCFRFGAYLVVNGHMK 989**

**XP_032926696.1 915 ESAGKIATQAIENIRTVVSLTRERKFESMYGEYLIVPYRNSVKKAHIFGFCFALSQAMMFFTYAACFRFGAYLVVNGHME 994**

**XP_005518835.2 899 EAAGKIATEAIENIRTVVSLTRERKFESMYGEHLIVPYRNSVKKAHIFGFCFALSQAMMFFTYAGCFRFGAYLVVNGHME 978**

**XP_058687902.1 900 EAAGKIATEAIENIRTVVSLTRERKFESMYGEHLILPYRNSVKKAHIFGFCFALSQAMMFFTYAGCFRFGAYLVVNGHME 979**

**XP_015471143.1 900 EAAGKIATEAIENIRTVVSLTRERKFESMYGEHLIVPYRNSVKKAHIFGFCFALSQAMMFFTYAGCFRFGAYLVVNGHME 979**

**XP_023776043.1 900 EAAGKIATEAIENIRTVVSLTRERKFESMYGEHLVVPYRNSVKKAHIFGFCFALSQAMMFFTYAGCFRFGAYLVVNGYME 979**

**XP_058275891.1 896 ETAGKIATEAIENIRTVVSLTRERKFESMYGEHLIVPYRNSVKKAHIFGFCFALSQAMMFFTYAGCFRFGAYLVVHDHMN 975**

**XP_063268876.1 901 ETAGKIATEAIENIRTVVSLTRERKFESMYGEHLIVPYRNSVKKAHIFGFCFALSQAMMFFTYAGCFRFGAYLVVNDLME 980**

**XP_068862854.1 900 EAAGKIATEAIENIRTVVSLTRERKFESMYGEHLIVPYRNSVKKAHIFGFCFALSQAMMFFTYAGCFRFGAYLVVNKYME 979**

**XP_031968992.1 900 EAAGKIATEAIENIRTVVSLTRERKFESMYGEHLIVPYRNSVKKAHIFGFCFALSQAMMFFTYAGCFRFGAYLVVNGHME 979**

**XP_010397518.2 900 EAAGKIATEAIENIRTVVSLTRERKFESMYGEHLIVPYRNSVKKAHIFGFCFALSQAMMFFTYAGCFRFGAYLVVNDHME 979**

**XP_008628111.1 900 EAAGKIATEAIENIRTVVSLTRERKFESMYGEHLIVPYRNSVKKAHIFGFCFALSQAMMFFTYAGCFRFGAYLVVNDHME 979**

**XP_041899696.1 900 EAAGKIATEAIENIRTVVSLTRERKFESMYGEHLIVPYRNSVKKAHIFGFCFALSQAMMFFTYAGCFRFGAYLVVNEHME 979**

**XP_048162577.1 900 EAAGKIATEAIENIRTVVSLTRERKFESMYGEHLIVPYRNSVKKAHIFGFCFALSQAMMFFTYAGCFRFGAYLVVNGHMK 979**

**XP_066172927.1 912 ETAGKIATEAIENIRTVVSLTRERKFESMYGEHLIVPYRNSVKKAHIFGFCFALSQAMMFFTYAGCFRFGAYLVVNGHMV 991**

**XP_066054226.1 918 ETAGKIATEAIENIRTVVSLTREKKFESMYGEHLIVPYRNSVKKAHIFGFCFALSQAMMFFTYAGCFRFGAYLVVNGHMQ 997**

**XP_057239101.1 898 EAAGKIATEAIENIRTVVSLTRERKFESMYGEHLIVPYRNSVKKAHIFGLCFALSQAMMFFTYAACFRFGAYLVVNGHME 977**

**XP_039235593.1 900 EAAGKIATEAIENIRTVVSLTRERKFELMYGEHLIVPYRNSVKKAHIFGFCFALSQAMMFFTYAGCFRFGAYLVANGHME 979**

**XP_027564162.1 901 EAAGKIATEAIENIRTVVSLTRERKFELMYGEYLIVPYRNSVKKAHIFGFCFALSQAMMFFTYAGCFRFGAYLVANGHME 980**

**XP_051658500.1 900 EAAGKIATEAIENIRTVVSLTRERKFELMYGEHLIVPYRNSVKKAHIFGFCFALSQAMMFFTYAGCFRFGAYLVANGHME 979**

**XP_064534663.1 900 EAAGKIATEAIENIRTVVSLTRERKFELMYGEHLLVPYRNSVKKAHIFGFSFALSQAMMFFTYAACFRFGAYLVANGHME 979**

**CDM63410.1 999 YKSVFLVFSAVVFGAMALGQTSSFAPDYAKAKISAAHLFLLFERVPSIDSYSEEGDKPETFEGNITIKDVAFNYPNRPEV 1078**

**XP_059698659.1 1000 YKSVFLVFSAVVFGAMALGQTSSFAPDYAKAKISAAHLFLLFERVPSIDSYSEEGDKPETFEGNITIKDVAFNYPNRPEV 1079**

**XP_050827218.1 1000 YKSVFLVFSAVVFGAMALGQTSSFAPDYAKAKISAAHLFLLFERVPSIDSYSEEGDKPETFEGNITIKDVAFNYPNRPEV 1079**

**XP_037984436.1 999 YKTVFLVFSAVVFGAMALGQTSSFAPDYAKAKISAAHLFLLFERVPSIDSYSEEGDKPEAFEGNITIKDVAFNYPNRPEV 1078**

**XP_041322342.1 999 YKSVFLVFSAVVFGAMALGQTSSFAPDYAKAKISAAHLFLLFERVPSIDSYSEEGDKPETFEGNITIKDVAFNYPNRPEV 1078**

**XP_041264549.1 1000 YKSVFLVFSAVVFGAMALGQTSSFAPDYAKAKISAAHLFLLFERVPSIDSYSEEGDKPETFEGNITIKDVAFNYPNRPEV 1079**

**XP_039567137.1 1000 YKSVFLVFSAVVFGAMALGQTSSFAPDYAKAKISAAHLFLLFERVPSIDSYSEEGDKPETFEGNITIKDVAFNYPNRPEV 1079**

**XP_064265165.1 1000 YKSVFLVFSAVVFGAMALGQTSSFAPDYAKAKISAAHLFLLFERVPSIDSYSEEGDKPETFEGNITIKDVAFNYPNRPEV 1079**

**XP_016151870.1 1000 YKDVFLVFSAVVFGAMALGQTSSFAPDYAKAKISAAHLFLLFERVPSIDSYSEEGDKPETFDGNITIKDVVFNYPNRPEV 1079**

**XP_058670934.1 1004 FKSVFLVFSAVVFGAMALGQTSSFAPDYAKAKISAAHLFQLFERVPSIDSYSEEGDKPETFEGNITIKDVAFNYPNRPEV 1083**

**XP_059323184.1 1004 FKSVFLVFSAVVFGAMALGQTSSFAPDYAKAKISAAHLFQLFERVPSIDSYSEEGDKPETFEGNITIKDVAFNYPNRPEV 1083**

**XP_057876310.1 1004 FKSVFLVFSAVVFGAMALGQTSSFAPDYAKAKISAAHLFQLFERVPSIDSYSEEGDKPETFEGNITIKDVAFNYPNRPEV 1083**

**XP_063006839.1 1004 FKSVFLVFSAVVFGAMALGQTSSFAPDYAKAKISAAHLFQLFERVPSIDSYSEEGDKPETFEGNITIKDVAFNYPNRPEV 1083**

**XP_053808094.1 989 YKSVFLVFSAVVFGAMALGQTSSFAPDYAEAKISAAHLFLLFERVPSIDSYSEGGDKPETFEGNITIKDVAFNYPNRPEV 1068**

**XP_068068048.1 988 YKSVFLVFSAVVFGAMALGQTSSFAPDYAKAKISAAHLFLLFERVPSIDSYSEEGDKPETFEGNITMKDVAFNYPNRPEV 1067**

**XP_004186266.5 990 YKRVFLVFSAVVFGAMALGQTSSFAPDYAKAKISAAHLFLLFERVPSIDSYSEEGDKPETFEGNITMKDVAFNYPNRPEV 1069**

**XP_021407324.2 990 YKKVFLVFSAVVFGAMALGQSSSFAPDYAKAKISAAHLFLLFERVPSIDSYSEEGDKPETFEGNITMKDVAFNYPNRPEV 1069**

**XP_054129694.1 994 FKSVFLVFSAVVFGAMALGQTSSFAPDYAKAKISAAHLFQLFERVPSIDSYSEEGDKLETFEGNITIKDVAFNYPNRPEV 1073**

**XP_064560295.1 997 FKHVFLVFSAVVFGAMALGQTSSFAPDYAKAKISAAHLFQLFERVPSIDSYSEEGDKPETFEGNITIKDVAFNYPNRPEV 1076**

**XP_074391572.1 997 FKHVFLVFSAVVFGAMALGQTSSFAPDYAKAKISAAHLFQLFERVPSIDSYSEEGDKPETFEGNITIKDVAFNYPNRPEV 1076**

**XP_056339284.1 990 YKDVFLVFSAVVFGAMALGQTSSFAPDYAKAKISAAHLFLLFERVPSIDSYSEEGDKPETFDGNITIKDVVFNYPNRPEV 1069**

**XP_036262392.1 994 FKSVFLVFSAVVFGAMALGQTSSFAPDYAKAKISAAHLFQLFERVPSIDSYSEEGDKPETFEGNITVKDVVFNYPNRPEV 1073**

**XP_066408995.1 994 FKSVFLVFSAVVFGAMALGQTSSFAPDYAKAKISAAHLFQLFERVPSIDSYSEEGDKPETFEGNITVKDVVFNYPNRPEV 1073**

**XP_054484009.2 994 FKNVFLVFSAVVFGAMALGQTSSFAPDYAKAKISAAHLFQLFERVPSIDSYSEEGDKPETFQGNITIKDVVFNYPNRPEV 1073**

**XP_071285034.1 994 FKNVFLVFSAVVFGAMALGQTSSFAPDYAKAKISAAHLFQLFERVPSIDSYSEEGDKPETFQGNITIKDVVFNYPNRPEV 1073**

**XP_030922622.1 994 YKHVFLVFSAVVFGAMALGQTSSFAPDYAKAKISAAHLFQLFERVPSIDSYSEEGDKPETFEGNITIKDVAFNYPNRPEV 1073**

**XP_030826393.1 994 YKHVFLVFSAVVFGAMALGQTSSFAPDYAKAKISAAHLFQLFERVPSIDSYSEEGDKPETFEGNITIKDVAFNYPNRPEV 1073**

**XP_062350659.1 988 YKNVFLVFSAVVFGAMALGQTSSFAPDYAKAKISAAHLFLLFERVPSIDSYSEEGDKPETFEGNITIKDVVFNYPNRPEV 1067**

**XP_014731144.1 990 YQDVFLVFSAVVFGAMALGQTSSFAPDYAKAKISAAHLFLLFERVPSIDSYSEEGDKPETFEGNITVKDVVFNYPNRPEV 1069**

**XP_032926696.1 995 SRHVFLVFSAVVFGAMALGQTSSFAPDYAKGKISAAHLFLLFERVPSIDSYSEEGDKPETFDGNITIKDVIFNYPNRPQV 1074**

**XP_005518835.2 979 YKSVFLVFSAVVFGAMALGQTSSFAPDYAKAKISASHLFQLFERVPSIDSYSEEGDKLETFEGNITIKDVAFNYPNRPEV 1058**

**XP_058687902.1 980 YKSVFLVFSAVVFGAMALGQTSSFAPDYAKAKISASHLFQLFERVPSIDSYSEEGDKPETFEGNITIKDVAFNYPNRPEV 1059**

**XP_015471143.1 980 YKSVFLVFSAVVFGAMALGQTSSFAPDYAKAKISASHLFQLFERVPSIDSYSEEGDKPETFEGNITIRDVAFNYPNRPEV 1059**

**XP_023776043.1 980 YKSVFLVFSAVVFGAMALGQTSSFAPDYAKAKISASHLFQLFERVPSIDSYSEEGDKPETFEGNITIRDVAFNYPNRPEV 1059**

**XP_058275891.1 976 YKHVFLVFSAVVFGAMALGQTSSFAPDYAKAKISAAHLFMLFERVPSIDSYSEEGDKPETFEGNITIKDVAFNYPNRPEV 1055**

**XP_063268876.1 981 YKDVFLVFSAVVFGAMALGQTSSFAPDYAKAKISAAHLFMLFERVPSIDSYSEEGDKPETFEGNITIKDMAFNYPNRPEV 1060**

**XP_068862854.1 980 YKTVFLVFSAVVFGAMALGQTSSFAPDYAKAKISAAHLFLLFERVPSIDSYSEEGDKPETFEGNITIKDVAFNYPNRPEV 1059**

**XP_031968992.1 980 STSVFLVFSAVVFGAMALGQTSSFAPDYAKAKISAAHLFRLFERVPSIDSYSEEGDKPETFEGNITIKDVAFNYPNRPEV 1059**

**XP_010397518.2 980 STSVFLVFSAVVFGAMALGQTSSFAPDYAKAKISAAHLFRLFERVPSIDSYSEEGDKPETFEGNITIKDVAFNYPNRPEV 1059**

**XP_008628111.1 980 STSVFLVFSAVVFGAMALGQTSSFAPDYAKAKISAAHLFRLFERVPSIDSYSEEGDKPETFEGNITIKDVAFNYPNRPEV 1059**

**XP_041899696.1 980 STSVFLVFSAVVFGAMALGQTSSFAPDYAKAKISAAHLFRLFERVPSIDSYSEEGDKPETFEGNITIKDVAFNYPNRPEV 1059**

**XP_048162577.1 980 STSVFLVFSAVVFGAMALGQTSSFAPDYAKAKISAAHLFRLFERVPSIDSYSEEGDKPETFEGNITIKDVAFNYPNRPEV 1059**

**XP_066172927.1 992 YQDVFLVFSAVVFGAMALGQASSFAPDYAKAKISAAHLFLLFERVPSIDSYSEEGDKPETFEGNITIKDVAFNYPNRPEV 1071**

**XP_066054226.1 998 YKDVFLVFSAVVFGAMALGQTSSFAPDYAKAKISAAHLFLLFERVPSIDSYSEEGDKPETFEGNITIKDVAFNYPNRPEV 1077**

**XP_057239101.1 978 YKSVFLVFSAVVFGAMALGQTSSFAPDYAKAKISAAHLFLLFERVPAIDSYSEEGDKPETFEGNITIKDVAFNYPNRPEV 1057**

**XP_039235593.1 980 YKSVFLVFSAVVFGAMALGQTSSFAPDYAKAKISAAHLFQLFERVPSIDSYSEEGEKPETFEGNITIKDVAFNYPNRPEV 1059**

**XP_027564162.1 981 YKSVFLVFSAVVFGAMALGQTSSFAPDYAKAKISAAHLFQLFERVPSIDSYSEEGEKPETFEGNITIKDVAFNYPNRPEV 1060**

**XP_051658500.1 980 YKSVFLVFSAVVFGAMALGQTSSFAPDYAKAKISAAHLFQLFERVPSIDSYSEEGQKPETFEGNITIKDVAFNYPNRPEV 1059**

**XP_064534663.1 980 YKSVFLVFSAVVFGAMALGQTSSFAPDYAKAKISAAHLFQLFERVPSIDSYSEEGEKPETFEGNITIKDVVFNYPNRPEV 1059**

**CDM63410.1 1079 KILQGLNLKVEKGQTLALVGSSGCGKSTVVQLLERFYDPLDGELLFDGKTAKALNIQWLRAQIGIVSQEPILFDFTIAEN 1158**

**XP_059698659.1 1080 KILQGLNLKVEKGQTLALVGSSGCGKSTVVQLLERFYDPLDGELLFDGKTAKALNIQWLRAQIGIVSQEPILFDFTIAEN 1159**

**XP_050827218.1 1080 KILQGLNLKVEKGQTLALVGSSGCGKSTVVQLLERFYDPLDGELFFDGKTAKALNIQWLRAQIGIVSQEPILFDFTIAEN 1159**

**XP_037984436.1 1079 KILQGLNLKVEKGQTLALVGSSGCGKSTVVQLLERFYDPLDGEMLFDGKNAKALNIQWLRAQIGIVSQEPILFDFTIAEN 1158**

**XP_041322342.1 1079 KILQGLNLKVEKGQTLALVGSSGCGKSTVVQLLERFYDPLDGEMLFDGKTAKALNIQWLRAQIGIVSQEPILFDFTIAEN 1158**

**XP_041264549.1 1080 KILQGLNLKVEKGQTLALVGSSGCGKSTVVQLLERFYDPLDGEMLFDGKTAKALNIQWLRAQIGIVSQEPILFDFTIAEN 1159**

**XP_039567137.1 1080 KILQGLNLKVEKGQTLALVGSSGCGKSTVVQLLERFYDPLDGEMLFDGKTAKSLNIQWLRSQIGIVSQEPILFDFTIAEN 1159**

**XP_064265165.1 1080 KILQGLNLKVEKGQTLALVGSSGCGKSTVVQLLERFYDPLDGEMLFDGKTAKSLNIQWLRAQIGIVSQEPILFDFTIAEN 1159**

**XP_016151870.1 1080 KILQGLNLKVEKGQTLALVGSSGCGKSTVVQLLERFYDPLEGEMLFDGKNAKALNIQWLRAQIGIVSQEPILFDFTIAEN 1159**

**XP_058670934.1 1084 KILQGLNLKVEKGQTLALVGSSGCGKSTVVQLLERFYDPLDGEMFFDGKTAKALNIQWLRAQIGIVSQEPILFDFTIAEN 1163**

**XP_059323184.1 1084 KILQGLNLKVEKGQTLALVGSSGCGKSTVVQLLERFYDPLDGEMFFDGKTAKALNIQWLRAQIGIVSQEPILFDFTIAEN 1163**

**XP_057876310.1 1084 KILQGLNLKVEKGQTLALVGSSGCGKSTVVQLLERFYDPLDGEMFFDGKTAKALNIQWLRAQIGIVSQEPILFDFTIAEN 1163**

**XP_063006839.1 1084 KILQGLNLKVEKGQTLALVGSSGCGKSTVVQLLERFYDPLDGEMFFDGKTAKALNIQWLRAQIGIVSQEPILFDFTIAEN 1163**

**XP_053808094.1 1069 KILQGLNLKVEKGQTLALVGSSGCGKSTVVQLLERFYDPLDGEMLFDGKNAKALNIQWLRAQIGIVSQEPILFDFTIAEN 1148**

**XP_068068048.1 1068 KILQGLNLKVEKGQTLALVGSSGCGKSTVVQLLERFYDPLDGEMLFDGKNAKALNIQWLRAQIGIVSQEPILFDFTIAEN 1147**

**XP_004186266.5 1070 KILQGLNLKVEKGQTLALVGSSGCGKSTVVQLLERFYDPLDGEMIFDGKNAKALNIQWLRAQIGIVSQEPILFDCTIAEN 1149**

**XP_021407324.2 1070 KILQGLNLKVEKGQTLALVGSSGCGKSTVVQLLERFYDPLDGEMIFDGKNAKALNIQWLRAQIGIVSQEPILFDCTIAEN 1149**

**XP_054129694.1 1074 KILQGLNLKVEKGQTLALVGSSGCGKSTVVQLLERFYDPLDGEMFFDGKTAKALNIQWLRAQIGIVSQEPILFDFTIAEN 1153**

**XP_064560295.1 1077 KILQGLNLKVEKGQTLALVGSSGCGKSTVVQLLERFYDPLDGEMFFDGKTAKALNIQWLRAQIGIVSQEPILFDFTIAEN 1156**

**XP_074391572.1 1077 KILQGLNLKVEKGQTLALVGSSGCGKSTVVQLLERFYDPLDGEMFFDGKTAKALNIQWLRAQIGIVSQEPILFDFTIAEN 1156**

**XP_056339284.1 1070 KILQGLNLKVEKGQTLALVGSSGCGKSTVVQLLERFYDPLEGEMLFDGKNAKALNIQWLRAQIGIVSQEPILFDFTIAEN 1149**

**XP_036262392.1 1074 KILQGLNLKVEKGQTLALVGSSGCGKSTVVQLLERFYDPLDGEMFFDGKTAKALNIQWLRAQIGIVSQEPILFDFTIAEN 1153**

**XP_066408995.1 1074 KILQGLNLKVEKGQTLALVGSSGCGKSTVVQLLERFYDPLDGEMFFDGKTAKALNIQWLRAQIGIVSQEPILFDFTIAEN 1153**

**XP_054484009.2 1074 KILQGLNLKVEKGQTLALVGSSGCGKSTVVQLLERFYDPLDGEMFFDGKTAKALNIQWLRAQIGIVSQEPILFDFTIAEN 1153**

**XP_071285034.1 1074 KILQGLNLKVEKGQTLALVGSSGCGKSTVVQLLERFYDPLDGEMFFDGKTAKALNIQWLRAQIGIVSQEPILFDFTIAEN 1153**

**XP_030922622.1 1074 KILQGLNLKVEKGQTLALVGSSGCGKSTVVQLLERFYDPLDGEMLFDGKSAKSLNIQWLRAQIGIVSQEPILFDCTIAEN 1153**

**XP_030826393.1 1074 KILQGLNLKVEKGQTLALVGSSGCGKSTVVQLLERFYDPLDGEMLFDGKSAKSLNIQWLRAQIGIVSQEPILFDCTIAEN 1153**

**XP_062350659.1 1068 KILQGLNLKVEKGQTLALVGSSGCGKSTVVQLLERFYDPLDGEMLFDGKNAKALNIQWLRAHIGIVSQEPILFDFTIAEN 1147**

**XP_014731144.1 1070 KILQGLNLKVEKGQTLALVGSSGCGKSTIVQLLERFYDPLDGEMLFDGKNAKALNIQWLRAQIGIVSQEPILFDFTIAEN 1149**

**XP_032926696.1 1075 KILQGLNLKVEKGQTLALVGSSGCGKSTVVQLLERFYDPLEGEMLFDGKNAKTLNIQWLRSQIGIVSQEPILFDFTIAEN 1154**

**XP_005518835.2 1059 RILQGLNLKVEKGQTLALVGSSGCGKSTVVQLLERFYDPLDGEMLFDGKNAKALNVQWLRAQIGIVSQEPILFDFTIAEN 1138**

**XP_058687902.1 1060 KILQGLNLKVEKGQTLALVGSSGCGKSTVVQLLERFYDPLDGEMLFDGKNAKALNIQWLRAQIGIVSQEPILFDFTIAEN 1139**

**XP_015471143.1 1060 KILQGLNLKVEKGQTLALVGSSGCGKSTVVQLLERFYDPLDGEMLFDGKNAKALNIQWLRAQIGIVSQEPILFDFTIAEN 1139**

**XP_023776043.1 1060 KILQGLNVKVEKGQTLALVGSSGCGKSTVVQLLERFYDPLDGEMLFDGKNAKALNIQWLRAQIGIVSQEPILFDFTIAEN 1139**

**XP_058275891.1 1056 KILQGLNLKVEKGQTLALVGSSGCGKSTVVQLLERFYDPLDGEMLFDGKNAKALNIQWLRAQIGIVSQEPILFDFTIAEN 1135**

**XP_063268876.1 1061 KILQGLNLKVEKGQTLALVGSSGCGKSTVVQLLERFYDPLDGEMLFDGKNAKELNIQWLRAHIGIVSQEPILFDFTIAEN 1140**

**XP_068862854.1 1060 KILQGLNLKVEKGQTLALVGSSGCGKSTVVQLLERFYDPLDGEMLFDGKNAKALNIQWLRAQIGIVSQEPILFDFTIAEN 1139**

**XP_031968992.1 1060 KILQGLNLKVEKGQTLALVGSSGCGKSTVVQLLERFYDPLDGEMLFDGKNAKALNIQWLRAQIGIVSQEPILFDFTIAEN 1139**

**XP_010397518.2 1060 KILQGLNLKVEKGQTLALVGSSGCGKSTVVQLLERFYDPLDGEMLFDGKNAKALNIQWLRAQIGIVSQEPILFDFTIAEN 1139**

**XP_008628111.1 1060 KILQGLNLKVEKGQTLALVGSSGCGKSTVVQLLERFYDPLDGEMLFDGKNAKALNIQWLRAQIGIVSQEPILFDFTIAEN 1139**

**XP_041899696.1 1060 KILQGLNLKVEKGQTLALVGSSGCGKSTVVQLLERFYDPLDGEMLFDGKNAKALNIQWLRAQIGIVSQEPILFDFTIAEN 1139**

**XP_048162577.1 1060 KILQGLNLKVEKGQTLALVGSSGCGKSTVVQLLERFYDPLDGEMLFDGKNAKALNIQWLRAQIGIVSQEPILFDFTIAEN 1139**

**XP_066172927.1 1072 KILQGLNLKVEKGQTLALVGSSGCGKSTVVQLLERFYDPLDGEMLFDGKNAKALNIQWLRAQIGIVSQEPILFDFTIAEN 1151**

**XP_066054226.1 1078 KILQGLNLNVEKGQTLALVGSSGCGKSTVVQLLERFYDPLDGEMLFDGKNAKALNIQWLRAQIGIVSQEPILFDFTIAEN 1157**

**XP_057239101.1 1058 KILQGLNLKVEKGQTLALVGSSGCGKSTVVQLLERFYDPLDGEMLFDGKDAKALNIQWLRAQIGIVSQEPILFDFTIAEN 1137**

**XP_039235593.1 1060 KILQGLNLQVEKGQTLALVGSSGCGKSTVVQLLERFYDPLDGEMLFDGKGAKALNIKWLRAQIGIVSQEPILFDCTIAEN 1139**

**XP_027564162.1 1061 KILQGLNLKAEKGQTLALVGSSGCGKSTVVQLLERFYDPLDGEMLFDGKGAKALNIKWLRAQIGIVSQEPILFDCTIAEN 1140**

**XP_051658500.1 1060 KILQGLNLKVEKGQTLALVGSSGCGKSTVVQLLERFYDPLDGEMLFDGKGAKALNIKWLRAQIGIVSQEPILFDCTIAEN 1139**

**XP_064534663.1 1060 KILQGLNLKVEKGQTLALVGSSGCGKSTVVQLLERFYDPLDGEMLFDGKGAKALNIKWLRAQIGIVSQEPILFDCTIAEN 1139**

**CDM63410.1 1159 IAYGDNSRQVSFEEIVSAAKQANIHSFIDSLPDKYNTRVGDKGTQLSGGQKQRIAIARALVRKPQILLLDEATSALDTES 1238**

**XP_059698659.1 1160 IAYGDNSRQVSFEEIVSAAKQANIHSFIDSLPDKYNTRVGDKGTQLSGGQKQRIAIARALVRKPQILLLDEATSALDTES 1239**

**XP_050827218.1 1160 IAYGDNSRQVSFEEIVSAAKQANIHSFIDSLPDKYNTRVGDKGTQLSGGQKQRIAIARALVRKPQILLLDEATSALDTES 1239**

**XP_037984436.1 1159 IAYGDNSRQVPFEEIVSAAKQANIHSFIDSLPDKYNTRVGDKGTQLSGGQKQRIAIARALVRKPQILLLDEATSALDTES 1238**

**XP_041322342.1 1159 IAYGDNSRQVSHEEIVSAAKQANIHSFIDSLPDKYNTRVGDKGTQLSGGQKQRIAIARALVRKPQILLLDEATSALDTES 1238**

**XP_041264549.1 1160 IAYGDNSRQVSHEEIVSAAKQANIHSFIDSLPDKYNTRVGDKGTQLSGGQKQRIAIARALVRKPQILLLDEATSALDTES 1239**

**XP_039567137.1 1160 IAYGDNSRQVSHEEIVSAAKQANIHSFIDSLPDKYNTRVGDKGTQLSGGQKQRIAIARALVRKPQILLLDEATSALDTES 1239**

**XP_064265165.1 1160 IAYGDNSRQVSHEEIVSAAKQANIHSFIDSLPDKYNTRVGDKGTQLSGGQKQRIAIARALVRKPQILLLDEATSALDTES 1239**

**XP_016151870.1 1160 IAYGDNSRQVPFEEIVSAAKEANIHSFIDSLPNKYNTRVGDKGTQLSGGQKQRITIARALVRKPQILLLDEATSALDTES 1239**

**XP_058670934.1 1164 IAYGDNSRQVSFEEIVSAAKQANIHSFIDSLPDKYNTRVGDKGTQLSGGQKQRIAIARALVRRPQILLLDEATSALDTES 1243**

**XP_059323184.1 1164 IAYGDNSRQVSFEEIVSAAKQANIHSFIDSLPDKYNTRVGDKGTQLSGGQKQRIAIARALVRRPQILLLDEATSALDTES 1243**

**XP_057876310.1 1164 IAYGDNSRQVSFEEIVSAAKQANIHSFIESLPDKYNTRVGDKGTQLSGGQKQRIAIARALVRRPQILLLDEATSALDTES 1243**

**XP_063006839.1 1164 IAYGDNSRQVSFEEIVSAAKQANIHSFIDSLPDKYNTRVGDKGTQLSGGQKQRIAIARALVRRPQILLLDEATSALDTES 1243**

**XP_053808094.1 1149 IAYGDNSREVSFEEIVSAAKQANIHSFIDSLPDKYNTRVGDKGTQLSGGQKQRIAIARALVRKPQILLLDEATSALDTES 1228**

**XP_068068048.1 1148 IAYGDNSREVSFEEIVSAAKQANIHSFIDSLPDKYNTRVGDKGTQLSGGQKQRVAIARALVRKPQILLLDEATSALDTES 1227**

**XP_004186266.5 1150 IAYGDNSREVSFEEIVSAAKQANIHSFIDSLPDKYNTRVGDKGTQLSGGQKQRIAIARALVRKPQILLLDEATSALDTES 1229**

**XP_021407324.2 1150 IAYGDNSREVSFEEIVSAAKQANIHSFIDSLPDKYNTRVGDKGTQLSGGQKQRIAIARALVRKPQILLLDEATSALDTES 1229**

**XP_054129694.1 1154 IAYGDNSRQVSFEEIVSAAKQANIHSFIDSLPDKYNTRVGDKGTQLSGGQKQRIAIARALVRRPQILLLDEATSALDTES 1233**

**XP_064560295.1 1157 IAYGDNSRQVSFEEIVSAAKQANIHSFIDSLPDKYNTRVGDKGTQLSGGQKQRIAIARALVRRPQILLLDEATSALDTES 1236**

**XP_074391572.1 1157 IAYGDNSRQVSFEEIVSAAKQANIHSFIDSLPDKYNTRVGDKGTQLSGGQKQRIAIARALVRRPQILLLDEATSALDTES 1236**

**XP_056339284.1 1150 IAYGDNSRQVPFEEIVSAAKEANIHSFIDSLPNKYNTRVGDKGTQLSGGQKQRIAIARALVRKPQILLLDEATSALDTES 1229**

**XP_036262392.1 1154 IAYGDNSRQVSFEEIVSAAKQANIHSFIDSLPDKYNTRVGDKGTQLSGGQKQRIAIARALVRRPQILLLDEATSALDTES 1233**

**XP_066408995.1 1154 IAYGDNSRQVSFEEIVSAAKQANIHSFIDSLPDKYNTRVGDKGTQLSGGQKQRIAIARALIRRPQILLLDEATSALDTES 1233**

**XP_054484009.2 1154 IAYGDNSRQVSFEEIVSAAKQANIHSFIDSLPDKYNTRVGDKGTQLSGGQKQRIAIARALVRRPQILLLDEATSALDTES 1233**

**XP_071285034.1 1154 IAYGDNSRQVSFEEIVSAAKQANIHSFIDSLPDKYNTRVGDKGTQLSGGQKQRIAIARALVRRPQILLLDEATSALDTES 1233**

**XP_030922622.1 1154 IAYGDNSRQVSFEEIVSAAKQANIHSFIDSLPDKYNTRVGDKGTQLSGGQKQRIAIARALVRRPQILLLDEATSALDTES 1233**

**XP_030826393.1 1154 IAYGDNSRQVSFEEIVSAAKQANIHSFIDSLPDKYNTRVGDKGTQLSGGQKQRIAIARALVRRPQILLLDEATSALDTES 1233**

**XP_062350659.1 1148 IAYGDNSRQVSFEEIVSAAKEANIHSFIDSLPDKYNTRVGDKGTQLSGGQKQRIAIARALVRKPQILLLDEATSALDTES 1227**

**XP_014731144.1 1150 IAYGDNSRQVSFEEIVSAAKEANIHSFIDSLPDKYNTRVGDKGTQLSGGQKQRIAIARALVRKPQILLLDEATSALDTES 1229**

**XP_032926696.1 1155 IAYGDNSREVPFEEIVNAAKEANIHSFIDSLPNKYNTRVGDKGTQLSGGQKQRIAIARALVRKPQILLLDEATSALDTES 1234**

**XP_005518835.2 1139 IAYGDNSRQVSFEEIVRAAKEANIHSFIDSLPDKYNTRVGDKGTQLSGGQKQRIAIARALVRKPQILLLDEATSALDTES 1218**

**XP_058687902.1 1140 IAYGDNSRQVSFEEIVRAAKEANIHSFIDSLPDKYNTRVGDKGTQLSGGQKQRIAIARALVRKPQILLLDEATSALDTES 1219**

**XP_015471143.1 1140 IAYGDNSRQVSFEEIVRAAKEANIHSFIDSLPDKYNTRVGDKGTQLSGGQKQRIAIARALVRKPQILLLDEATSALDTES 1219**

**XP_023776043.1 1140 IAYGDNSRQVSFEEIVRAAKEANIHSFIDSLPDKYNTRVGDKGTQLSGGQKQRIAIARALVRKPQILLLDEATSALDTES 1219**

**XP_058275891.1 1136 IAYGDNSRQVSFEEIVSAAKEANIHSFIDSLPDKYNTRVGDKGTQLSGGQKQRIAIARALVRKPQILLLDEATSALDTES 1215**

**XP_063268876.1 1141 IAYGDNSRQVSFEEIVSAAKEANIHSFIDSLPDKYNTRVGDKGTQLSGGQKQRIAIARALVRKPQILLLDEATSALDTES 1220**

**XP_068862854.1 1140 IAYGDNSRQVSFEEIVSAAKEANIHSFIDSLPNKYNTRVGDKGTQLSGGQKQRIAIARALVRKPQILLLDEATSALDTES 1219**

**XP_031968992.1 1140 IAYGDNSRQVSFEEIVSAAKEANIHSFIDSLPNKYNTRVGDKGTQLSGGQKQRIAIARALVRKPHILLLDEATSALDTES 1219**

**XP_010397518.2 1140 IAYGDNSRQVSFEEIVSAAKEANIHSFIDSLPNKYNTRVGDKGTQLSGGQKQRIAIARALVRKPHILLLDEATSALDTES 1219**

**XP_008628111.1 1140 IAYGDNSRQVSFEEIVSAAKEANIHSFIDSLPNKYNTRVGDKGTQLSGGQKQRIAIARALVRKPHILLLDEATSALDTES 1219**

**XP_041899696.1 1140 IAYGDNSRQVSFEEIVSAAKEANIHSFIDSLPNKYNTRVGDKGTQLSGGQKQRIAIARALVRKPHILLLDEATSALDTES 1219**

**XP_048162577.1 1140 IAYGDNSRQVSFEEIVSAAKEANIHSFIDSLPNKYNTRVGDKGTQLSGGQKQRIAIARALVRKPHILLLDEATSALDTES 1219**

**XP_066172927.1 1152 IAYGDNSRQVSFEEIVTAAKEANIHSFIESLPDKYNTRVGDKGTQLSGGQKQRIAIARALVRKPQILLLDEATSALDTES 1231**

**XP_066054226.1 1158 IAYGDNSRQVSFEEIVTAAKEANIHSFIDSLPDKYNTRVGDKGTQLSGGQKQRIAIARALVRKPQILLLDEATSALDTES 1237**

**XP_057239101.1 1138 IAYGDNSRQVSFEEIVSAAKEANIHSFIESLPDKYNTRVGDKGTQLSGGQKQRIAIARALVRRPQILLLDEATSALDTES 1217**

**XP_039235593.1 1140 IAYGDNSRQVPFEEIVNAAKEANIHTFVDSLPDKYNTRVGDKGTQLSGGQKQRIAIARALVRQPQILLLDEATSALDTES 1219**

**XP_027564162.1 1141 IAYGDNSRQVPFEEIVNVAKKANIHTFIDSLPNKYNTRVGDKGTQLSGGQKQRIAIARALVRQPQILLLDEATSALDTES 1220**

**XP_051658500.1 1140 IAYGDNSRQVPFEEIVNAAKEANIHTFVDSLPDKYNTRVGDKGTQLSGGQKQRIAIARALVRRPQILLLDEATSALDTES 1219**

**XP_064534663.1 1140 IAYGDNSRQVPFEEIVNAAKEANIHTFVDSLPDKYNTRVGDKGTQLSGGQKQRIAIARALVRRPQILLLDEATSALDTES 1219**

**CDM63410.1 1239 EKIVQEALDKAREGRTCIVIAHRLSTIQNADRIAVVQNGRVTEQGTHQQLLAEKGVYYSLVNVQSG- 1304**

**XP_059698659.1 1240 EKIVQEALDKAREGRTCIVIAHRLSTIQNADKIAVVQNGRVTEQGTHQQLLAEKGVYYSLVNVQSG- 1305**

**XP_050827218.1 1240 EKIVQEALDKAREGRTCIVIAHRLSTIQNADKIAVVQNGRVTEQGTHQQLLAEKGVYYSLVNVQS-- 1304**

**XP_037984436.1 1239 EKIVQEALDKAREGRTCIVIAHRLSTIQNADKIAVVQNGRVTEQGTHQQLLAEKGVYYSLVNVQSG- 1304**

**XP_041322342.1 1239 EKIVQEALDKAQEGRTCIVIAHRLSTIQNADKIAVVQNGRVIEQGTHQQLLAEKGVYYSLVNVQSG- 1304**

**XP_041264549.1 1240 EKIVQEALDKAREGRTCIVIAHRLSTIQNADKIAVVQNGRVIEQGTHQQLLAEKGVYYSLVNVQSG- 1305**

**XP_039567137.1 1240 EKIVQEALDKAREGRTCIVIAHRLSTIQNADKIAVVQNGRVTEQGTHQQLLAEKGIYYSLVNVQSG- 1305**

**XP_064265165.1 1240 EKIVQEALDKAREGRTCIVIAHRLSTIQNADKIAVVQNGRVTEQGTHQQLLAEKGVYYSLVNVQSG- 1305**

**XP_016151870.1 1240 EKIVQEALDKAREGRTCIVIAHRLSTIQNADKIAVVQNGTVVEQGTHQQLLAEKGIYYSLVNVQNG- 1305**

**XP_058670934.1 1244 EKIVQEALDKAREGRTCIVIAHRLSTIQNADTIAVIQNGRVVEQGTHQQLLAEKGAYYSLVNVQSG- 1309**

**XP_059323184.1 1244 EKIVQEALDKAREGRTCIVIAHRLSTIQNADTIAVIQNGRVVEQGTHQQLLAEKGAYYSLVNVQSG- 1309**

**XP_057876310.1 1244 EKIVQEALDKAREGRTCIVIAHRLSTIQNADTIAVIQNGRVIEQGTHQQLLAEKGAYYSLVNVQSG- 1309**

**XP_063006839.1 1244 EKIVQEALDKAREGRTCIVIAHRLSTIQNADTIAVIQNGRVVEQGTHQQLLAEKGAYYSLVNVQSG- 1309**

**XP_053808094.1 1229 EKVVQEALDKAREGRTCIVIAHRLSTIQNADKISVVQNGRVVEQGTHQQLLAEKGIYYSLVNVQSG- 1294**

**XP_068068048.1 1228 EKIVQEALDKAREGRTCIVIAHRLSTIQNADKISVVQNGRVVEQGTHQQLLAEKGIYYSLVNVQSG- 1293**

**XP_004186266.5 1230 EKIVQEALDKAREGRTCIVIAHRLSTIQNADKISVVQNGRVVEQGTHQQLLAEKGIYYSLVNVQSG- 1295**

**XP_021407324.2 1230 EKIVQEALDKAREGRTCIVIAHRLSTIQNADKISVVQNGRVVEQGTHQQLLAEKGIYYSLVNVQSG- 1295**

**XP_054129694.1 1234 EKIVQEALDKAREGRTCIVIAHRLSTIQNADTIAVIQNGRVVEQGTHQQLLAEKGAYYSLVNVQSG- 1299**

**XP_064560295.1 1237 EKIVQEALDKAREGRTCIVIAHRLSTIQNADKIAVIQNGRVIEQGTHQQLLAEKGAYYSLVNVQSG- 1302**

**XP_074391572.1 1237 EKIVQEALDKAREGRTCIVIAHRLSTIQNADKIAVIQNGRVIEQGTHQQLLAEKGAYYSLVNVQSG- 1302**

**XP_056339284.1 1230 EKIVQEALDKAREGRTCIVIAHRLSTIQNADKIAVVQNGTVVEQGTHQQLLAEKGIYYSLVNVQNG- 1295**

**XP_036262392.1 1234 EKIVQEALDKAREGRTCIVIAHRLSTIQNADRIAVLQNGRVTEQGTHQQLLAEKGVYYSLVNVQSG- 1299**

**XP_066408995.1 1234 EKIVQEALDKAREGRTCIVIAHRLSTIQNADKIAVLQNGRVTEQGTHQQLLAEKGVYYSLVNVQSG- 1299**

**XP_054484009.2 1234 EKIVQEALDKAREGRTCIVIAHRLSTIQNADKIAVLQNGRVTEQGTHQQLLAEKGVYYSLVNVQSG- 1299**

**XP_071285034.1 1234 EKIVQEALDKAREGRTCIVIAHRLSTIQNADKIAVLQNGRVTEQGTHQQLLAEKGVYYSLVNVQSG- 1299**

**XP_030922622.1 1234 EKIVQEALDKAREGRTCIVIAHRLSTIQNADKIVVLQNGRVTEQGTHQQLLAEKGVYYSLVNVQSG- 1299**

**XP_030826393.1 1234 EKIVQEALDKAREGRTCIVIAHRLSTIQNADKIVVLQNGRVTEQGTHQQLLAEKGVYYSLVNVQSG- 1299**

**XP_062350659.1 1228 EKIVQEALDKAREGRTCIVIAHRLSTIQNADKIAVVQNGRVVEQGTHQQLLAEKGIYYSLVNVQSG- 1293**

**XP_014731144.1 1230 EKIVQEALDKAREGRTCIVIAHRLSTIQNADKIAVVQNGKVVEQGTHQQLLAEKGIYYSLVNVQSG- 1295**

**XP_032926696.1 1235 EKIVQEALDKAREGRTCIVIAHRLSTIQNADKIAVVQNGRIVEQGTHQQLLAEKGIYYSLVNVQSG- 1300**

**XP_005518835.2 1219 EKIVQEALDKAREGRTCIVIAHRLSTIQNADKIAVVQSGKIIEQGTHQQLLAEKGVYYSLVNVQSG- 1284**

**XP_058687902.1 1220 EKIVQEALDKAREGRTCIVIAHRLSTIQNADKIAVVQNGKIIEQGTHQQLLAEKGVYYSLVNVHSG- 1285**

**XP_015471143.1 1220 EKIVQEALDKAREGRTCIVIAHRLSTIQNADKIAVVQNGKVIEQGTHQQLLAEKGVYYSLVNVQSG- 1285**

**XP_023776043.1 1220 EKIVQEALDKAREGRTCIVIAHRLSTIQNADKIAVVQNGKIIEQGTHQQLLAERGVYYSLVNVQSG- 1285**

**XP_058275891.1 1216 EKIVQEALDKAREGRTCIVIAHRLSTIQNADKIAVVQNGRVIEQGTHQQLLAEKGIYYSLVNVQSG- 1281**

**XP_063268876.1 1221 EKIVQEALDKAREGRTCIVIAHRLSTIQNADKIAVVQNGRVIEQGTHQQLLAEKGVYYSLVNVQSG- 1286**

**XP_068862854.1 1220 EKIVQEALDKAREGRTCIVIAHRLSTIQNADKIAVVQNGKVVEQGTHQQLLAEKGIYYSLVNVQSG- 1285**

**XP_031968992.1 1220 EKIVQEALDKAREGRTCIVIAHRLSTIQNADKIAVVQNGKVVEQGTHQQLLAEKGIYYSLVNVQSG- 1285**

**XP_010397518.2 1220 EKIVQEALDKAREGRTCIVIAHRLSTIQNADKIAVVQNGKVVEQGTHQQLLAEKGIYYSLVNVQSG- 1285**

**XP_008628111.1 1220 EKIVQEALDKAREGRTCIVIAHRLSTIQNADKIAVVQNGKVVEQGTHQQLLAEKGIYYSLVNVQSG- 1285**

**XP_041899696.1 1220 EKIVQEALDKAREGRTCIVIAHRLSTIQNADKIAVVQNGKVVEQGTHQQLLAEKGIYYSLVNVQSG- 1285**

**XP_048162577.1 1220 EKIVQEALDKAREGRTCIVIAHRLSTIQNADKIAVVQNGKVVEQGTHQQLLAEKGIYYSLVNVQSG- 1285**

**XP_066172927.1 1232 EKIVQEALDKAREGRTCIVIAHRLSTXQNADKIAVVQNGKVIEQGTHQQLLAEKGVYYSLVNVQNV- 1297**

**XP_066054226.1 1238 EKIVQEALDKAREGRTCIVIAHRLSTIQNADKIAVVQNGRVIEQGTHQQLLAEKGVYYSLVNVQSV- 1303**

**XP_057239101.1 1218 EKIVQEALDKAREGRTCIVIAHRLSTIQNADKIAVVQNGVVVEQGTHQQLLAEKGIYYSLVNVQNIA 1284**

**XP_039235593.1 1220 EKIVQEALDKAREGRTCIVIAHRLSTVQNADKIAVIQNGKVVEQGTHQQLLAEKGIYYSLVNVQSV- 1285**

**XP_027564162.1 1221 EKIVQEALDKAREGRTCIVIAHRLSTVQNADKIAVIQNGKVVEQGTHQQLLAEKGIYYSLVNVQSV- 1286**

**XP_051658500.1 1220 EKIVQEALDKAREGRTCIVIAHRLSTVQNADKIAVIQNGKVVEQGTHQQLLAEKGIYYSLVNVQSV- 1285**

**XP_064534663.1 1220 EKIVQEALDKAREGRTCIVIAHRLSTVQNADKIAVIQNGKVVEQGTHQQLLAEKGIYYSLVNVQSV- 1285**

**Supplementary Figure 1: Multi sequence alignment of ABCB1 from *Passeriformes* species.** The alignment was performed with the NCBI constraint-based multiple alignment tool COBALT (www.ncbi.nlm.nih.gov/tools/cobalt/) with standard settings, including all ABCB1 protein sequences from *Passeriformes* species listed in **Table 2**. The 10-aa motif insertion with the consensus sequence NVLPSSENYE is present as single insertion, tandem repeat insertion, or triple repeat insertion in different passerine birds.

**
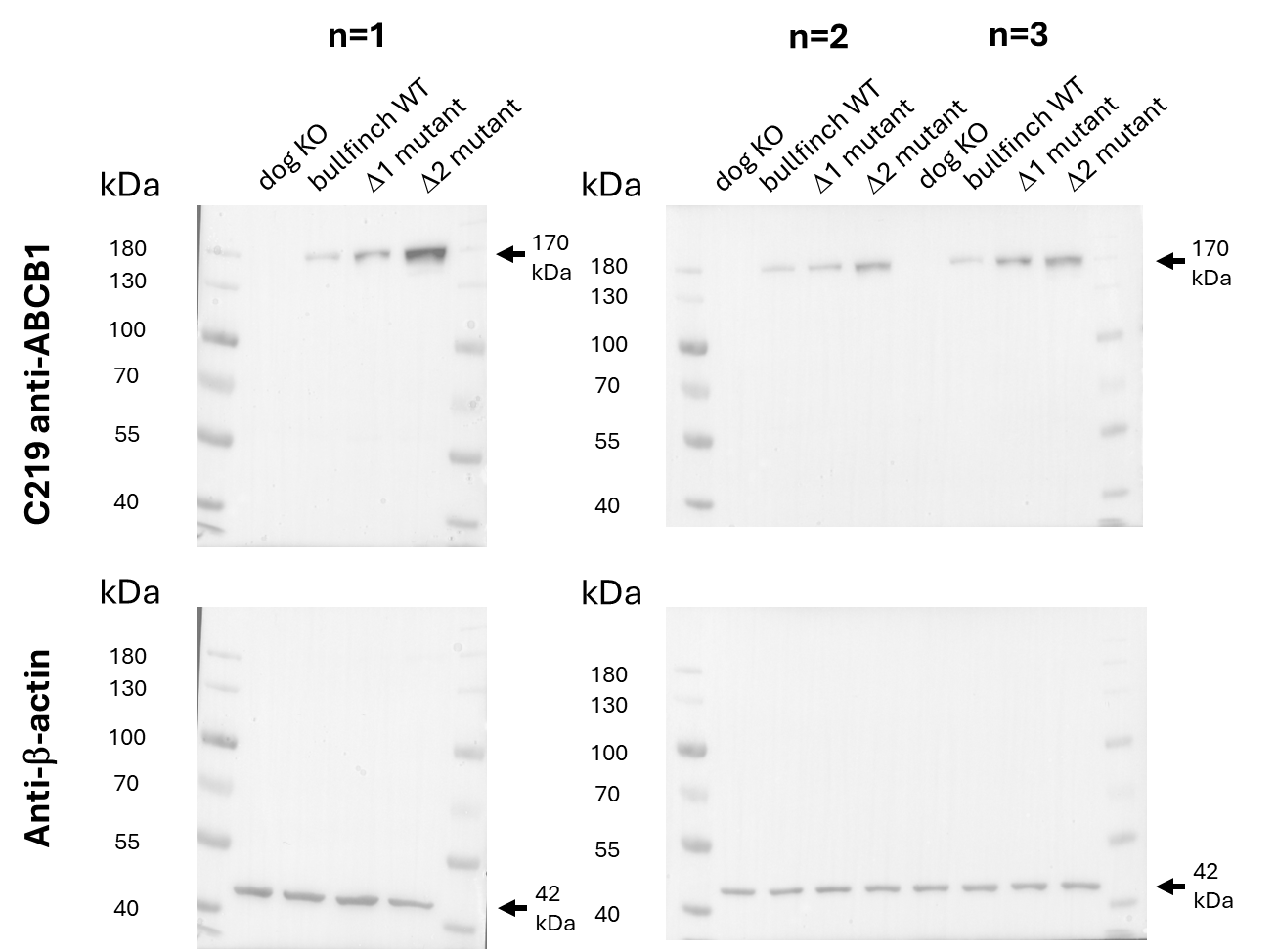
**

**Supplementary Figure 2: Western blot scans.** Scan of n=3 Western blot protein expression analysis of the Eurasian bullfinch ABCB1 transporter proteins (WT, Δ1 mutant and Δ2 mutant) stably expressed in transfected HEK293 cells. Detection of β-actin expression served as the loading control. HEK293 cells stably transfected with the ABCB1 cDNA construct of an ABCB1 mutant dog lacking any ABCB1 protein expression were included as a negative control for Western blotting. The ABCB1 transporter protein of the Eurasian bullfinch revealed an apparent molecular weight of ~170 kDa, while β-actin was detected at ~42 kDa.

**Supplementary Table 1:** Adjusted band volumes of the Western blot analysis.

| **Experiment** | **ABCB1** | **Adjusted volume** | **beta-actin** | **Adjusted volume** | **Ratio** |
| --- | --- | --- | --- | --- | --- |
| 1 | bullfinch WT | 7357290 | bullfinch WT | 38087016 | 0.19317055 |
|  | Δ1 mutant | 22966526 | Δ1 mutant | 42176902 | 0.54452852 |
|  | Δ2 mutant | 96082335 | Δ2 mutant | 28134506 | 3.41510652 |
| 2 | bullfinch WT | 6732504 | bullfinch WT | 17045937 | 0.39496239 |
|  | Δ1 mutant | 10359036 | Δ1 mutant | 20309781 | 0.51005159 |
|  | Δ2 mutant | 26303640 | Δ2 mutant | 17984176 | 1.46259912 |
| 3 | bullfinch WT | 6613248 | bullfinch WT | 18544317 | 0.35661858 |
|  | Δ1 mutant | 24984022 | Δ1 mutant | 19239666 | 1.29856838 |
|  | Δ2 mutant | 35478612 | Δ2 mutant | 21841732 | 1.62434975 |

**Supplementary Table 2:** 95% Confidence intervals for IC_50_ calculation**.**

| **Carrier** | **IC_50_ values (IC_50_) and 95% confidence intervals (95% CI)** | | | |
| --- | --- | --- | --- | --- |
|  | **Tariquidar (TQR)** | | **Ivermectin (IVM)** | |
| dog WT | IC_50_: 0.15 µM  95% CI: 0.14 – 0.16 µM | IC_50_: 0.18 µM  95% CI: 0.17 – 0.19 µM | IC_50_: 2.8 µM  95% CI: 2.7 – 2.9 µM | IC_50_: 3.2 µM  95% CI: 3.4 – 3.7 µM |
| bullfinch WT | IC_50_: 0.11 µM  95% CI: 0.10 – 0.12 µM | IC_50_: 0.13 µM  95% CI: 0.12 – 0.14 µM | IC_50_: 3.9 µM  95% CI: 3.8 – 4.1 µM | IC_50_: 4.1 µM  95% CI: 3.7 – 4.4 µM |
| Δ1 mutant | IC_50_: 0.11 µM  95% CI: 0.10 – 0.13 µM | IC_50_: 0.15 µM  95% CI: 0.14 – 0.16 µM | IC_50_: 2.9 µM  95% CI: 2.5 – 3.3 µM | IC_50_: 3.6 µM  95% CI: 3.3 – 3.9 µM |
| Δ2 mutant | IC_50_: 0.06 µM  95% CI: 0.05 – 0.06 µM | IC_50_: 0.23 µM  95% CI: 0.22 – 0.24 µM | IC_50_: 3.8 µM  95% CI: 3.3 – 4.4 µM | IC_50_: 3.9 µM  95% CI: 3.6 – 4.3 µM |
